# Global, regional, and national burden of kidney dysfunction from 1990 to 2019: a systematic analysis from the global burden of disease study 2019

**DOI:** 10.1186/s12889-023-16130-8

**Published:** 2023-06-23

**Authors:** Shu Zhang, Hui-Fang Ren, Rong-Xin Du, Wei-Li Sun, Mei-Li Fu, Xiao-Chao Zhang

**Affiliations:** Department of Urology, Shanxi Bethune Hospital, Shanxi Academy of Medical Science, Tongji Shanxi Hospital, Third Hospital of Shanxi Medical University, Taiyuan, China

**Keywords:** Disability-adjusted life year, GBD 2019, Kidney dysfunction, Mortality, Summary exposure value

## Abstract

**Objective:**

We aim to explore the prevalence and temporal trends of the burden of kidney dysfunction (KD) in global, regional and national level, since a lack of related studies.

**Design:**

Cross-sectional study.

**Materials:**

The data of this research was obtained from Global Burden of Diseases Study 2019. The estimation of the prevalence, which was measured by the summary exposure value (SEV), and attributable burden of KD was performed by DisMod-MR 2.1, a Bayesian meta-regression tool. The Spearman rank order correlation method was adopted to perform correlation analysis. The temporal trends were represented by the estimated annual percentage change (EAPC).

**Results:**

In 2019, there were total 3.16 million deaths and 76.5 million disability-adjusted life years (DALYs) attributable to KD, increased by 101.1% and 81.7% compared with that in 1990, respectively. From 1990 to 2019, the prevalence of KD has increased in worldwide, but decreased in High-income Asia Pacific. Nearly 48.5% of countries globally, such as South Africa, Egypt and Mexico had increased mortality rates of KD from 1990 to 2019 while 44.6% for disability rate. Countries with lower socio-demographic index (SDI) are facing a higher prevalence as well as mortality and disability rate compared with those with higher SDI. Compared with females, the prevalence of KD was lower in males, however the attributable mortality and disability rate were higher in all years from 1990 to 2019.

**Conclusion:**

With the progress of senescent, we will face more severe challenges of reducing the prevalence and attributable burden of KD, especially in regions with lower SDI. Effective measures are urgently required to alleviate the prevalence and burden of KD.

**Supplementary Information:**

The online version contains supplementary material available at 10.1186/s12889-023-16130-8.

## Introduction

Kidney dysfunction (KD) is defined as a state of decreased kidney function, caused by any conditions which is characterized by metabolite retention, imbalance of water, electrolyte and acid–base metabolism, and systemic symptoms over a period of time and ultimately leads to end stage renal disease (ESRD), dialysis or kidney transplantation [1, 2]. Over the past 40 years, KD has been classified into two distinct syndromes — acute kidney injury and chronic kidney disease (CKD) by nephrologists, both of which are delineated according to the serum creatinine concentration or the glomerular filtration rate (GFR) and important contributor to increased disability rate and mortality for non-communicable diseases. KD has also been identified as a significant risk factor for cardiovascular disease [3] and is a risk multiplier in patients with hypertension and diabetes [4, 5].

Nearly 10% of adults globally are being influenced by a variety of kidney diseases, however, firstly, as a “silent killer”, KD is often hardly detected at its early stage but can cause the lethal kidney failure in its late stage in both developed and developing countries [6, 7], besides, KD is diagnosed through biochemical testing to measure kidney function by estimating GFR and kidney damage through urinary albumin excretion which is not routine tested in daily life by adults, thus, both of above reasons together lead to a phenomenon that patients with KD are often not aware that they are suffering this disease [8]. Furthermore, due to the subdued economic development and health care system function in underdeveloped countries and territories, the prevalence, mortality and disability rate of KD is always underestimated universally [9].

The complexities of characterizing and quantifying the prevalence, mortality disability rate of KD varies across nations, however, previous researches mainly focused on the epidemiology and burden of CKD based on limited data [10], or finite regions [11],the prevalence and attributable burden of KD have not been documented well on a global scale yet, while the mortality and disability rate are significantly higher in KD than those in CKD according to GBD 2019 [12, 13]. The Global Burden of Diseases, Injuries, and Risk Factors Study (GBD) 2019 systematically collected and integrated the risk data from 84 risk factors thus proving a chance to analyze the incidence, prevalence and attributable burden to KD in the global level [14, 15]. KD is defined as a risk factor in the GBD study which includes communicable, non-communicable and malignant diseases. Basing on the data of GBD 2019, the data of summary exposure value (SEV) and attributable burden of KD from 204 countries and territories were extracted for further analyzing the trends of the prevalence and attributable burden of KD from 1990 to 2019 at the global, regional and national levels. We also analysis their association with sex, age and sociodemographic development disparities to provide an up-to-date and comprehensive assessment about the health burden associated with KD and inform priorities for population-level interventions to alleviate the related burden.

## Materials and methods

### Data source and definition

All the data for this study were extracted from the GBD study 2019 (http://ghdx.healthdata.org/gbd-results-tool). The methodology of data inputting, mortality estimation, and modeling for GBD 2019 has been comprehensively reviewed in previously published articles and the final data for every disease, injury, or risk factor can be interpreted in the context of location, year and age groups [12, 13]. In this study, we focused on the prevalence and associated burden of KD from 1990 to 2019 in 204 countries and territories. According to the parent GBD risk factor study, kidney dysfunction is divided into four categories of renal function defined by urinary albumin to creatinine ratio (ACR) and estimated glomerular filtration rate (eGFR): 1. Albuminuria with preserved eGFR (ACR > 30 mg/g & eGFR >  = 60 ml/min/1.73m^2^) which corresponds to stages 1 and 2 CKD in the Kidney Disease Improving Global Outcomes (KDIGO) classification; 2. CKD stage 3 (eGFR of 30–59 ml/min/1.73m^2^); 3. CKD stage 4 (eGFR of 15–29 ml/min/1.73m^2^); 4. CKD stage 5 (eGFR < 15ml/min/1.73m^2^, not (yet) on renal replacement therapy) [13].The input data for estimating the prevalence of KD were based on a systematic review in GBD 2010 on population-based studies, which had been updated for GBD 2013 and 2015. In order to estimate the risk factor, GBD 2019 follows a comparative risk assessment framework which includes six steps: identification of risk outcome pairs; exposure estimation; relative risk (RR) estimation; determination of the theoretical minimum-risk exposure level; and estimation of summary exposure value (SEV) and the attributable burden. The detailed methodology of the modelling and estimation of all risk factors has been specified by previous parent GBD studies [13]. Here we summarized the methods for these steps specific to KD.

### Risk-outcome pairs

Ever since 2010, GBD study has included risk-outcome pairs meeting the World Cancer Research Fund (WCRF) grades of convincing or probable evidence [13]. Cardiovascular diseases, chronic kidney diseases, and gout were regarded as the disease endpoint for KD in GBD 2019.

### Relative risk

The RR to the outcomes has been estimated as a function of exposure to risk factors for each risk-outcome pair in the GBD study. In order to achieve the RR estimation, the GBD study did meta-analyses of RRs from published systematic reviews in each GBD iterations and 81 new systematic reviews was added in GBD 2019. In collaboration with Chronic Kidney Disease Prognosis Consortium (CKD-PC), GBD 2019 got data on 38 new cohorts to estimate the RR for KD, however, none of the 38 studies were added in final model and the original model on a pooled cohort analysis of six cohort studies from CKD-PC in GBD 2017 was continued to be used [13].

### Exposure estimation

Household surveys, censuses, published studies, and governmental data were investigated to estimate the mean levels of risk exposure in order to estimate the distribution of risk exposure. Then, a nonlinear model, spatiotemporal Gaussian process regression (ST-GPR) was applied to estimate the mean exposure along with standard deviation of each risk factor by age, sex, country and year [16, 17]. In that model, age was divided at 0, 10, 20, 30, 40, 50, 60, 70, 80, 90, and 100 years. The time window was set to 10 years for fitting data. The minimum coefficient of variation was 0.1 for global, 0.06 for super regions and 0.08 for other region level.

### Theoretical minimum-risk exposure level

Theoretical minimum-risk exposure level (TMREL) is the theoretically possible risk exposure that minimizes the risk to the exposed population. For kidney dysfunction, the TMREL is ACR 30 mg/g or less and eGFR greater than 60 ml/min/1.73m^2^ [13] calculated basing on the 85th percentile of exposure levels across cohort studies and meta-analyses.

### Population-attributable fractions

Population-attributable fractions (PAF) is defined as the percentage of disease burden that can be decreased if TMREL exposure to a specific risk factor that can be achieved. We calculated the PAF for KD referring to the following formula: $\mathrm{PAF}=\frac{{\int }_{x=l}^{m}RR\left(x\right)dx-RR\left(x\right)TRMEL}{{\int }_{x=l}^{m}RR\left(x\right)P\left(x\right)dx}$, in which l stands for the minimum exposure level while m means the maximum exposure level, RR (x) exists the relative risks at exposure level x, TRMEL stands for the counterfactual exposure level and P(x) is the current exposure level. All variables are calculated according to the combination of covariates, which includes age, sex, location and year.

### Summary exposure values

The prevalence of risk factors was measured by the SEV in GBD 2019, which is weighted by the relative risk, in which the value zero means there exists no excess risk for the population while the value one indicates that the population is facing the highest level of risk. In this study, the weighted prevalence of KD in the global and regional level is presented by the SEV. SEV varies from 0 to 100 in the GBD 2019, while 100 indicating that all the people are at maximum prevalence and 0 indicating that all are at minimum prevalence. In this study, the reported SEV for KD is standardized by age. A decline in age-standardized SEV indicates decreased prevalence of KD, and vice versa.

### Socio-demographic index

The burden of KD was calculated in contradiction of country-level development restrained with the Socio-demographic index** (**SDI) [18], which is a composite indicator that combined by the three following indicators: 1.lag-distributed income per capita; 2. average educational attainment for people aged 15 years and older; 3. the total fertility rate (in people aged < 25 years). The 204 countries and territories were divided into five groups: low SDI (< 0·45), low-middle SDI (≥ 0·45 and < 0·61), middle SDI (≥ 0·61 and < 0·69), high-middle SDI (≥ 0·69 and < 0·80), and high SDI (≥ 0·80) according to the SDI values.

### Statistical analysis

In this study, the age-standardized SEV, mortality rate (ASMR), and disability-adjusted life years (DALYs) rate (ASDR), as well as the 95% uncertain intervals (95% UI) are presented to evaluate and compare the mortality and DALYs rates among countries with distinct age structure and demographic traits and show the epidemiology and burden of KD. The estimated annual percentage change (EAPC) is calculated via age-standardized rates (ASR) in each year from 1990 to 2019 to indicate the trends of age-standardized SEV, ASMR and ASYR with time. All metrics were presented with a 95% uncertainty interval (UI). ASMR and ASDR were reported per 100,000 population. A linear relationship is performed between the natural logarithm of ASMR or ASDR and time, i.e. y = α + βx + ε, where x = year and y = ln(rate) and EAPC is calculated by the following formula: EAPC = 100* (e^β -1) with 95% confidence interval (95% CI). If the lower boundary of 95% CI is positive, ASMR or ASDR is considered to have an upward trend. On the contrary, ASMR or ASDR is deemed to have a downward trend if the higher boundary is negative. Otherwise, ASMR or ASDR is considered to have a stable trend. Gaussian process regression with a Loess smoother is performed to estimate the expected values of SEV, ASMR and ASDR within every SDI unit. Spearman's rank order correlation is used to determine the correlation between the SDI and age-standardized SEV, ASMR and ASDR. Statistical significance is defined as the *p*-value < 0.05. R software (version 4.0.5) is used to perform all statistical analyses.

## Results

### Global and regional prevalence of KD

**Fig. 1:**
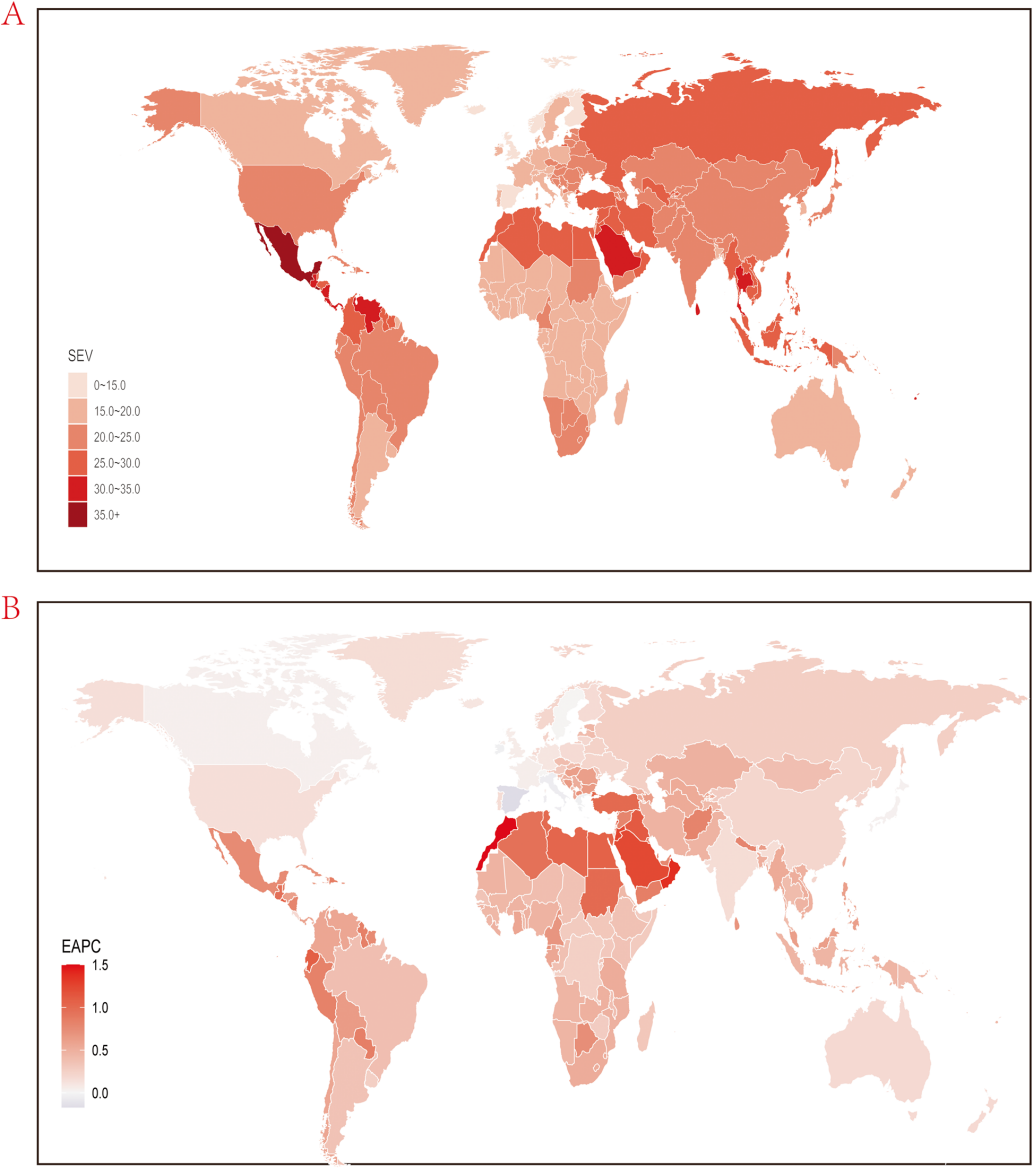
Global exposure to kidney dysfunction. **A** Age standardized SEV of kidney dysfunction, for both sexes in 204 countries and territories in 2019. **B** The EAPC in age standardized SEV of kidney dysfunction, for both sexes from 1990 to 2019, in 204 countries and territories. SEV, summary exposure value; EAPC, estimated annual percentage change

As stated above, the prevalence of KD was measured by age-standard SEV. Table 1 shows the trends of the prevalence of KD in 1990, 2000, 2010 and 2019 at the global and regional level. ﻿In 2019, the global SEVs for KD in both sexes, males and females were 22.74 (95% UI, 16.24 to 30.25), 21.75 (95% UI,15.33 to 29.12) and 23.68 (95% UI, 16.97 to 31.49), respectively. In the region level, the highest age-standard SEV was seen in Central Latin America (35.06; 95% UI, 27.47 to 43.34), following by Southeast Asia (28.11; 95% UI, 20.53 to 36.49), while the lowest was observed in Western Europe (15.59; 95% UI, 10.75to 22.05). In the country level, high age standardized SEV in 2019 were mainly seen in countries located in Central Latin America, Southeast Asia, Southeast Asia and North Africa and Middle East (Fig. 1A). Mexico had the highest SEV (37.11; 95% UI, 29.38 to 45.39), followed by El Salvador and Mauritius. Countries in Western Europe, and central, eastern and western Sub-Saharan Africa had relatively low prevalence of KD and Spain (14.23; 95% UI, 9.6–20.44) had the lowest prevalence among all countries and regions, following by Iceland and United Kingdom (Table S3, Fig. 1A).

**Table 1:** Global and regional age-standardized SEVs of kidney dysfunction for both sexes combined in 1990,2000,2010, and 2019, and EAPC of SEVs from 1990 to 2019 and 1990 to 2019

	**SEV 1990**	**SEV 2000**	**SEV 2010**	**SEV 2019**	**EAPC 1990–2010**	**EAPC 1990–2019**
**Global **	20.56 (14.29to27.97)	21.63 (15.21to29.04)	22.35 (15.82to29.79)	22.74(16.24to30.25)	0.42 (0.4 to 0.44)	0.34 (0.32 to 0.37)
**Gender**						
Male	19.67(13.50to26.86)	20.72(14.36to28.04)	21.43(14.95to28.84)	21.75(15.33to29.12)	0.43 (0.41 to 0.45)	0.35 (0.32 to 0.37)
Female	21.36(14.93to28.92)	22.46(15.97to30.09)	23.20(16.65to30.94)	23.68(16.97to31.49)	0.42 (0.4 to 0.44)	0.35 (0.33 to 0.37)
**SDI**						
High SDI	18.98 (13.66to25.49)	19.99 (14.89to26.12)	21.30 (16.36to27.33)	23.11 (18.17to29.19)	0.15 (0.14 to 0.15)	0.2 (0.18 to 0.23)
High-middle SDI	19.45 (12.88to27.33)	20.7 (14.17to28.39)	22.06 (15.46to29.53)	22.85 (16.51to29.98)	0.39 (0.36 to 0.42)	0.29 (0.25 to 0.32)
Middle SDI	20.06 (12.5to28.92)	21.49009 (13.83to30.42)	23.36034 (15.95to31.86)	24.49285 (17.3to32.51)	0.53 (0.5 to 0.56)	0.41 (0.38 to 0.45)
Low-middle SDI	18.88 (11.63to27.48)	19.69 (12.41to28.31)	20.73 (13.53to29.29)	21.38 (14.23to29.7)	0.41 (0.38 to 0.44)	0.31 (0.28 to 0.34)
Low SDI	15.66 (9.05to24.02)	16.03 (9.26to24.63)	16.47 (9.57to25.2)	17.03 (10.07to25.49)	0.38 (0.37 to 0.38)	0.36 (0.35 to 0.36)
**Region**						
Andean Latin America	16.87(11.25to23.96)	18.35(12.61to25.62)	19.83(13.77to27.07)	21.21(14.97to28.73)	0.81 (0.81 to 0.82)	0.79 (0.78 to 0.8)
Australasia	16.47(11.65to22.93)	16.75(11.92to23.38)	17.02(12.15to23.66)	17.37(12.39to23.97)	0.17 (0.17 to 0.17)	0.18 (0.17 to 0.18)
Caribbean	20.12(13.66to27.79)	22.21(15.53to29.95)	23.73(16.83to31.48)	24.7(17.86to32.45)	0.83 (0.79 to 0.87)	0.7 (0.66 to 0.74)
Central Asia	20.84(13.99to28.69)	21.55(14.67to29.52)	22.66(15.62to30.82)	24.3(17.12to32.55)	0.43 (0.41 to 0.45)	0.52 (0.49 to 0.55)
Central Europe	17.9(11.96to25.08)	18.85(12.86to26.01)	19.67(13.6to26.81)	20.31(14.13to27.55)	0.48 (0.46 to 0.49)	0.43 (0.42 to 0.45)
Central Latin America	28.62(20.93to36.68)	31.4(23.7to39.64)	33.56(26.03to41.84)	35.06(27.47to43.34)	0.8 (0.77 to 0.83)	0.69 (0.66 to 0.73)
Central Sub-Saharan Africa	15.01(9.29to22.07)	15.25(9.53to22.35)	15.67(9.92to22.84)	16.57(10.69to23.55)	0.22 (0.21 to 0.23)	0.31 (0.28 to 0.35)
East Asia	20.87(14.2to28.88)	21.99(15.18to29.99)	22.42(15.53to30.42)	22.21(15.41to30.16)	0.36 (0.32 to 0.4)	0.21 (0.17 to 0.26)
Eastern Europe	22.61(15.51to30.72)	22.97(15.83to31.12)	23.54(16.34to31.67)	24.57(17.33to32.76)	0.21 (0.2 to 0.22)	0.27 (0.25 to 0.29)
Eastern Sub-Saharan Africa	15.19(9.49to22.15)	15.67(9.95to22.63)	16.28(10.42to23.23)	17.01(11.11to24.22)	0.35 (0.34 to 0.36)	0.39 (0.37 to 0.4)
High-income Asia Pacific	20.79(15.11to28.11)	20.61(14.94to27.86)	20.55(14.88to27.81)	20.69(14.94to27.81)	-0.05 (-0.06 to -0.05)	-0.02 (-0.03 to -0.01)
High-income North America	20.36(14.62to27.08)	20.76(14.86to27.67)	20.98(15.1to28.01)	21.36(15.47to28.4)	0.15 (0.14 to 0.16)	0.15 (0.14 to 0.15)
North Africa and Middle East	20.33(14.55to27.38)	23.31(17.23to30.52)	25.44(19.08to33.08)	26.9(20.45to34.56)	1.13 (1.07 to 1.19)	0.94 (0.88 to 1)
Oceania	23.23(16.02to31.36)	24.07(16.72to32.33)	24.96(17.48to33.37)	25.91(18.39to34.34)	0.37 (0.37 to 0.37)	0.37 (0.37 to 0.38)
South Asia	21.6(14.71to29.51)	22.96(15.89to30.85)	23.49(16.41to31.42)	22.93(15.91to30.9)	0.41 (0.36 to 0.46)	0.22 (0.16 to 0.28)
Southeast Asia	24.16(16.9to32.42)	25.47(18.14to33.75)	26.8(19.31to35.31)	28.11(20.53to36.49)	0.53 (0.52 to 0.53)	0.52 (0.52 to 0.52)
Southern Latin America	17.15(11.74to23.88)	18.37(12.94to25.17)	19.1(13.61to26.01)	19.38(13.88to26.45)	0.54 (0.51 to 0.58)	0.42 (0.38 to 0.46)
Southern Sub-Saharan Africa	19.04(12.85to26.5)	19.58(13.23to27.07)	20.55(14.02to28.17)	22.17(15.5to29.72)	0.39 (0.37 to 0.42)	0.51 (0.47 to 0.55)
Tropical Latin America	18.9(12.99to26.17)	19.5(13.6to26.85)	20.24(14.34to27.61)	21.24(15.16to28.66)	0.35 (0.34 to 0.36)	0.39 (0.38 to 0.41)
Western Europe	15.28(10.43to21.69)	15.07(10.55to21.1)	15.09(10.61to21.11)	15.59(10.75to22.05)	-0.06 (-0.07 to -0.04)	0.05 (0.01 to 0.08)
Western Sub-Saharan Africa	15.72(10.2to22.71)	16.45(10.85to23.46)	17.22(11.53to24.23)	18.12(12.27to25.06)	0.46 (0.46 to 0.46)	0.48 (0.47 to 0.49)

*SEV* Summary exposure value, *EAPC* Estimated annual percentage change

In order to explore the trends of the risk exposures of KD, we further analyze the trends in two time-intervals: the full duration of the study, 1990-2019, and the past decade, 2010-2019. We found that the trends were comparable in these two time-intervals. At the global level, the EAPC of SEVs for both sexes, males and females were 0.34 (95% CI, 0.32 to 0.37), 0.35 (95% CI,0.32 to 0.37) and 0.35 (95% CI, 0.33 to 0.37), respectively in 1990-2019 while the EAPC of SEVs for both sexes, males and females were 0.42 (95% CI, 0.4 to 0.44), 0.43 (95% CI,0.41 to 0.45) and 0.42 (95% CI, 0.4 to 0.44), respectively in 1990-2010. Compared with EAPC for SEV for KD in 1990-2010, a silent decrease was seen in 2010-2019, however, it’s note-worthy that although the increasing trend of SEVs for KD has slowed recently, these still observes an obviously increasing trend globally (Table 1, Figure 1B). From 1990 to 2019, all regions showed an increase of SEV for KD except for High-income Asia Pacific, while the highest increase was seen in North Africa and Middle East (0.94; 95% CI, 0.88 to 1), following by Andean Latin America. There exists a little difference between the trends within the past decade and within the past thirty years at the regional level. Comparing the EAPC of SEV between two durations, it is noting that 10 regions had higher EAPCs of KD while 12 regions had lower EAPCs from 2010 to 2019 than 1990 to 2019. (Table 1) Trends of SEV for these three risk factors from 1990 to 2019 globally were shown in Figure 1B, most countries showed increasing trends within the past thirty years except for Greece, Ireland, Republic of Korea, Italy, Singapore and Spain. It should be noted that 14 countries are facing a EAPC value higher than 1.0 for KD and the highest was observed in Morocco (1.5; 95% CI, 1.47 to 1.53) (Table 3S, Figure 1B).

### Risk-attributable burden

**Fig. 2:**
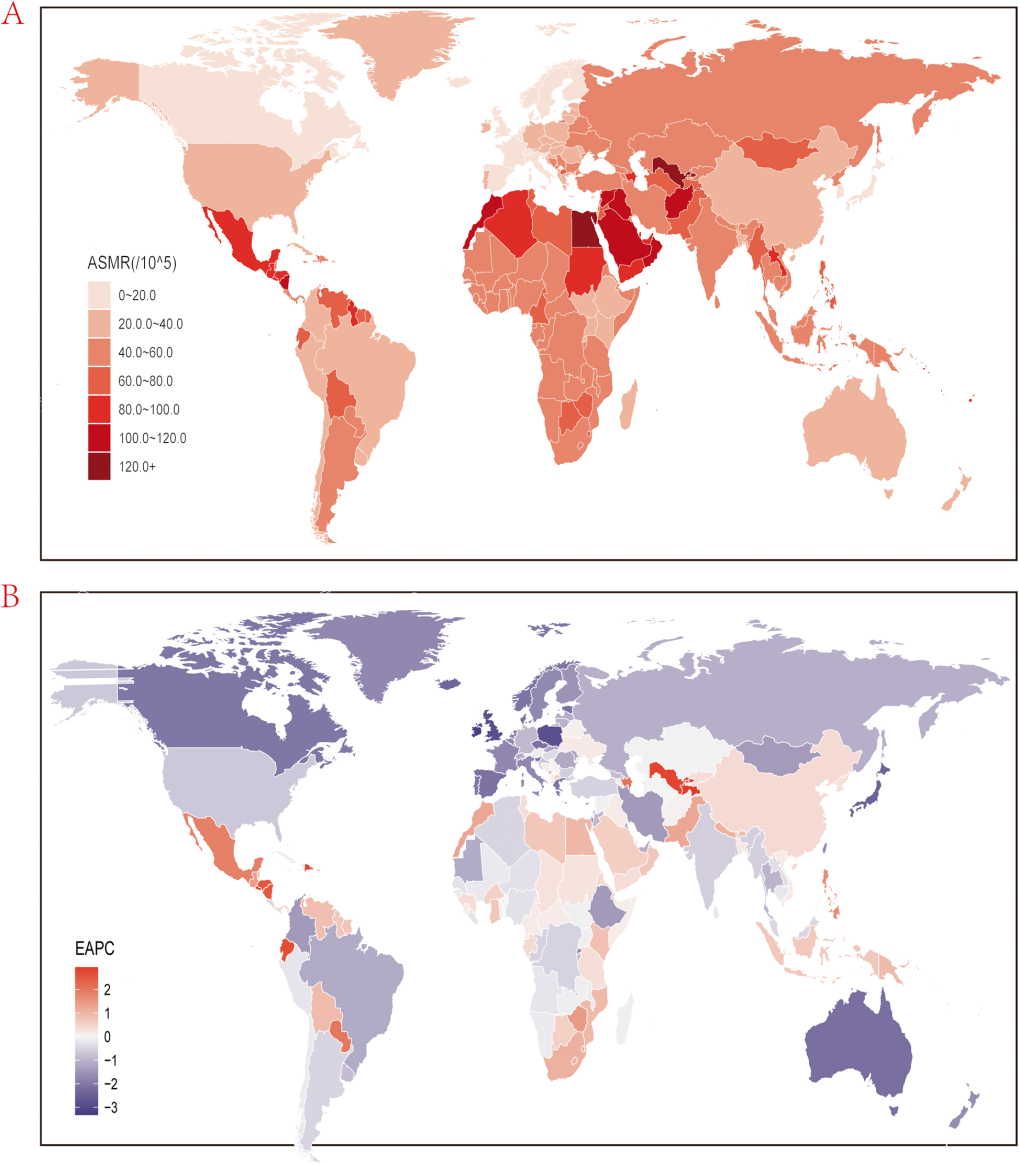
Global age standardized mortality rate to kidney dysfunction. **A** The all-cause ASMR per 100,000 associated with kidney dysfunction, for both sexes in 204 countries and territories in 2019. **B** The EAPC of ASMR of kidney dysfunction, for both sexes from 1990 to 2019, in 204 countries and territories. ASMR, age standardized mortality rate; EAPC, estimated annual percentage change

Globally in 2019, there were 3,161,551.84 (95% UI, 2,723,362.52 to 3,623,813.85) deaths due to KD, and there observed an increase of nearly 101.1%. The age-standardized mortality rate (ASMR) due to KD was 40.64 (95% UI, 34.81 to 46.71) per 100,000 population, which has decreased (EAPC, -0.18;95% CI, -0.26 to -0.11) and(EAPC, -0.35; 95% CI, -0.41 to -0.29) in 1990-2010 and 1990-2019. In the regional level, North Africa and Middle East had the highest ASMR (83.4; 95% UI, 69.82 to 97.32) for KD, while High-income Asia Pacific had the lowest ASMR for KD (15.94; 95% UI, 12.93 to 18.54). Concerning the trends from 1990 to 2019, Southern Sub-Saharan Africa had the highest increase ASMR for KD in 1990-2010 (2.01; 95% CI, 1.56 to 2.46) and Central Latin America had the highest in 1990-2010 (1.24; 95% CI, 1.1 to 1.38) while the greatest decreases were seen in High-income Asia Pacific both in 1990-2010 and 1990-2019 (Table 1S). Considerable global variation of more than 10-fold was observed in ASMR for KD among countries, with the highest in Nauru (146.46; 95% UI, 117.96 to 179.83) and the lowest in San Marino (12.49; 95% UI, 8.37 to 17.18) (Figure 2A, Table 4S). It’s notable that Figure 2B showed the trends of ASMR for KD in universally and over a half of countries showed decreasing trends. Republic of Korea had the most decreasing of ASMR for KD in both 1990-2010 (-3.78; 95% CI, -3.97 to -3.59) and 1990-2019 (-3.32; 95% CI, -3.5 to -3.14), meanwhile, the highest of ASMR for KD increase was seen in Uzbekistan (4.59; 95% CI, 4.07 to 5.11) in 1990-2010 while El Salvador (2.94; 95% CI, 2.5 to 3.37) had the highest increase in 1990-2019.

**Fig. 3:**
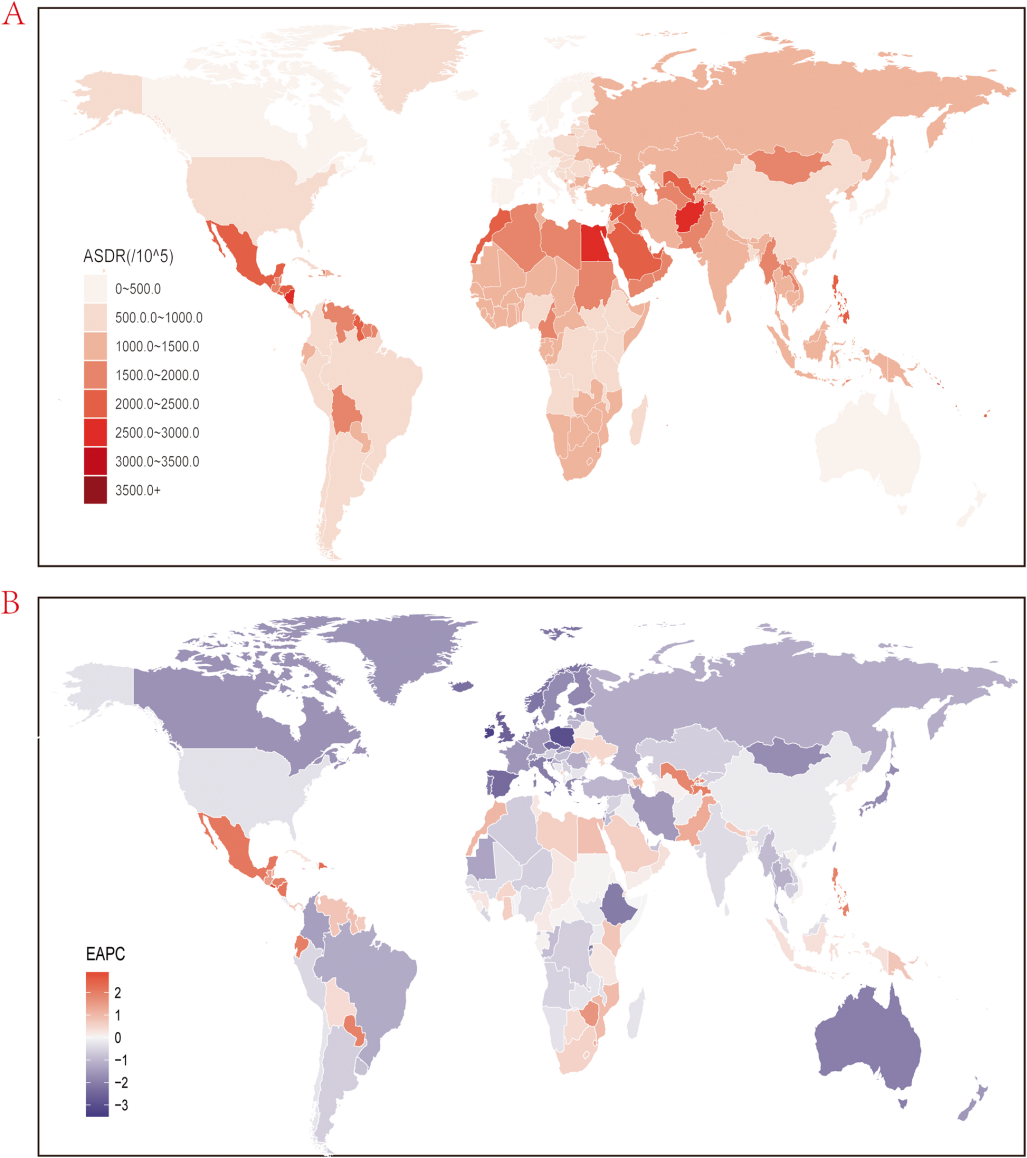
Global age standardized DALYs rate of kidney dysfunction. **A** The all-cause ASDR per 100,000 associated with kidney dysfunction, for both sexes in 204 countries and territories in 2019. **B** The EAPC of ASDR of kidney dysfunction, for both sexes from 1990 to 2019, in 204 countries and territories. DALYs, disability-adjusted life years. ASDR, age standardized DALYs rate; EAPC, estimated annual percentage change

There was total 76,486,944.94 (95% UI, 67,791,320.41 to 86,284,798.38) DALYs due to KD, increased by 81.7% compared to that in 1990. The age-standardized DALYs rate (ASDR) due to KD was 945.31 (95% UI, 836.33 to 1066.77) per 100,000 population, which has decreased (EAPC, -0.25; 95% CI, -0.31 to -0.19). In region level, the highest of ASDR for KD was seen in Central Latin America (1781.44; 95% UI, 1565.02 to 2028.72), following by North Africa and Middle East and Oceania while Western Europe had the lowest ASDR (327.1; 95% UI, 286.04 to 372.3). Similar to ASDR, the largest increase of ASDR was also observed in Central Latin America (EAPC, 1.41; 95% CI, 1.26 to 1.56), however, High-income Asia Pacific rather than Western Europe had the most decline of ASDR for KD (EAPC, -2.14; 95% CI, -2.2 to -2.08) (Table 2S). As stated in Fig. 3A and Table 5S that demonstrated the ASDR for KD in country level, the highest ASDR was seen in Nauru (3869.29; 95% UI, 3112.94 to 758.6), following by Micronesia (Federated States of) and Kiribati while San Marino had the lowest ASDR (225.42; 95% UI, 166.53 to 298.56). The highest increase of ASDR for KD was observed in El Salvador (EAPC, 2.9; 95% CI, 2.45 to 3.34) while Republic of Korea posed the greatest decrease (EAPC, -3.52; 95% CI, -3.7 to -3.34) (Fig. 3B, Table 5S).

### Causes of KD-related mortality and disability

For the 87 risk factors in GBD 2019, each risk factor is associated with an outcome or outcomes, defined as risk-outcome pairs [13]. There are three causes for KD-related mortality and four causes for disability (Figure S1). In all years from 1990 to 2019, CKD had surpassed ischemic heart diseases (IHD) to be the most important cause associated with the highest ASMR and remained the top cause of ASDR, followed by IHD and stroke for ASMR while IHD, stroke and gout for ASDR. (Figure S1). During this period, the ASMR associated with CKD and IHD fluctuated around 18 deaths per 100,000, and the ASDR was over 300 DALYs per 100,000 in most years. There was a gradual decrease from 1990 to 2019 in the ASMR and ASDR for IHD and stroke however an obvious increase for CKD, as for gout in ASDR, the trend remained relatively stable.

### Correlation of SEV and attributable burden with SDI

**Fig. 4:**
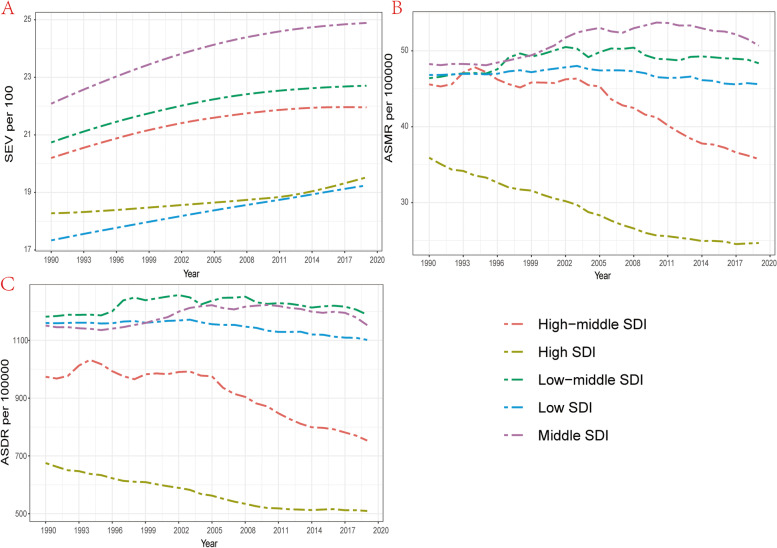
The exposure and burden of kidney dysfunction by SDI. **A** The age standardized SEV, **B** ASMR and **C** ASDR of kidney dysfunction in different SDI regions from 1990 to 2019. Results are showed for both sexes in worldwide. SEV, summary exposure value; ASMR, age standardized mortality rate; DALYs, disability-adjusted life years. ASDR, age standardized DALYs rate

Figure 4 and Figure S3 showed the age-standardized SEV as well as mortality and DALYs rate from 1990 to 2019 in all GBD super regions. It’s noteworthy that high SDI and low SDI regions are facing a lower SEV compared with middle, high-middle and low-middle SDI regions and the highest SEV is seen in middle SDI regions while ASMR and ASDR exhibit a different trend. Regions with high and high-middle SDI are facing a comparable lower ASMR and ASDR per 100000, while regions with lower SDI value have larger ASMR and ASDR. Since 1990, the SEV of all regions increased gradually, however, only high and high-middle SDI regions experienced a downtrend of ASMR and ASDR while ASMR and ASDR in regions with lower SDI fluctuated or ever slightly increased in the past thirty years (Table 1S and 2S, Figure 4B and C). A generally increasing trend was observed in SEV of all seven other GBD super regions, North Africa and Middle East had the fastest increasing SEV as well as the highest ASMR and ASDR from 1990 to 2019 in this seven GBD super regions while High-income posed a relatively stable SEV as well as the lowest ASMR and ASDR, which showed a trend of general decrease (Figure S3). However, unlike the other six regions, there has been a downward trend of SEV of South Asia from 2010 to 2019 (Figure S3A).

**Fig. 5:**
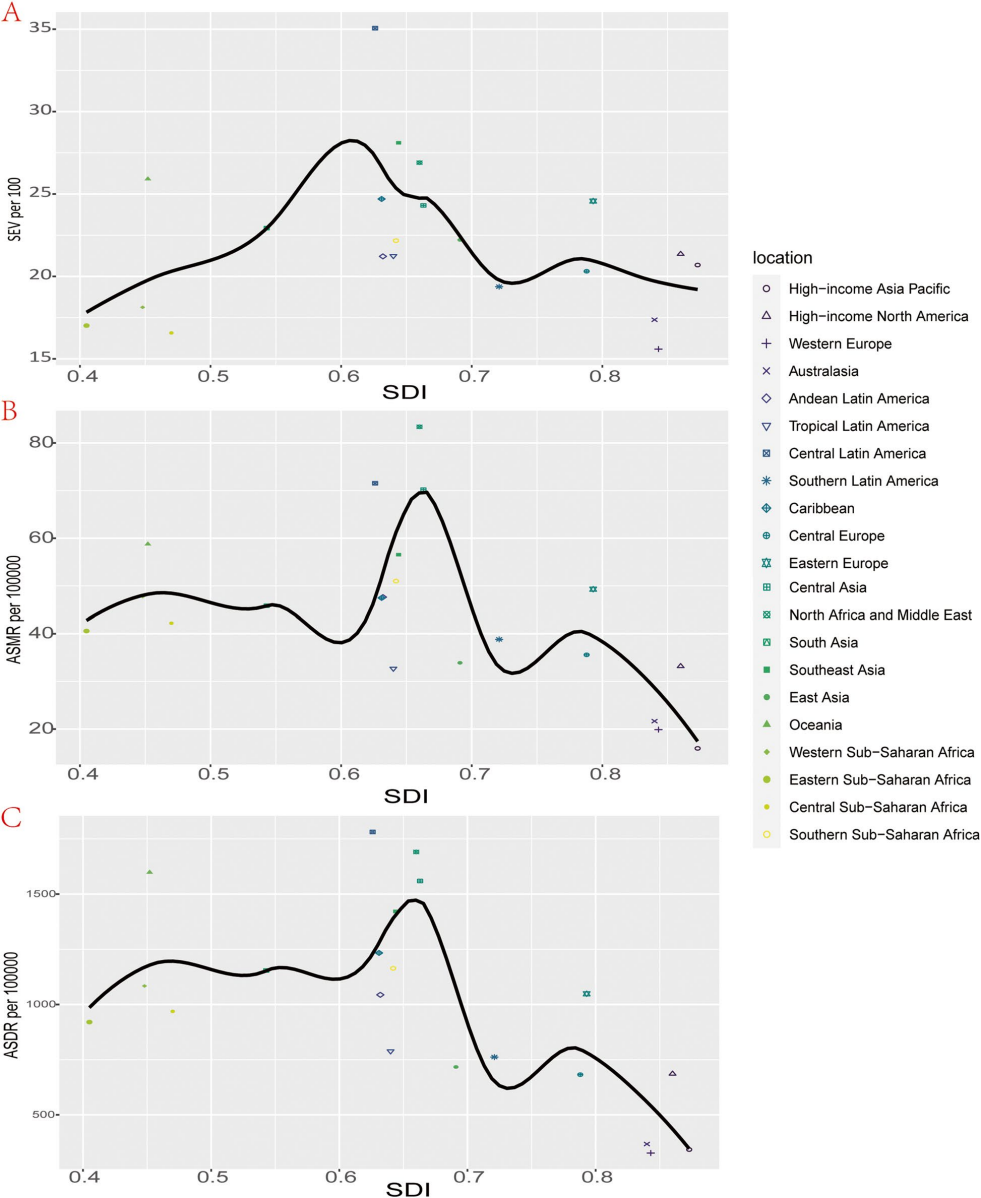
Correlations of SEV, ASMR as well as ASDR and SDI at the regional level. Age-standardized SEV (**A**), ASMR (**B**) as well as ASDR (**C**) for kidney dysfunction and SDI at the regional level in 21 regions from 1990 to 2019. SEV, summary exposure value; ASMR, age standardized mortality rate; DALYs, disability-adjusted life years. ASDR, age standardized DALYs rate

Concerning the correlation with SDI, an overall negative association was observed in SEV for KD as well as ASMR and ASDR (Figure 5, Figure S2). In the region level, Central Latin America, Eastern Europe, Southeast Asia and North Africa and Middle East posed a higher observed SEV for KD than the expected trends based on SDI over the observed period, while the SEV of Western Europe, Tropical Latin America, Andean Latin America and Australasia were below the expected value over this period. Similar with SEV, the general trends of ASMR and ASDR demonstrated that Central Latin America, Eastern Europe, High-income North America and North Africa and Middle East had a higher ASMR and ASDR than expected while the ASMR and ASDR of Western Europe, Tropical Latin America, Tropical Latin America, Andean Latin America and Australasia significantly stayed below the expected value (Figure 5). The trends of observed SEV, ASMR and ASDR versus the expected level based on their SDI values at the national level were comparable to the regional level, in which Mexico posed the biggest difference from the expected value of SEV for KD while Nauru had both the largest difference for ASMR and ASDR (Figure S2).

### Age and sex patterns

**Fig. 6:**
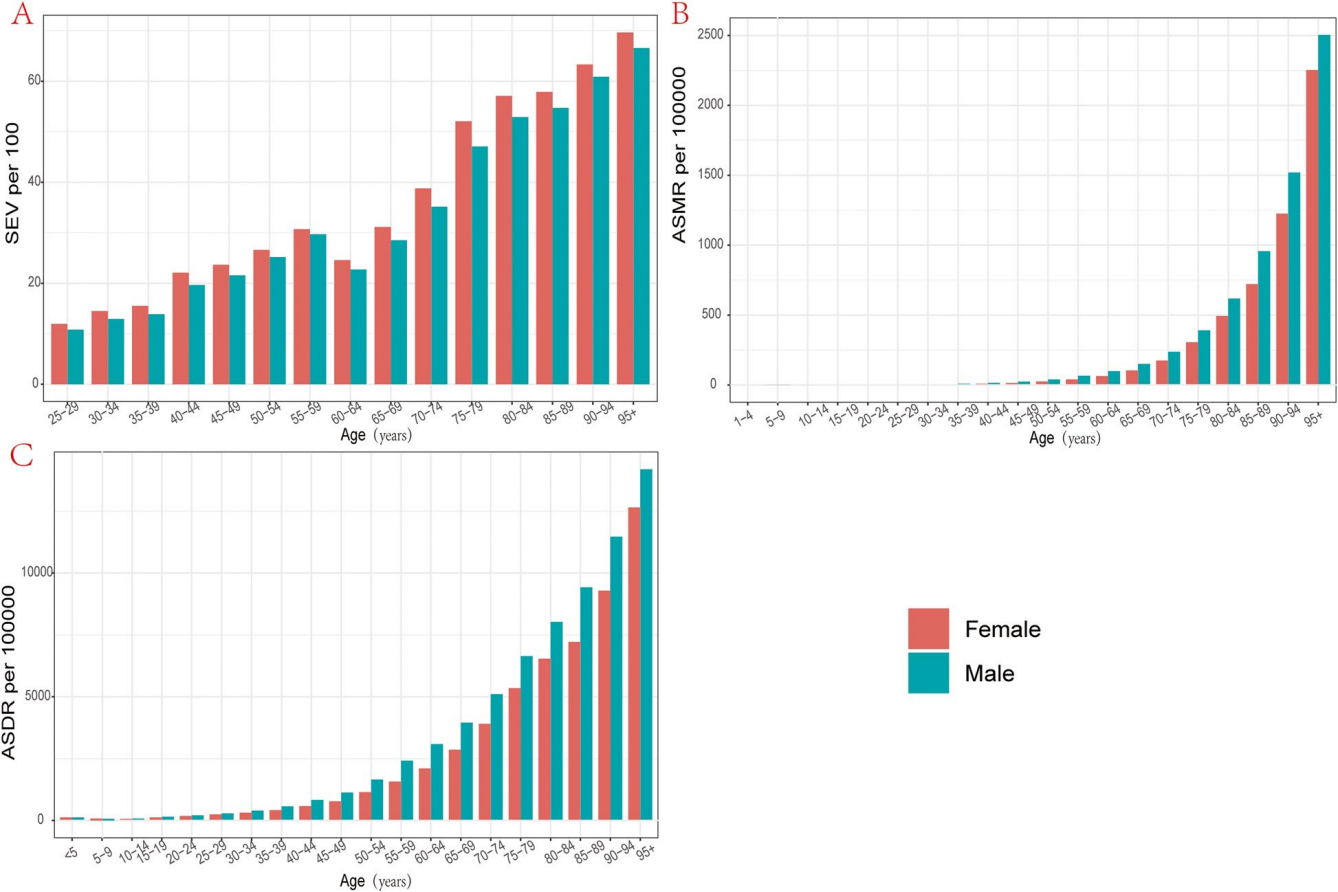
The exposure and burden of kidney dysfunction by age and sex. The all-cause (**A**) age standardized SEV, (**B**) ASMR and (**C**) ASDR of kidney dysfunction in worldwide in different age groups. SEV, summary exposure value; ASMR, age standardized mortality rate; DALYs, disability-adjusted life years. ASDR, age standardized DALYs rate

Compared with males, females had a higher prevalence rate however a lower mortality rate and DALYs rate for KD in all years from 1990 to 2019 and the gap between genders showed a generally stable trend in the past thirty years. Although males had lower SEVs for KD, the mortality rate was approximately 40% higher and the DALYs rate was 25% higher for males than females in 2019 (Figure S4). The data on the age distribution of SEV for KD were only available for those aged 25 years or older﻿ while the data of ASMR and ASDR in all ages were available in GBD 2019. In generally, females were facing a higher SEV but lower mortality rate and DALYs rate in all age groups. The elderly was facing a higher SEV and larger burden of KD compared with the younger and the growing speed trend of ASMR and ASDR of KD increased rapidly in those older than 64 years old. However, it’s noting that although a generally increasing trend of SEV was seen in this study, there still exists an urgency decreasing trend in those aged 60 to 64 years old and then keep on increasing as well as the general trend (Figure 6). There was a generally increasing trend of ratio of male to female SEV, mortality rate and DALYs rate before 60 years old, however, the trend of SEV differs from those of mortality rate and DALYs rate in 60+ age group. As for SEV of those age 60 years old or older, there exists a rapidly decline trend before 80 years old however an increasing trend in those aged 80 to 90 years old then decreased in 95+ age group. A decreasing trend was observed for ASMR and ASDR of those age 60 to 90 years old with a reverse trend only occurs in 80 to 84 years old (Figure S5).

## Discussion

In this study, we find that the global prevalence of KD has increased in the past thirty years, conversely most countries have decreasing trends of KD attributable burden. However, there exists large variations in the prevalence and attributable burden of KD across countries and territories, what’s noteworthy is that many countries, mainly location in low and low-middle SDI regions, had increasing burden from 1990 to 2019. Females are facing a higher SEV but a lower attributable burden compared with males and the gender gap is particularly in those older than 60 years old. The SDI was negatively correlated with SEV, ASMR and ASDR, indicating that low SDI regions had higher exposure levels and attributable burden of KD.

The increasing prevalence trend of KD in almost all regions and nations might be a result of the high-speed increasing population from 1990 to 2019 globally [12], however, the increase in the global age-standardized SEV for KD from 1990 to 2019 was not accompanied by corresponding increases in age-standardized mortality rate and disability rate, indicating high prevalence of non-fatal KD and the development and universality of renal replacement treatment of KD have achieved desirable outcomes, such as kidney transplantation, peritoneal dialysis and hemodialysis. In addition, we observed that the SEV, ASMR and ASDR for KD are all negatively correlated with SDI, suggesting that people in low-income countries are facing a higher possibility to KD than developed countries. Such correlation may be explained by lacking of health awareness, the low rate of routine medical examinations as well as the lack of medical resource and experienced nephrologists, we should note that not all patients with KD could receive renal replacement therapy, especially in developing countries, it is reported that 78% of patients with KD lived in low-income and middle-income countries, where resources, availability of dialysis, and kidney transplantations were unpleasant [19]. Although in nations with higher development level, the medical resource might be adequate enough to supply the need of renal treatment of KD, we should note that there is also a high age-standard SEV, ASMR and ASDR in high SDI nations such as America, Germany and Russia which leads to an increased burden of medical financial expenditure and financial working of these countries. Thus, there is an urgent need for all countries to pay more attention to KD and accelerate the process of prevention projection of KD in worldwide. Besides, in order to promoting the prevention of KD, the treatment of CKD also remains a big challenge in low-SDI countries, because the mortality and disability rate were also higher in these countries.

Developing status is a significant factor contributing to the age-standard SEV, ASMR and ASDR, compared regions with high and low SDI, high-middle, middle and low-middle SDI regions are facing a significantly larger SEV for KD, which might be explained by the limited number of populations in low SDI regions and the high medical care levels in high SDI regions, and nations with modest SDI had a higher increasing speed of population compared with low and high SDI regions. As for ASMR and ASDR, regions with higher SDI showed considerably lower value compared with those with lower SDI, which might because that people in higher-SDI regions have more opportunities to benefit from a well-developed healthcare system and policy priorities [17], besides, prolonged life expectancy and increased percentage of elderly people in high SDI regions may play a vital role, because that KD is an age-related condition which mainly affects the elderly [20]. Although all SDI regions exhibited an increasing trend of SEV for KD, the EAPC of SEV was negatively correlated with SDI, furthermore, the ASMR and ASDR fluctuated rather than reducing in lower SDI regions, both indicating the urgency of prevention and treatment of KD in all SDI regions especially in lower SDI regions. The findings at the national level were comparable to the regional level. From 2010 to 2019, there was a global decrease in ASMR and ASDR for KD. However, the situation of KD prevention and control varied from countries. Although most countries had decreased KD attributable burden, there saw upward trends in many countries, mainly in underdeveloped countries, such as Tajikistan, Uzbekistan, El Salvador, and Mexico. What’ more, it’s noteworthy that during the past thirty years, as the rate decreased globally, the absolute number of deaths and DALYs attributable to KD increased in almost all the countries, caused by the increasing population and the process of senescent universally, which will certainly upsurge the burden of medical and economy of the society, and greater challenges will come if efforts are not fully performed to deal with KD and CKD globally.

GBD 2019 defined IHD, CKD, stroke and gout as outcomes associated with KD, which might be able to explain why the mortality and disability of KD are significantly higher than those of CKD. IHD was once the most common outcome of ASMR for KD, however the ASMR and ASDR attributable to IHD has decreased in the past thirty years continuously. Reasons for such results are complicated, the invitation and update iterations of prevention medications permit the declining mortality rate and DALYs rate [21], in addition, the most important risk factor attributable to IHD was High systolic blood pressure, following by High LDL cholesterol and High fasting plasma glucose [22]. In addition, because of the same degree of progress in prevention mortality rate and DALYs rate of CKD as we have seen for many other important non-communicable diseases were not observed, a limited decline for CKD (2·8% change [95% UI − 1·5 to 6·3]) from 1990 to 2017 [10], resulting that CKD the ASMR and ASDR attributable to CKD continuously from 1990 to 2019 and had surpassed IHD to be the leading outcome of ASMR for KD and kept on the top outcome of ASDR for KD. The prevalence and attributable burden of stroke for KD was comparable with that of IHD.

Compared with females, despite lower level of exposure, males possessed higher ASMR and ASDR for KD, as evidenced by a 40% higher mortality rate and a 25% higher DALYs rate, however the gender difference are becoming more and more narrow in the past thirty years, which was in accordance with previously reported studies [23, 24]. The contribution of sex in different age groups was as similar as that of all ages however the gender difference of ASMR and ASDR held a different trend, which is particularly marked in females up to the age of 59 years, nevertheless this gender advantage is abruptly weakened while after 59 years, reflecting the remarkable protective effects of estrogen on the cardiovascular system prior to menopause [25]. People aged 70 + years make up the majority of IHD-related mortality while the majority of CKD-related mortality was mainly observed in those aged 60 + years old [10, 26], which was accordance with our results, we reported that the age-standard SEV, ASMR and ASDR were significantly higher in the elderly than the youngers, indicating the priority and urgency in preventing KD and KD-related outcomes, such as CKD, IHD and stroke, of elderly population.

Although the effect of KD on the burden of non-communicable diseases was not just limited in CKD and ESKD, it is reported that almost 7% total cardiovascular disease burden could be attributed to KD, it is reasonable to prevent severe adverse events and reduce mortality rate and DALYs rate due to KD in people at high risk and early population by routine examination of kidney function and standard treatment [27]. Previous studies has demonstrated that screening for CKD in both high-risk and early stage populations is a cost-effective method to delay the progression to CKD and ESKD [28, 29]. Dialysis plays a vital role in the treatment of CKD in the past decades, however, due to the limitation of health education and medical resource, poor adherence to dialysis treatment, the delay of treatment of primary disease, such as diabetes, hypertension and glomerulonephritis, fear of adverse effects and economic burden had resulted in insufficient treatment of KD, leading to the high ASMR and ASDR in worldwide especially in regions with low SDI [30, 31]. Peritoneal dialysis exists as the commonest measure of treatment of KD while peritonitis continues to be the major reason of mortality rate and DALYs rate in patients receiving peritoneal dialysis globally [32], however, as a preventable complication, there is plentiful evidence that the rate of peritonitis around the world have decreased considerably and the vast majority with KD would receive benefits from proper treatment of dialysis [33]. Public health also plays a vital role in decreasing the steadily rising rate of KD and CKD even ESKD by personal education of health, routine kidney function detection programmes, early administration of renal protective therapy and appropriate treatment of primary disease that affects kidney function [34]. In addition to dialysis and other renal replacement therapy, the access to laboratory diagnostic services, the awareness of KD treatment of health workers, medical consulting to patients and public health education on the harm of KD are all not sufficient enough universally especially in underdeveloped countries [35, 36], thus, more attention need to be paid to KD by policy-makers and more health education and supportive policies are in great urgency to alleviate the severe situation of KD.

To the best of our knowledge, this is the first study to explore the prevalence health burden of KD, stratified by age, sex and sociodemographic development thus providing a comprehensive description on it. However, there also exists some limitations that could not be ignored in our study. First of all, as suggested by KDIGO guidelines, repeat serum creatinine and urine ACR measurements over 3 months are required to confirm the chronicity of abnormalities [37], however, most involved studies reporting the prevalence of non-fatal CKD in GBD 2019 failed to provide such results and the nature of cross-sectional of these studies both result into a possible 25–50% overestimation of prevalence of CKD [38]. Hence, it is possible that an overestimation of KD prevalence was presented by the results of our analysis. The modeling of this study also leads to some internal limitations: Firstly, GBD 2019 failed to contain Mendelian randomisation studies in meta-regression, which might provide some unexpected new insights; Secondly, the data source ﻿effective size of KD on the outcome were mostly obtained from prospective observational studies and the authenticity and reliability of the estimates will be reduced by confounding factors in prospective observational studies. Besides, relative risks are often assumed as a function of exposure that are universal and consistent across regions and time periods, which will lead to inevitable bias [13].Due to the insufficient development in less-developed countries, high-quality primary data are sparsely acquired in these countries, nevertheless GBD must rely on statistical methods and predictive covariate values to generate final estimates. Furthermore, even when the data are available, the discrepancies of data in terms of quality, accuracy and comparability might also result into the deviations in the final estimated values [12, 13, 26].

## Conclusion

Despite the fact that the high prevalence and attributable burden of KD, increasing prevalence however decreasing attributable burden had also been observed globally from 1990 to 2019. ﻿However, some countries, including many high-income countries, remains suffering from increasing trends of age-standard SEV, ASMR and ASDR for KD. In general, regions and nations of lower SDI are facing a higher prevalence and mortality and disability rate of KD. Compared with females, males have a lower prevalence however higher attributable burden of KD, the elderly are facing higher age-standard SEV, ASMR and ASDR for KD. With the progress of senescent, we will face more severe challenges of KD and this study provided an evidence-based guidance for policymakers to design and implement appropriate public health measures and interventions to alleviate the burden associated with KD.

## Supplementary Information

**Additional file 1:** **Figure S1.** The top three causes for KD-related outcomes of ASMR (A) and top four causes of ASDR (B) in worldwide for both sexes from 1990 to 2019. ASMR, age standardized mortality rate; DALYs, disability-adjusted life years. ASDR, age standardized DALYs rate.

**Additional file 2:** **Figure S2.** Correlations of SEV, ASMR as well as ASDR and SDI at the national level. Age-standardized SEV (A), ASMR (B) as well as ASDR (C) for kidney dysfunction and SDI at the regional level in 204 countries and territories from 1990 to 2019. SEV, summary exposure value; ASMR, age standardized mortality rate; DALYs, disability-adjusted life years. ASDR, age standardized DALYs rate; SDI, sociodemographic index.

**Additional file 3:** **Figure S3.** The exposure and burden of kidney dysfunction in seven GBD super regions. (A) The age standardized SEV, (B) ASMR and (C) ASDR of kidney dysfunction in different SDI regions from 1990 to 2019. Results are showed for both sexes in worldwide. SEV, summary exposure value; ASMR, age standardized mortality rate; DALYs, disability-adjusted life years. ASDR, age standardized DALYs rate.

**Additional file 4:** **Figure S4.** Global exposure and attributable burden of kidney dysfunction by sex. The age-standard SEV (A), ASMR (B) and ASDR (C) of kidney dysfunction by sex from 1990 to 2019.SEV, summary exposure value; ASMR, age standardized mortality rate; DALYs, disability-adjusted life years. ASDR, age standardized DALYs rate.

**Additional file 5:** **Figure S5.** Sex disparity in the global exposure and attributable burden of kidney dysfunction in different age groups. Ratio of male to female SEV (A), ASMR (B) and ASDR (C) of kidney dysfunction, in different age groups in 2019. SEV, summary exposure value; ASMR, age standardized mortality rate; DALYs, disability-adjusted life years. ASDR, age standardized DALYs rate.

**Additional file 6:** **Table 1S.** Global and regional age-standardized morality of kidney dysfunction for both sexes combined in 1990,2000,2010, and 2019, and EAPC of ASMR from 1990 to 2019 and 1990 to 2010.

**Additional file 7**: **Table 2S.** Global and regional age-standardized DALYs of kidney dysfunction for both sexes combined in 1990,2000,2010, and 2019, and EAPC of ASDR from 1990 to 2019 and 1990 to 2010.

**Additional file 8**: **Table 3S.** Age-standardized SEVs of kidney dysfunction for both sexes combined in 1990, 2000,2010 and2019, and EAPC of SEVs from 1990 to 2019 and 1990 to 2010 in 204 countries and territories.

**Additional file 9**: **Table 4S.** Age-standardized morality of kidney dysfunction for both sexes combined in 1990,2000,2010, and 2019, and EAPC of ASMR from 1990 to 2019 and 1990 to 2010 in 204 countries and territories.

**Additional file 10**: **Table 5S.** Age-standardized DALYs of kidney dysfunction for both sexes combined in 1990,2000,2010, and 2019, and EAPC of ASDR from 1990 to 2019 and 1990 to 2010 in 204 countries and territories

## Data Availability

All data generated or analyzed during this study are included in this article and its supplementary material files.

## Abbreviations

SEV: Summary exposure value
ASMR: Age standardized mortality rate
DALYs: Disease adjusted life years
ASDR: Age standardized DALY rate
SDI: Sociodemographic index
EAPC: Estimated annual percentage change
KD: Kidney dysfunction
GFR: Glomerular filtration rate
CKD: Chronic kidney disease
IHD: Ischemic heart diseases
