# Supplementary figures and images for: Global, regional, and national burden of kidney dysfunction from 1990 to 2019: a systematic analysis from the global burden of disease study 2019
Source: BMC Public Health. 2023 Jun 23;23:1218. doi: 10.1186/s12889-023-16130-8 (PMC10288715; doi:10.1186/s12889-023-16130-8)

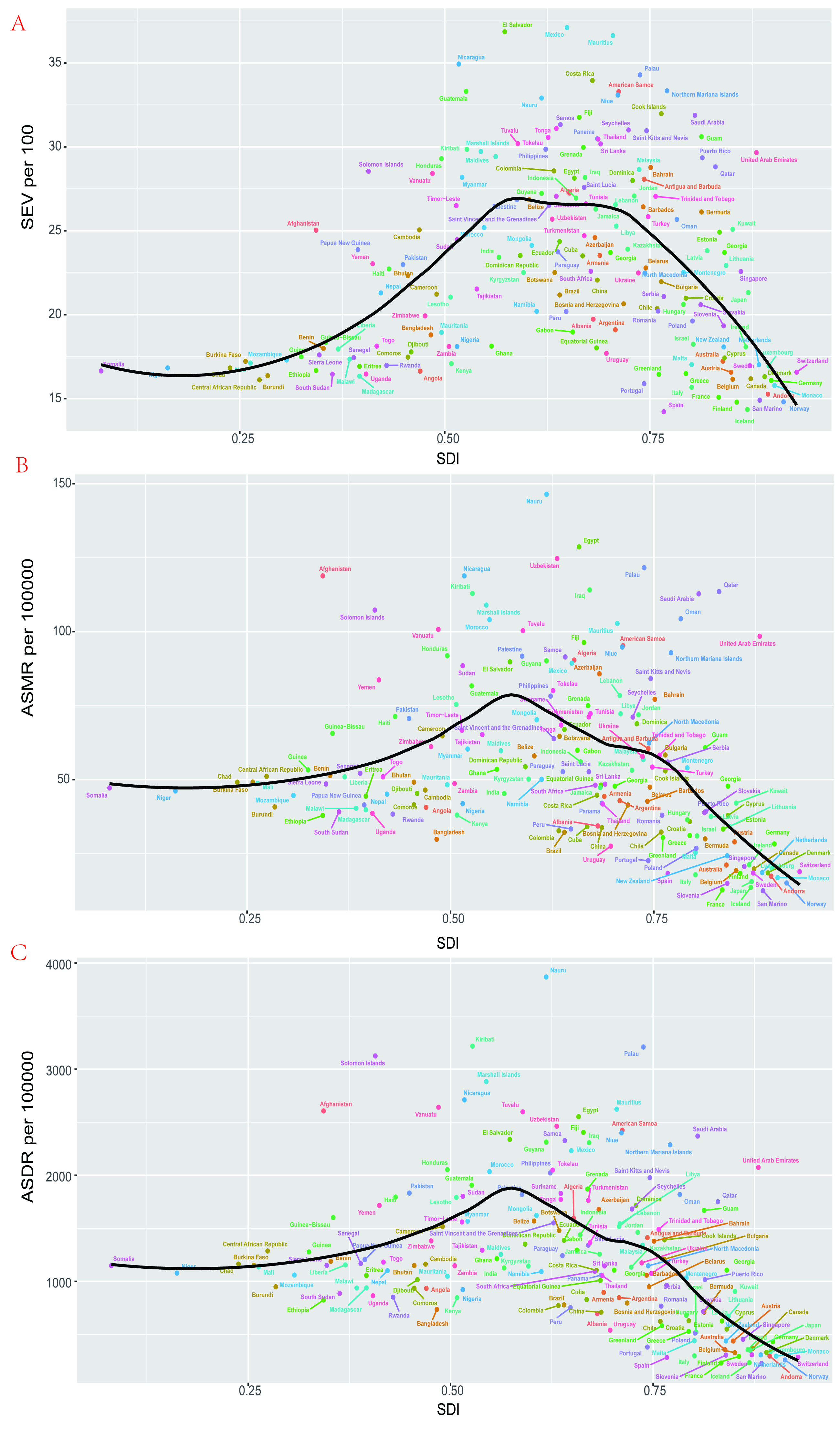

Supplement: Supplementary file 2 — Additional file 2: Figure S2. Correlations of SEV, ASMR as well as ASDR and SDI at the national level. Age-standardized SEV (A), ASMR (B) as well as ASDR (C) for kidney dysfunction and SDI at the regional level in 204 countries and territories from 1990 to 2019. SEV, summary exposure value; ASMR, age standardized mortality rate; DALYs, disability-adjusted life years. ASDR, age standardized DALYs rate; SDI, sociodemographic index. [file 12889_2023_16130_MOESM2_ESM.tif]

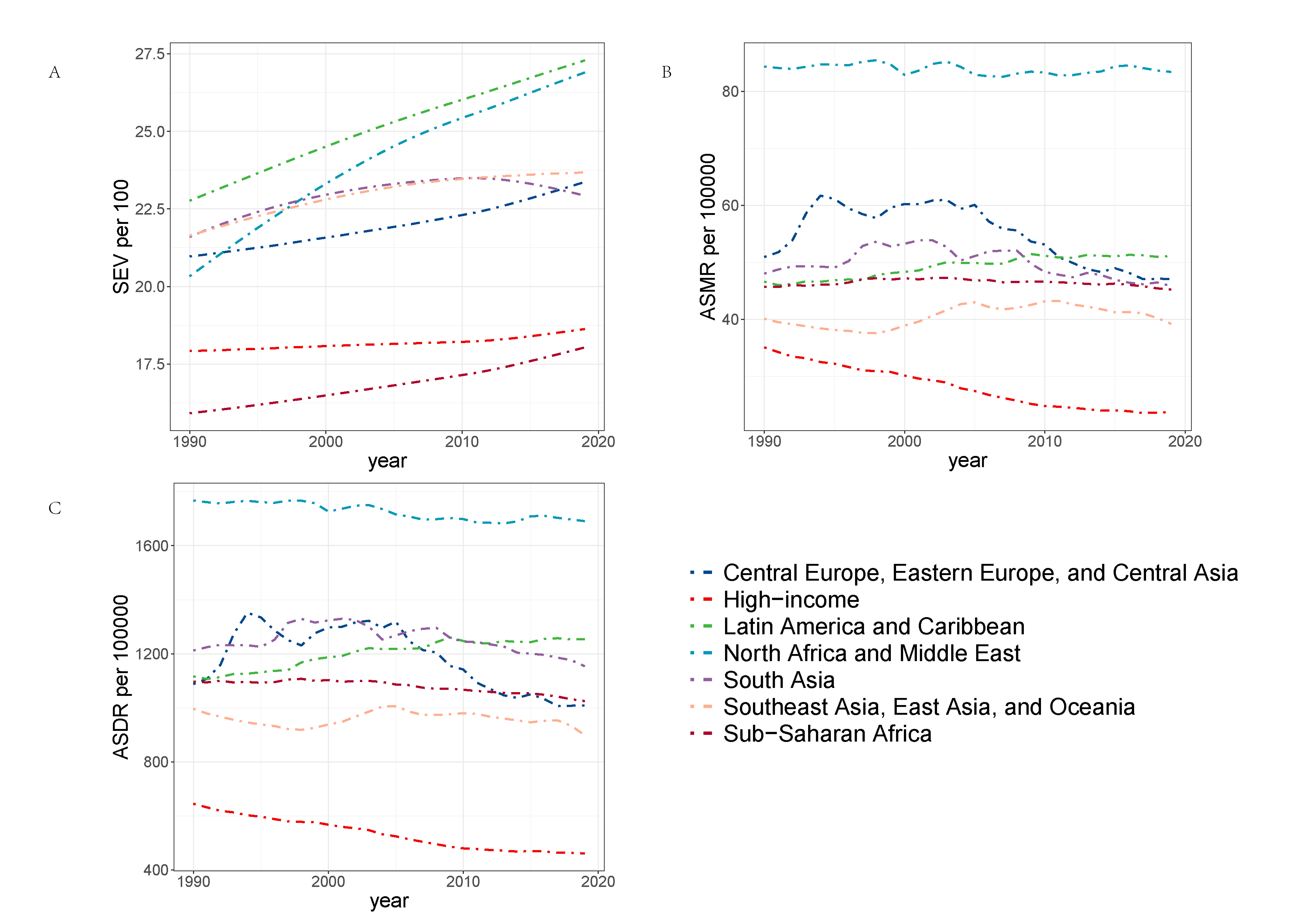

Supplement: Supplementary file 3 — Additional file 3: Figure S3. The exposure and burden of kidney dysfunction in seven GBD super regions. (A) The age standardized SEV, (B) ASMR and (C) ASDR of kidney dysfunction in different SDI regions from 1990 to 2019. Results are showed for both sexes in worldwide. SEV, summary exposure value; ASMR, age standardized mortality rate; DALYs, disability-adjusted life years. ASDR, age standardized DALYs rate. [file 12889_2023_16130_MOESM3_ESM.tif]

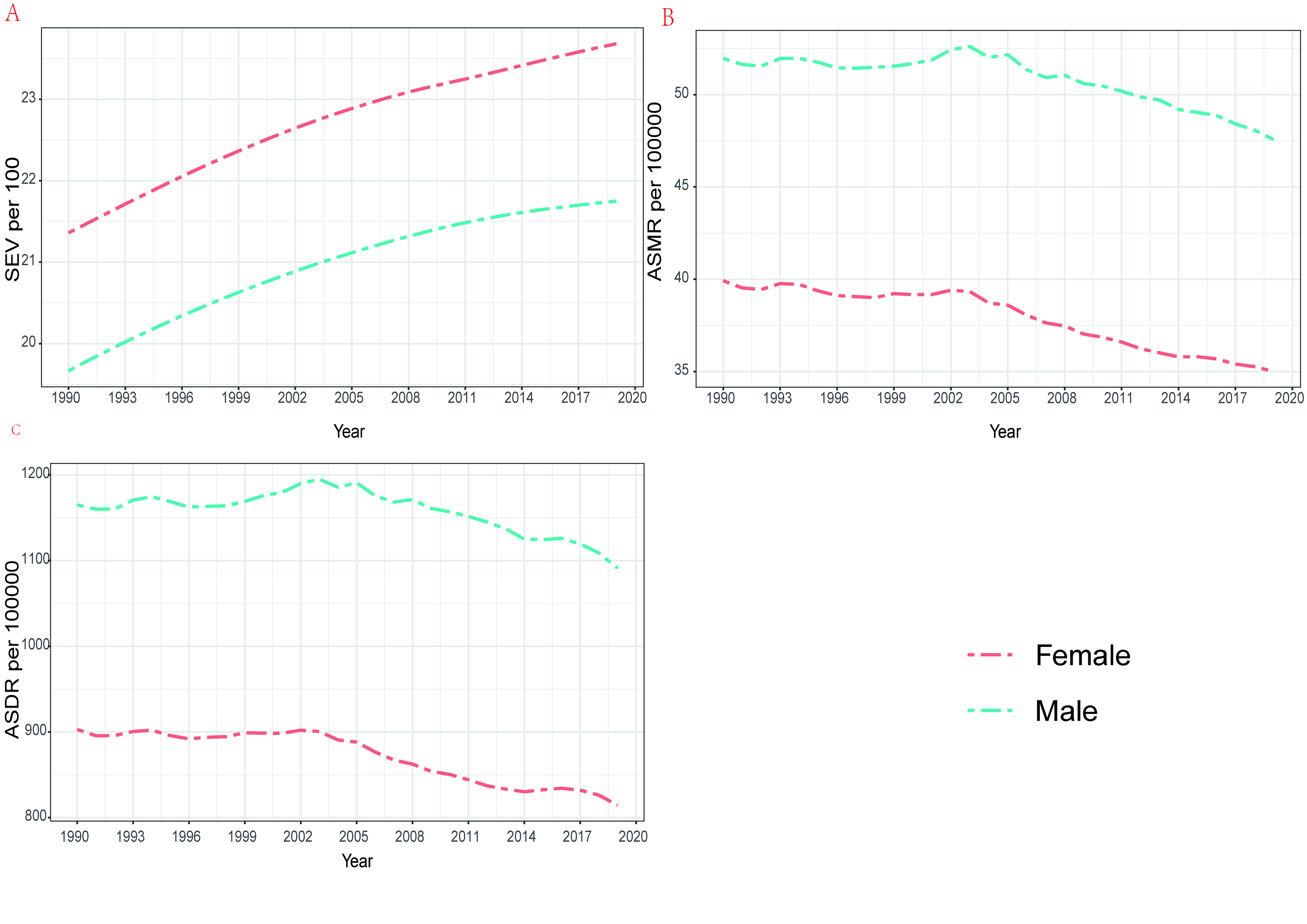

Supplement: Supplementary file 4 — Additional file 4: Figure S4. Global exposure and attributable burden of kidney dysfunction by sex. The age-standard SEV (A), ASMR (B) and ASDR (C) of kidney dysfunction by sex from 1990 to 2019.SEV, summary exposure value; ASMR, age standardized mortality rate; DALYs, disability-adjusted life years. ASDR, age standardized DALYs rate. [file 12889_2023_16130_MOESM4_ESM.tif]

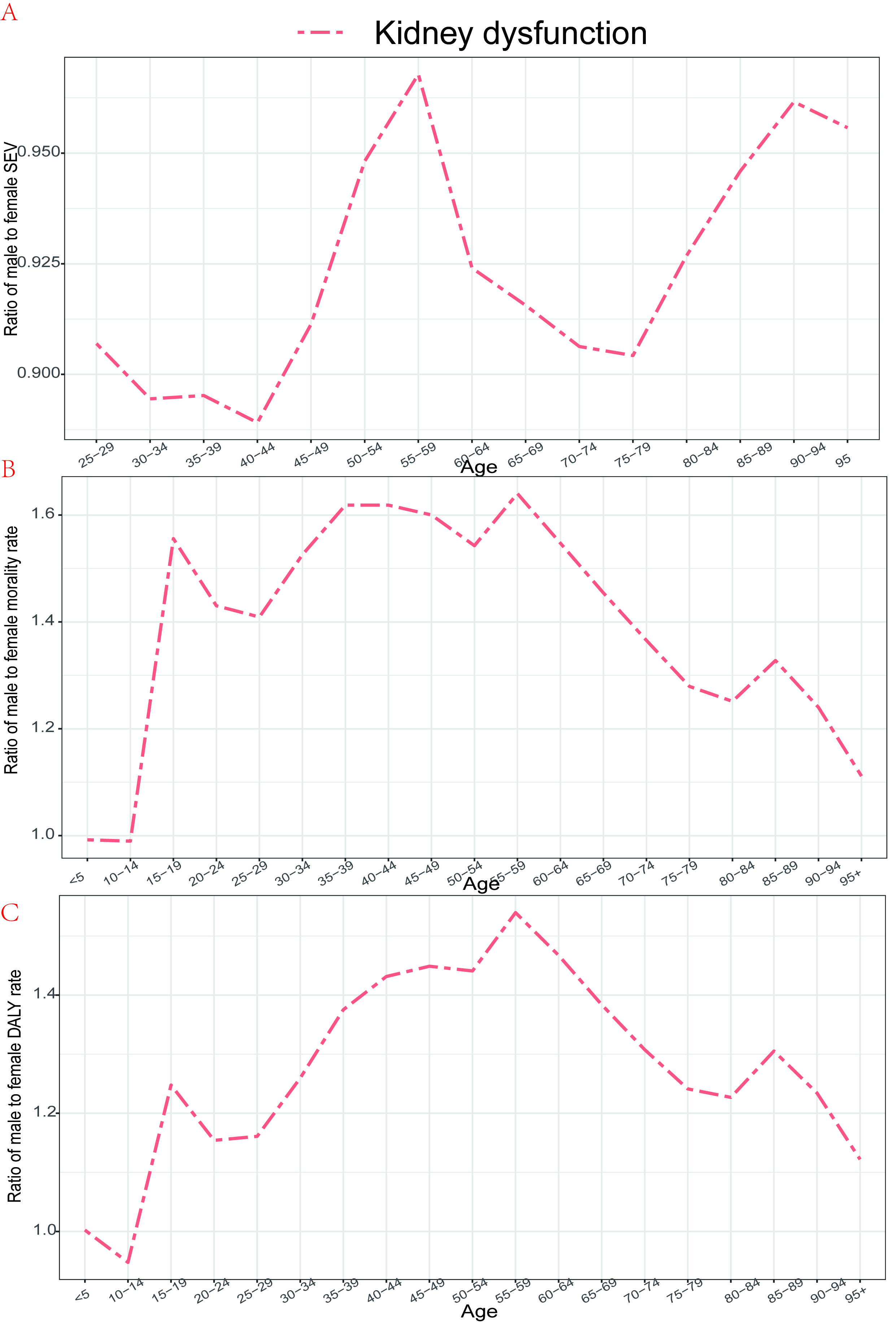

Supplement: Supplementary file 5 — Additional file 5: Figure S5. Sex disparity in the global exposure and attributable burden of kidney dysfunction in different age groups. Ratio of male to female SEV (A), ASMR (B) and ASDR (C) of kidney dysfunction, in different age groups in 2019. SEV, summary exposure value; ASMR, age standardized mortality rate; DALYs, disability-adjusted life years. ASDR, age standardized DALYs rate. [file 12889_2023_16130_MOESM5_ESM.tif]
