# Supplementary material for: Global, regional, and national burden of kidney dysfunction from 1990 to 2019: a systematic analysis from the global burden of disease study 2019
Source: BMC Public Health. 2023 Jun 23;23:1218. doi: 10.1186/s12889-023-16130-8 (PMC10288715; doi:10.1186/s12889-023-16130-8)
Supplement: Supplementary file 6 — Additional file 6: Table 1S. Global and regional age-standardized morality of kidney dysfunction for both sexes combined in 1990,2000,2010, and 2019, and EAPC of ASMR from 1990 to 2019 and 1990 to 2010. [file 12889_2023_16130_MOESM6_ESM.docx]

Table 1S. Global and regional age-standardized morality of kidney dysfunction for both sexes combined in 1990,2000,2010, and 2019, and EAPC of ASMR from 1990 to 2019 and 1990 to 2010

|  | ASMR 1990 | ASMR2000 | ASMR 2010 | ASMR 2019 | EAPC 1990-2010 | EAPC 1990-2019 |
| --- | --- | --- | --- | --- | --- | --- |
| Global  Gender | \| 45.17  (37.62to52.33) \| \| --- \| | 44.72  (37.82to51.43) | 42.94 (36.90to49.27) | \| 40.64(34.81to46.71) \| \| --- \| | -0.18 (-0.26 to -0.11) | -0.35 (-0.41 to -0.29) |
| Male | 51.96(43.66to60.17) | 51.69(43.96to59.46) | 50.47(43.38to57.84) | 47.57(40.82to54.96) | -0.07 (-0.14 to -0.01) | -0.26 (-0.32 to -0.2) |
| Female  SDI | 39.92(32.9to46.79) | 39.17(33.03to45.46) | 36.86(31.28to42.47) | 35.01(29.67to40.79) | -0.33 (-0.41 to -0.25) | -0.47 (-0.53 to -0.42) |
| High SDI | \| 35.91(29.13to43.29) \| \| --- \| | 31.02(25.78to36.5) | 25.67(21.77to29.62) | 24.67(20.97to28.48) | -1.62 (-1.7 to -1.54) | -1.42 (-1.5 to -1.33) |
| High-middle SDI | \| 45.58(36.63to55.18) \| \| --- \| | \| 45.82(36.95to55.03) \| \| --- \| | 41.23(33.78to49.39) | 35.75(29.15to42.45) | -0.48 (-0.68 to -0.29) | -0.96 (-1.12 to -0.8) |
| Middle SDI | \| 48.26(41.47to55.49) \| \| --- \| | 50.05(43.2to56.8) | 53.73(46.59to61.19) | 50.69(43.36to58.78) | 0.65 (0.56 to 0.74) | 0.38 (0.27 to 0.48) |
| Low-middle SDI | \| 46.4(39.66to53.84) \| \| --- \| | 49.58(42.92to56.31) | 48.95(42.36to55.79) | 48.36(41.02to55.76) | 0.4 (0.28 to 0.52) | 0.15 (0.05 to 0.24) |
| Low SDI  Region | \| 46.83(39.9to54.26) \| \| --- \| | 47.44(40.7to54.6) | 46.53(40.48to53.28) | 45.6(39.3to52.46) | 0.05 (-0.01 to 0.1) | -0.1 (-0.15 to -0.05) |
| Andean Latin America | 39.19(34.25to44.38) | 45.07(40.35to50.23) | 50.9(45.02to56.42) | 47.65(39.57to56.76) | 1.35 (1.23 to 1.48) | 0.85 (0.68 to 1.03) |
| Australasia | 37.09(29.62to45.06) | 29.07(23.44to35.25) | 22.7(18.62to26.91) | 21.66(17.91to25.49) | -2.45 (-2.53 to -2.37) | -2.09 (-2.23 to -1.94) |
| Caribbean | 43.89(37.03to51.37) | 41.96(36.13to48.1) | 44.83(38.95to50.95) | 47.48(39.39to55.43) | 0.21 (0.06 to 0.37) | 0.46 (0.35 to 0.56) |
| Central Asia | 49.88(37.1to64.57) | 69.29(52to89.08) | 74.25(57.01to93.99) | 70.2(54.05to88.52) | 1.89 (1.48 to 2.3) | 0.9 (0.57 to 1.23) |
| Central Europe | 48(37.36to59.98) | 46.51(36.56to57.01) | 39.51(31.47to47.9) | 35.58(27.89to44.43) | -1.2 (-1.43 to -0.97) | -1.43 (-1.56 to -1.29) |
| Central Latin America | 51.42(44.97to58.27) | 60.78(54.79to66.79) | 69.98(63.77to76.06) | 71.55(61.61to82.82) | 1.7 (1.61 to 1.79) | 1.24 (1.1 to 1.38) |
| Central Sub-Saharan Africa | 46.37(39.08to54.91) | 45.15(38to53.35) | 42.71(34.32to51.5) | 42.21(32.94to53.32) | -0.42 (-0.47 to -0.38) | -0.41 (-0.44 to -0.38) |
| East Asia | 36.12(30.48to42.39) | 34.15(29.11to39.45) | 39.01(32.37to46.27) | 33.9(27.65to40.99) | 0.72 (0.39 to 1.04) | 0.33 (0.11 to 0.54) |
| Eastern Europe | 52.59(37.42to70.43) | 66.09(47.47to87.41) | 57.26(41.03to75.83) | 49.31(35.44to64.55) | 0.27 (-0.27 to 0.82) | -0.72 (-1.1 to -0.35) |
| Eastern Sub-Saharan Africa | 43.37(37.8to49.2) | 42.62(37.41to48.08) | 40.7(35.91to46.25) | 40.58(35.36to46.94) | -0.36 (-0.41 to -0.32) | -0.31 (-0.34 to -0.28) |
| High-income Asia Pacific | 32.56(27.46to37.69) | 23.74(20.13to27.1) | 18.74(15.41to21.47) | 15.94(12.93to18.45) | -2.85 (-2.97 to -2.72) | -2.56 (-2.67 to -2.45) |
| High-income North America | 40.21(32.46to48.4) | 38.58(31.9to45.47) | 32.81(27.86to37.63) | 33.18(28.47to38.06) | -0.84 (-1.02 to -0.66) | -0.78 (-0.89 to -0.68) |
| North Africa and Middle East | 84.35(71.06to99.1) | 82.86(70.21to96.66) | 83.33(70.88to96.64) | 83.4(69.82to97.32) | -0.09 (-0.16 to -0.03) | -0.05 (-0.09 to -0.01) |
| Oceania | 50.48(41.15to62.03) | 57.56(47.6to68.77) | 57.88(47.23to70.35) | 58.78(47.03to72.88) | 0.67 (0.54 to 0.81) | 0.44 (0.35 to 0.54) |
| South Asia | 48.02(39.6to56.71) | 53.26(44.61to61.91) | 48.3(40.85to56.2) | 45.84(37.34to54.7) | 0.21 (-0.05 to 0.47) | -0.29 (-0.47 to -0.11) |
| Southeast Asia | 53.24(46.08to61.08) | 54.7(47.56to62.31) | 56.88(49.89to64) | 56.55(48.98to64.6) | 0.36 (0.32 to 0.39) | 0.26 (0.23 to 0.3) |
| Southern Latin America | 45.38(38.71to52.26) | 44.22(39.12to49.25) | 40.29(36.02to44.34) | 38.83(34.6to42.95) | -0.3 (-0.54 to -0.06) | -0.5 (-0.63 to -0.37) |
| Southern Sub-Saharan Africa | 37.92(33.44to43.33) | 54.1(48.89to60) | 55.83(50.59to61.72) | 51.01(45.52to56.94) | 2.01 (1.56 to 2.46) | 1.1 (0.76 to 1.44) |
| Tropical Latin America | 44.61(38.82to50.51) | 39.04(34.57to43.48) | 34.55(30.72to38.29) | 32.7(29.18to36.28) | -1.25 (-1.31 to -1.18) | -1.06 (-1.13 to -1) |
| Western Europe | 31.51(25.41to38.03) | 25.86(21.22to30.75) | 21.09(17.6to24.62) | 19.89(16.54to23.16) | -1.95 (-2.01 to -1.89) | -1.68 (-1.78 to -1.59) |
| Western Sub-Saharan Africa | 49.3(42.27to57.58) | 48.36(40.83to56.82) | 48.96(41.43to56.24) | 47.75(40.43to55.65) | 0.01 (-0.04 to 0.07) | -0.04 (-0.08 to -0.01) |

ASMR, age-standard morality rate; EAPC, estimated annual percentage change.
