# Supplementary material for: Global, regional, and national burden of kidney dysfunction from 1990 to 2019: a systematic analysis from the global burden of disease study 2019
Source: BMC Public Health. 2023 Jun 23;23:1218. doi: 10.1186/s12889-023-16130-8 (PMC10288715; doi:10.1186/s12889-023-16130-8)
Supplement: Supplementary file 7 — Additional file 7: Table 2S. Global and regional age-standardized DALYs of kidney dysfunction for both sexes combined in 1990,2000,2010, and 2019, and EAPC of ASDR from 1990 to 2019 and 1990 to 2010. [file 12889_2023_16130_MOESM7_ESM.docx]

Table 2S. Global and regional age-standardized DALYs of kidney dysfunction for both sexes combined in 1990,2000,2010, and 2019, and EAPC of ASDR from 1990 to 2019 and 1990 to 2010

|  | ASDR 1990 | ASDR 2000 | ASDR 2010 | ASDR 2019 | EAPC 1990-2010 | EAPC 1990-2019 |
| --- | --- | --- | --- | --- | --- | --- |
| Global  Gender | \| 1024.13  (898.38to1154.80) \| \| --- \| | 1028.60  (914.76to1154.42) | 995.18 (886.33to1113.72) | \| 945.31(836.33to1066.77) \| \| --- \| | -0.07 (-0.14 to 0) | -0.25 (-0.31 to -0.19) |
| Male | \| 1165.12(1016.44to1320.85) \| \| --- \| | \| 1176.03(1031.59to1322.62) \| \| --- \| | \| 1157.02(1024.76to1297.67) \| \| --- \| | \| 1091.12(956.91to1234.67) \| \| --- \| | 0.04 (-0.03 to 0.11) | -0.17 (-0.24 to -0.1) |
| Female  SDI | 902.88(792.09to1019.04) | 898.56(803.72to1007.66) | 850.34(759.36to947.42) | 814.43(719.3to920.95) | -0.23 (-0.32 to -0.14) | -0.36 (-0.42 to -0.31) |
| High SDI | \| 675.39(575.32to781.28) \| \| --- \| | 601.98(522.02to685.3) | 519.95(461.2to583.7) | 509.55(451.87to571.42) | -1.24 (-1.3 to -1.17) | -1.04 (-1.12 to -0.97) |
| High-middle SDI | \| 973.98(827.67to1131.06) \| \| --- \| | \| 985.7(840.78to1141.89) \| \| --- \| | 871.31(742.93to1006.3) | 753.26(645.97to877.52) | -0.52 (-0.75 to -0.3) | -1.02 (-1.19 to -0.85) |
| Middle SDI | \| 1151.22(1025.17to1287.27) \| \| --- \| | 1171.18(1054.98to1299.8) | 1222.48(1093.55to1359.75) | 1152.78(1018.75to1297.68) | 0.43 (0.35 to 0.51) | 0.2 (0.11 to 0.28) |
| Low-middle SDI | \| 1182.04(1043.92to1338.6) \| \| --- \| | 1245.28(1108.16to1391.75) | 1226.24(1099.67to1367.09) | 1188.9(1039.55to1345.51) | 0.27 (0.16 to 0.38) | 0.06 (-0.02 to 0.14) |
| Low SDI  Region | \| 1160.16(1023.81to1306.87) \| \| --- \| | 1163.88(1024.18to1313.55) | 1133.68(1007.96to1267.7) | 1101.63(964.88to1252.74) | -0.07 (-0.11 to -0.02) | -0.18 (-0.22 to -0.14) |
| Andean Latin America | 938.28(841.05to1043.25) | 1037.65(945.93to1133.93) | 1134.11(1023.71to1245.87) | 1043.73(878.73to1234.78) | 0.98 (0.88 to 1.09) | 0.48 (0.32 to 0.64) |
| Australasia | 615.06(513.48to723.87) | 476.6(406.25to553.14) | 381.46(330.81to434.83) | 367.82(319.74to418.62) | -2.37 (-2.43 to -2.31) | -1.92 (-2.07 to -1.76) |
| Caribbean | 1086.64(955.24to1226.52) | 1073.03(960.38to1197.74) | 1161.1(1029.34to1305.56) | 1233.47(1046.48to1432.94) | 0.47 (0.34 to 0.6) | 0.64 (0.55 to 0.72) |
| Central Asia | 1213(989.65to1470.15) | 1626.44(1320.61to1968.16) | 1670.87(1376.17to1988.45) | 1559.32(1260.36to1876.8) | 1.49 (1.08 to 1.91) | 0.51 (0.18 to 0.85) |
| Central Europe | 987.08(816.07to1171.43) | 915.47(765.2to1077.99) | 763.09(646.2to886.93) | 682.12(562.06to823.17) | -1.48 (-1.68 to -1.29) | -1.63 (-1.74 to -1.51) |
| Central Latin America | 1223.71(1103.57to1358.89) | 1491.55(1376.37to1618.22) | 1726.18(1604.52to1862.55) | 1781.44(1565.02to2028.72) | 1.91 (1.81 to 2.02) | 1.41 (1.26 to 1.56) |
| Central Sub-Saharan Africa | 1121.41(948.3to1317.2) | 1079.73(917.14to1250.51) | 1000.13(830.33to1186.12) | 968.63(784.78to1184.38) | -0.6 (-0.66 to -0.54) | -0.58 (-0.61 to -0.55) |
| East Asia | 871.29(755.89to1001.12) | 790.01(700.34to890.91) | 827.02(716.49to945.53) | 716.94(601.6to839.34) | 0.08 (-0.2 to 0.35) | -0.19 (-0.36 to -0.03) |
| Eastern Europe | 1108.07(857.06to1394.94) | 1427.36(1104.55to1789.2) | 1236.7(955.04to1534.4) | 1048.54(818.23to1308.82) | 0.39 (-0.23 to 1.02) | -0.75 (-1.18 to -0.33) |
| Eastern Sub-Saharan Africa | 1065.14(946.98to1189.24) | 1015.77(903.24to1132.4) | 945.02(836.84to1059.7) | 920.28(804to1058.98) | -0.67 (-0.72 to -0.62) | -0.59 (-0.63 to -0.56) |
| High-income Asia Pacific | 639.12(559.58to721.87) | 496.28(440.53to553.48) | 403.16(354.84to453.2) | 342.75(300.78to387.75) | -2.32 (-2.4 to -2.25) | -2.14 (-2.2 to -2.08) |
| High-income North America | 765.76(645.78to894.11) | 760.32(657.64to870.26) | 676.68(601.49to756.81) | 685.98(612.01to765.36) | -0.41 (-0.59 to -0.24) | -0.42 (-0.52 to -0.33) |
| North Africa and Middle East | 1767.06(1532.18to2015.15) | 1726.61(1504.78to1963.57) | 1698.41(1488.04to1912.2) | 1690.97(1449.36to1945.98) | -0.22 (-0.27 to -0.17) | -0.18 (-0.22 to -0.15) |
| Oceania | 1409.71(1182.31to1696.36) | 1603.36(1358.52to1892.61) | 1604.54(1331.95to1906.7) | 1598.45(1296.13to1955.54) | 0.66 (0.52 to 0.8) | 0.36 (0.26 to 0.47) |
| South Asia | 1212.27(1040.15to1401.83) | 1323.69(1148.92to1495.06) | 1245.06(1094.86to1416.17) | 1154.01(975.73to1346.18) | 0.21 (0.01 to 0.42) | -0.19 (-0.34 to -0.04) |
| Southeast Asia | 1393.49(1240.04to1563.36) | 1401.11(1256.45to1570.38) | 1437.05(1287.75to1593.34) | 1421.48(1257.95to1597.65) | 0.19 (0.15 to 0.23) | 0.13 (0.11 to 0.16) |
| Southern Latin America | 913.01(816.7to1020.46) | 886.6(813.93to964.69) | 804.7(740.83to873.41) | 761.8(697.18to827.56) | -0.38 (-0.58 to -0.18) | -0.62 (-0.73 to -0.5) |
| Southern Sub-Saharan Africa | 956.3(861.13to1064.71) | 1273.9(1168.52to1390.56) | 1287.62(1179.24to1409.17) | 1163.64(1050.14to1293.63) | 1.68 (1.31 to 2.06) | 0.77 (0.46 to 1.09) |
| Tropical Latin America | 1066.43(961.73to1181.31) | 960.71(876.14to1054.52) | 826.9(752.78to909.6) | 788.31(717.56to867.33) | -1.26 (-1.36 to -1.16) | -1.13 (-1.21 to -1.06) |
| Western Europe | 543.95(460.03to634.79) | 442.36(376.43to509.38) | 352.33(305.99to401.67) | 327.11(286.04to372.3) | -2.16 (-2.21 to -2.1) | -1.9 (-2 to -1.8) |
| Western Sub-Saharan Africa | 1152.37(1001.29to1331.57) | 1117.79(948.41to1311.43) | 1111.64(940.38to1281.61) | 1083.85(914.36to1266.47) | -0.14 (-0.19 to -0.08) | -0.15 (-0.18 to -0.12) |

ASDR, age-standard DALYs rate; DALYs: disability-adjusted life years; EAPC: estimated annual percentage change.
