# Supplementary material for: Global, regional, and national burden of kidney dysfunction from 1990 to 2019: a systematic analysis from the global burden of disease study 2019
Source: BMC Public Health. 2023 Jun 23;23:1218. doi: 10.1186/s12889-023-16130-8 (PMC10288715; doi:10.1186/s12889-023-16130-8)
Supplement: Supplementary file 8 — Additional file 8: Table 3S. Age-standardized SEVs of kidney dysfunction for both sexes combined in 1990, 2000,2010 and2019, and EAPC of SEVs from 1990 to 2019 and 1990 to 2010 in 204 countries and territories. [file 12889_2023_16130_MOESM8_ESM.docx]

Table 3S. Age-standardized SEVs of kidney dysfunction for both sexes combined in 1990, 2000,2010 and2019, and EAPC of SEVs from 1990 to 2019 and 1990 to 2010 in 204 countries and territories

| Location | SEVs 2000 | SEVs 2000 | SEVs 2000 | SEVs 2000 | EAPC 1990to2010 | EAPC 1990to2019 |
| --- | --- | --- | --- | --- | --- | --- |
| Afghanistan | 19.76(13.92to26.97) | 21.08(15.24to28.4) | 22.77(16.66to30.23) | 25.04(18.64to32.59) | 0.73 (0.71 to 0.74) | 0.81 (0.78 to 0.83) |
| Albania | 16.82(11.05to23.92) | 18.09(12.16to25.35) | 19.08(13.13to26.48) | 19.74(13.64to27.04) | 0.64 (0.62 to 0.67) | 0.55 (0.52 to 0.58) |
| Algeria | 20.56(14.76to27.73) | 23.05(16.99to30.3) | 25.21(19.01to32.91) | 27.25(20.82to34.9) | 1.05 (1.02 to 1.08) | 0.95 (0.93 to 0.98) |
| American Samoa | 26.62(18.76to35.1) | 29.97(22.08to38.47) | 32.18(24.18to40.63) | 33.29(25.27to41.73) | 0.98 (0.92 to 1.03) | 0.76 (0.7 to 0.82) |
| Andorra | 15.04(10.26to21.56) | 15.33(10.52to21.88) | 15.41(10.62to22) | 15.27(10.44to21.83) | 0.13 (0.11 to 0.14) | 0.06 (0.04 to 0.08) |
| Angola | 14.57(9.02to21.57) | 15.03(9.34to22.04) | 15.72(9.97to22.79) | 16.64(10.71to23.65) | 0.38 (0.37 to 0.4) | 0.46 (0.43 to 0.48) |
| Antigua and Barbuda | 23.12(16.35to31.15) | 25.78(18.8to33.99) | 27.4(20.19to35.7) | 28.07(21.11to36.19) | 0.88 (0.82 to 0.93) | 0.66 (0.6 to 0.72) |
| Argentina | 17.22(11.8to24.04) | 18.4(12.95to25.2) | 19(13.53to25.93) | 19.1(13.65to26.1) | 0.51 (0.47 to 0.55) | 0.35 (0.31 to 0.4) |
| Armenia | 19.86(13.27to27.62) | 20.7(13.88to28.52) | 21.92(14.93to29.9) | 23.53(16.35to31.62) | 0.5 (0.48 to 0.52) | 0.58 (0.56 to 0.61) |
| Australia | 16.33(11.58to22.74) | 16.55(11.74to23.11) | 16.82(11.97to23.46) | 17.24(12.3to23.81) | 0.15 (0.14 to 0.15) | 0.18 (0.17 to 0.19) |
| Austria | 15.14(10.29to21.48) | 15.83(10.86to22.28) | 16.3(11.27to22.85) | 16.58(11.55to23.1) | 0.38 (0.36 to 0.4) | 0.31 (0.29 to 0.33) |
| Azerbaijan | 20.47(13.69to28.43) | 21.3(14.41to29.27) | 22.66(15.6to30.84) | 24.6(17.43to32.63) | 0.51 (0.49 to 0.54) | 0.63 (0.59 to 0.67) |
| Bahamas | 22.43(15.63to30.39) | 24.86(18.01to32.73) | 26.27(19.24to34.39) | 26.78(19.57to34.74) | 0.82 (0.76 to 0.88) | 0.6 (0.54 to 0.66) |
| Bahrain | 21.53(15.71to28.73) | 25.1(18.97to32.25) | 27.35(21.08to34.64) | 28.77(22.29to36.12) | 1.24 (1.16 to 1.32) | 0.96 (0.89 to 1.04) |
| Bangladesh | 16.17(10.32to23.3) | 17.58(11.47to24.85) | 18.41(12.14to25.71) | 18.8(12.64to26.31) | 0.67 (0.63 to 0.72) | 0.51 (0.46 to 0.55) |
| Barbados | 21.96(15.32to29.8) | 24.32(17.53to32.35) | 25.78(18.7to33.99) | 26.43(19.39to34.49) | 0.83 (0.77 to 0.88) | 0.63 (0.57 to 0.68) |
| Belarus | 20.95(14.1to28.75) | 21.07(14.13to29.13) | 21.4(14.46to29.57) | 22.79(15.73to30.86) | 0.11 (0.09 to 0.12) | 0.24 (0.2 to 0.29) |
| Belgium | 15.69(10.78to22.22) | 16.2(11.23to22.77) | 16.35(11.36to22.95) | 16.16(11.24to22.78) | 0.21 (0.19 to 0.24) | 0.1 (0.07 to 0.13) |
| Belize | 21.09(14.51to28.78) | 23.73(16.82to31.58) | 25.68(18.81to33.66) | 26.85(19.67to34.92) | 1 (0.96 to 1.05) | 0.83 (0.77 to 0.88) |
| Benin | 15.67(10.1to22.6) | 16.5(10.82to23.5) | 17.3(11.57to24.36) | 18(12.17to25.05) | 0.5 (0.49 to 0.5) | 0.48 (0.47 to 0.48) |
| Bermuda | 21.03(14.58to28.71) | 23.61(16.92to31.37) | 25.29(18.49to33.28) | 26.12(19.14to34.01) | 0.95 (0.89 to 1) | 0.74 (0.68 to 0.8) |
| Bhutan | 18.58(12.32to26.17) | 19.83(13.42to27.47) | 21.12(14.46to28.91) | 22.33(15.6to30.26) | 0.65 (0.64 to 0.65) | 0.63 (0.63 to 0.64) |
| Bolivia (Plurinational State of) | 17.58(11.79to24.73) | 18.92(13.12to26.15) | 20.13(14.06to27.54) | 21.17(14.92to28.66) | 0.69 (0.67 to 0.7) | 0.64 (0.62 to 0.65) |
| Bosnia and Herzegovina | 17.03(11.22to24.25) | 18.67(12.65to25.99) | 19.88(13.74to27.22) | 20.65(14.45to28.16) | 0.79 (0.75 to 0.82) | 0.66 (0.62 to 0.69) |
| Botswana | 18.21(12.1to25.59) | 19.18(12.93to26.73) | 20.61(14.19to28.29) | 22.52(15.87to30.23) | 0.62 (0.6 to 0.65) | 0.73 (0.7 to 0.76) |
| Brazil | 18.9(12.99to26.17) | 19.49(13.59to26.83) | 20.21(14.3to27.57) | 21.18(15.13to28.59) | 0.34 (0.33 to 0.34) | 0.38 (0.37 to 0.4) |
| Brunei Darussalam | 22.93(16.73to30.34) | 23.8(17.56to31.14) | 24(17.74to31.43) | 23.43(17.2to30.73) | 0.23 (0.2 to 0.27) | 0.08 (0.04 to 0.13) |
| Bulgaria | 18.18(12.19to25.41) | 19.2(13.03to26.6) | 20.48(14.14to28.03) | 21.97(15.57to29.73) | 0.6 (0.59 to 0.61) | 0.65 (0.64 to 0.67) |
| Burkina Faso | 15.05(9.62to22.05) | 15.91(10.34to22.97) | 16.65(10.98to23.68) | 17.23(11.58to24.4) | 0.51 (0.5 to 0.52) | 0.47 (0.45 to 0.48) |
| Burundi | 15.25(9.55to22.15) | 15.69(9.97to22.62) | 16.07(10.31to23.15) | 16.37(10.45to23.47) | 0.26 (0.26 to 0.27) | 0.24 (0.24 to 0.25) |
| Cabo Verde | 15.42(9.9to22.49) | 16.6(10.88to23.74) | 17.77(11.94to25.08) | 18.82(12.8to26.24) | 0.72 (0.71 to 0.72) | 0.69 (0.68 to 0.7) |
| Cambodia | 21.84(14.8to29.93) | 22.6(15.42to30.76) | 23.65(16.4to31.9) | 25.06(17.56to33.38) | 0.4 (0.39 to 0.42) | 0.47 (0.45 to 0.49) |
| Cameroon | 17.39(11.64to24.43) | 19.03(13.09to26.25) | 20.31(14.2to27.97) | 21.23(15.04to28.94) | 0.79 (0.76 to 0.82) | 0.68 (0.65 to 0.71) |
| Canada | 16.04(11.11to22.62) | 16.33(11.4to22.93) | 16.41(11.36to23.06) | 16.18(11.09to22.78) | 0.12 (0.1 to 0.13) | 0.04 (0.01 to 0.06) |
| Central African Republic | 14.79(9.16to21.8) | 15.13(9.38to22.15) | 15.57(9.74to22.65) | 16.12(10.23to23.14) | 0.26 (0.25 to 0.26) | 0.29 (0.28 to 0.3) |
| Chad | 15.14(9.72to22.06) | 15.56(9.97to22.52) | 16.12(10.46to23.14) | 16.83(11.14to23.85) | 0.31 (0.3 to 0.32) | 0.36 (0.35 to 0.38) |
| Chile | 17.23(11.78to23.97) | 18.84(13.32to25.63) | 19.87(14.24to26.87) | 20.37(14.8to27.46) | 0.73 (0.69 to 0.78) | 0.57 (0.52 to 0.61) |
| China | 20.77(14.11to28.78) | 21.9(15.09to29.9) | 22.31(15.44to30.32) | 22.07(15.28to30.03) | 0.38 (0.34 to 0.42) | 0.21 (0.16 to 0.26) |
| Colombia | 24.18(17.08to32.26) | 25.82(18.55to34.09) | 27.32(20.08to35.59) | 28.57(21.36to36.87) | 0.62 (0.61 to 0.63) | 0.57 (0.56 to 0.59) |
| Comoros | 15.76(9.96to22.82) | 16.31(10.45to23.54) | 16.89(10.89to24.18) | 17.47(11.51to24.65) | 0.35 (0.35 to 0.35) | 0.36 (0.35 to 0.36) |
| Congo | 15.78(9.92to22.84) | 16.29(10.32to23.4) | 16.9(10.87to24.08) | 17.6(11.6to24.84) | 0.35 (0.34 to 0.35) | 0.37 (0.37 to 0.38) |
| Cook Islands | 26.22(18.63to34.49) | 28.49(20.71to36.79) | 30.51(22.59to39.04) | 31.98(23.98to40.51) | 0.77 (0.75 to 0.79) | 0.69 (0.66 to 0.71) |
| Costa Rica | 32(24.73to40.05) | 32.72(25.54to40.95) | 33.28(26.12to41.45) | 33.95(26.42to42.21) | 0.2 (0.19 to 0.21) | 0.19 (0.19 to 0.2) |
| Croatia | 18.21(12.23to25.19) | 19.45(13.37to26.48) | 20.39(14.21to27.54) | 20.99(14.92to28.42) | 0.57 (0.55 to 0.6) | 0.49 (0.46 to 0.51) |
| Cuba | 19.15(12.81to26.84) | 20.75(14.19to28.39) | 22.24(15.54to30.07) | 23.51(16.76to31.31) | 0.75 (0.74 to 0.77) | 0.71 (0.69 to 0.72) |
| Cyprus | 16.96(11.89to23.48) | 17.7(12.55to24.28) | 17.9(12.76to24.57) | 17.43(12.23to24.04) | 0.28 (0.24 to 0.32) | 0.1 (0.05 to 0.16) |
| Czechia | 17.91(11.95to25.02) | 18.92(12.84to26.16) | 19.64(13.44to26.76) | 20.07(13.93to27.35) | 0.47 (0.45 to 0.49) | 0.39 (0.36 to 0.41) |
| C么te d'Ivoire | 16.64(11.02to23.7) | 17.54(11.78to24.56) | 18.26(12.39to25.38) | 18.76(12.8to26.1) | 0.47 (0.46 to 0.49) | 0.41 (0.39 to 0.43) |
| Democratic People's Republic of Korea | 22.49(15.65to30.63) | 22.99(15.84to31.17) | 23.42(16.19to31.49) | 23.88(16.69to31.97) | 0.21 (0.2 to 0.21) | 0.2 (0.2 to 0.2) |
| Democratic Republic of the Congo | 15.05(9.32to22.16) | 15.18(9.47to22.31) | 15.47(9.78to22.65) | 16.4(10.58to23.39) | 0.14 (0.12 to 0.15) | 0.26 (0.22 to 0.3) |
| Denmark | 15.24(10.27to21.73) | 15.96(10.91to22.44) | 16.3(11.2to22.81) | 16.31(11.26to22.86) | 0.35 (0.32 to 0.38) | 0.23 (0.2 to 0.26) |
| Djibouti | 15.34(9.65to22.35) | 15.89(10.15to22.84) | 16.71(10.8to23.92) | 17.79(11.64to25.15) | 0.43 (0.41 to 0.45) | 0.51 (0.48 to 0.53) |
| Dominica | 23.98(16.96to31.99) | 26.38(19.24to34.49) | 27.65(20.42to35.79) | 28(20.72to36.19) | 0.74 (0.68 to 0.8) | 0.52 (0.46 to 0.58) |
| Dominican Republic | 18.32(12.15to25.77) | 20.31(13.77to27.98) | 22.1(15.25to30.04) | 23.53(16.65to31.53) | 0.95 (0.92 to 0.97) | 0.86 (0.84 to 0.89) |
| Ecuador | 17.75(11.85to24.73) | 19.98(13.88to27.22) | 22.26(15.83to29.77) | 24.36(17.82to32.18) | 1.14 (1.13 to 1.16) | 1.09 (1.08 to 1.11) |
| Egypt | 20.47(14.7to27.64) | 23.48(17.51to30.61) | 25.94(19.69to33.4) | 28.14(21.61to35.85) | 1.22 (1.17 to 1.26) | 1.07 (1.03 to 1.11) |
| El Salvador | 26.34(18.84to34.55) | 33.05(25.47to41.11) | 36.31(28.75to44.41) | 36.86(28.94to45.01) | 1.67 (1.51 to 1.83) | 1.1 (0.94 to 1.26) |
| Equatorial Guinea | 14.68(9.02to21.73) | 16(10.15to23.22) | 17.14(11.14to24.44) | 18.02(11.94to25.2) | 0.79 (0.77 to 0.81) | 0.71 (0.68 to 0.73) |
| Eritrea | 14.88(9.24to21.86) | 15.3(9.66to22.23) | 15.98(10.2to22.95) | 16.92(11.03to24.14) | 0.36 (0.34 to 0.38) | 0.44 (0.42 to 0.47) |
| Estonia | 21.87(14.88to29.72) | 22.63(15.59to30.62) | 23.64(16.39to31.67) | 24.92(17.54to33.06) | 0.39 (0.38 to 0.4) | 0.45 (0.43 to 0.46) |
| Eswatini | 19.6(13.29to27.14) | 20.07(13.57to27.73) | 21.06(14.46to28.8) | 23.1(16.08to30.92) | 0.36 (0.33 to 0.39) | 0.54 (0.48 to 0.59) |
| Ethiopia | 15.01(9.33to21.95) | 15.3(9.57to22.25) | 15.84(10.1to22.84) | 16.68(10.86to23.76) | 0.27 (0.25 to 0.29) | 0.36 (0.33 to 0.38) |
| Fiji | 28.02(20.07to36.38) | 29.34(21.45to37.75) | 30.61(22.71to39.1) | 31.76(23.64to40.37) | 0.44 (0.44 to 0.45) | 0.43 (0.43 to 0.43) |
| Finland | 14.05(9.08to20.64) | 14.41(9.51to20.91) | 14.63(9.74to21.03) | 14.79(9.94to21.21) | 0.21 (0.2 to 0.22) | 0.17 (0.16 to 0.18) |
| France | 14.22(9.47to20.75) | 11.84(9.11to15.55) | 11.58(9.03to15) | 15.09(10.32to21.65) | -1.01 (-1.21 to -0.81) | 0.05 (-0.28 to 0.39) |
| Gabon | 16.13(10.26to23.32) | 16.96(11.05to24.16) | 17.93(11.9to25.25) | 18.97(12.75to26.54) | 0.53 (0.53 to 0.54) | 0.56 (0.55 to 0.57) |
| Gambia | 15.57(10.1to22.58) | 16.26(10.65to23.28) | 17.06(11.36to24.2) | 17.9(12.17to25.01) | 0.46 (0.46 to 0.47) | 0.48 (0.48 to 0.49) |
| Georgia | 20.42(13.62to28.21) | 20.98(14.09to29.2) | 22.03(15.02to30.14) | 23.71(16.57to31.81) | 0.38 (0.36 to 0.41) | 0.51 (0.47 to 0.54) |
| Germany | 15.52(10.69to22.07) | 15.43(10.73to21.8) | 15.51(10.85to21.87) | 16.09(11.3to22.66) | 0 (-0.02 to 0.01) | 0.1 (0.07 to 0.13) |
| Ghana | 15.37(9.7to22.33) | 16.38(10.61to23.42) | 17.34(11.58to24.49) | 18.13(12.25to25.15) | 0.61 (0.6 to 0.62) | 0.57 (0.56 to 0.58) |
| Greece | 16.82(11.75to23.41) | 17.09(12.03to23.6) | 17.11(12.08to23.6) | 16.5(11.52to23.1) | 0.08 (0.06 to 0.1) | -0.04 (-0.08 to 0) |
| Greenland | 15.66(10.6to22.42) | 15.83(10.79to22.57) | 16.08(10.99to22.74) | 16.45(11.31to23.21) | 0.13 (0.12 to 0.14) | 0.16 (0.15 to 0.17) |
| Grenada | 23.17(16.18to30.87) | 26.23(19.12to34.09) | 28.54(21.39to36.66) | 29.97(22.76to38.14) | 1.06 (1.01 to 1.11) | 0.88 (0.83 to 0.93) |
| Guam | 25.77(18.24to33.93) | 27.88(20.08to36.2) | 29.51(21.64to37.94) | 30.61(22.51to39.09) | 0.7 (0.67 to 0.72) | 0.59 (0.56 to 0.62) |
| Guatemala | 25.19(17.85to33.22) | 28.55(21.03to36.7) | 31.32(23.68to39.53) | 33.31(25.67to41.56) | 1.11 (1.07 to 1.15) | 0.96 (0.92 to 1) |
| Guinea | 15.38(9.86to22.38) | 16.16(10.57to23.23) | 16.87(11.18to23.86) | 17.49(11.75to24.5) | 0.47 (0.46 to 0.48) | 0.44 (0.43 to 0.45) |
| Guinea-Bissau | 16.09(10.43to23.17) | 16.99(11.21to24.03) | 17.76(11.92to24.9) | 18.35(12.42to25.66) | 0.5 (0.49 to 0.51) | 0.45 (0.44 to 0.46) |
| Guyana | 21.36(14.79to29.04) | 23.37(16.48to31.16) | 25.39(18.45to33.27) | 27.23(20.08to35.66) | 0.87 (0.86 to 0.88) | 0.84 (0.83 to 0.85) |
| Haiti | 19.42(12.98to26.93) | 21.08(14.32to28.77) | 22.16(15.25to29.91) | 22.72(15.85to30.69) | 0.68 (0.64 to 0.72) | 0.53 (0.49 to 0.57) |
| Honduras | 24.47(17.12to32.44) | 26.2(18.88to34.14) | 27.86(20.66to36) | 29.3(21.78to37.4) | 0.66 (0.65 to 0.66) | 0.62 (0.61 to 0.63) |
| Hungary | 17.24(11.26to24.33) | 18.46(12.44to25.64) | 19.62(13.51to26.88) | 20.62(14.38to28.03) | 0.65 (0.64 to 0.66) | 0.62 (0.61 to 0.63) |
| Iceland | 13.96(9.29to20.14) | 14.3(9.59to20.71) | 14.43(9.7to20.89) | 14.36(9.61to20.78) | 0.17 (0.15 to 0.19) | 0.1 (0.08 to 0.12) |
| India | 22.49(15.45to30.56) | 23.86(16.65to31.9) | 24.28(17.08to32.35) | 23.42(16.32to31.57) | 0.39 (0.34 to 0.44) | 0.16 (0.09 to 0.23) |
| Indonesia | 23.61(16.35to31.98) | 24.5(17.12to32.85) | 25.63(18.14to34.02) | 26.95(19.21to35.51) | 0.41 (0.4 to 0.42) | 0.46 (0.44 to 0.47) |
| Iran (Islamic Republic of) | 22.81(16.72to30.01) | 24.15(18.01to31.3) | 25.25(19.08to32.45) | 26.24(19.93to33.74) | 0.52 (0.5 to 0.53) | 0.47 (0.46 to 0.48) |
| Iraq | 20.44(14.73to27.57) | 22.24(16.2to29.49) | 24.86(18.39to32.53) | 28.18(21.51to35.85) | 0.99 (0.96 to 1.03) | 1.12 (1.08 to 1.15) |
| Ireland | 18.21(12.67to25.31) | 17.65(12.4to24.36) | 17.56(12.46to24.17) | 18.09(12.83to24.83) | -0.19 (-0.22 to -0.16) | -0.04 (-0.08 to 0.01) |
| Israel | 17.23(12.03to23.8) | 18.27(13.05to25.04) | 18.6(13.31to25.47) | 18.24(13.04to24.99) | 0.4 (0.35 to 0.45) | 0.2 (0.14 to 0.26) |
| Italy | 15.93(10.84to22.53) | 15.72(10.7to22.36) | 15.64(10.63to22.24) | 15.68(10.65to22.26) | -0.09 (-0.1 to -0.08) | -0.06 (-0.07 to -0.04) |
| Jamaica | 21.83(15.16to29.5) | 24.87(17.92to32.56) | 26.21(19.18to34.11) | 26.29(19.07to34.17) | 0.96 (0.87 to 1.06) | 0.61 (0.51 to 0.71) |
| Japan | 21.09(15.33to28.49) | 20.93(15.14to28.29) | 20.94(15.07to28.28) | 21.32(15.47to28.55) | -0.04 (-0.05 to -0.03) | 0.03 (0.01 to 0.04) |
| Jordan | 18.84(13.26to25.74) | 21.26(15.37to28.41) | 24.08(17.94to31.42) | 27.08(20.74to34.75) | 1.24 (1.24 to 1.25) | 1.26 (1.25 to 1.26) |
| Kazakhstan | 20.72(13.86to28.67) | 21.21(14.26to29.22) | 22.19(15.08to30.31) | 23.91(16.68to31.96) | 0.34 (0.32 to 0.37) | 0.48 (0.44 to 0.52) |
| Kenya | 15.4(9.69to22.36) | 15.74(9.94to22.7) | 16.27(10.44to23.32) | 17.09(11.16to24.3) | 0.28 (0.26 to 0.29) | 0.35 (0.33 to 0.37) |
| Kiribati | 26.16(18.37to34.58) | 27.52(19.58to35.97) | 28.76(20.73to37.2) | 29.85(21.78to38.45) | 0.48 (0.47 to 0.49) | 0.45 (0.45 to 0.46) |
| Kuwait | 19.67(14.27to26.81) | 21.2(15.57to28.26) | 23.05(17.11to30.13) | 25.09(18.92to32.45) | 0.8 (0.79 to 0.81) | 0.84 (0.83 to 0.86) |
| Kyrgyzstan | 20.66(13.8to28.64) | 20.9(14.09to28.82) | 21.42(14.66to29.37) | 22.52(15.57to30.71) | 0.18 (0.16 to 0.2) | 0.28 (0.25 to 0.31) |
| Lao People's Democratic Republic | 24.25(16.9to32.58) | 25.37(17.78to33.82) | 26.7(18.9to35.28) | 28.13(20.35to36.7) | 0.48 (0.48 to 0.49) | 0.51 (0.5 to 0.52) |
| Latvia | 21.12(14.25to29.12) | 21.55(14.66to29.57) | 22.36(15.32to30.43) | 23.81(16.62to31.93) | 0.29 (0.27 to 0.31) | 0.4 (0.36 to 0.43) |
| Lebanon | 18.33(12.81to25.22) | 21.03(15.29to28.08) | 23.84(17.91to31.16) | 26.55(20.31to33.95) | 1.33 (1.32 to 1.34) | 1.28 (1.27 to 1.29) |
| Lesotho | 17.72(11.76to24.9) | 18.16(12.06to25.47) | 19.1(12.75to26.75) | 21.05(14.48to28.8) | 0.37 (0.34 to 0.41) | 0.56 (0.5 to 0.62) |
| Liberia | 15.6(10to22.67) | 16.69(10.99to23.73) | 17.49(11.72to24.62) | 17.96(12.09to25.04) | 0.58 (0.56 to 0.61) | 0.48 (0.45 to 0.51) |
| Libya | 18.71(13.25to25.57) | 21.36(15.53to28.4) | 23.61(17.4to30.78) | 25.28(19.15to32.53) | 1.18 (1.14 to 1.22) | 1.03 (0.99 to 1.08) |
| Lithuania | 21.49(14.62to29.31) | 21.7(14.75to29.73) | 22.05(15.02to30.18) | 22.93(15.9to31.13) | 0.13 (0.12 to 0.13) | 0.2 (0.18 to 0.22) |
| Luxembourg | 16.34(11.22to22.86) | 17.39(12.2to23.92) | 17.61(12.41to24.26) | 16.95(11.8to23.61) | 0.38 (0.32 to 0.44) | 0.13 (0.06 to 0.21) |
| Madagascar | 14.9(9.23to21.92) | 15.25(9.52to22.19) | 15.73(9.98to22.7) | 16.34(10.44to23.48) | 0.27 (0.26 to 0.28) | 0.32 (0.3 to 0.33) |
| Malawi | 15.56(9.81to22.54) | 16.13(10.34to23.09) | 16.74(10.79to23.9) | 17.36(11.35to24.59) | 0.37 (0.37 to 0.37) | 0.38 (0.37 to 0.38) |
| Malaysia | 23.81(16.52to31.93) | 26.47(19.05to34.78) | 28.02(20.64to36.53) | 28.65(21.01to37.17) | 0.85 (0.79 to 0.9) | 0.62 (0.56 to 0.68) |
| Maldives | 24.93(17.45to33.34) | 27.36(19.75to35.65) | 28.9(21.21to37.07) | 29.42(21.67to37.78) | 0.77 (0.72 to 0.81) | 0.57 (0.51 to 0.62) |
| Mali | 15.07(9.62to22.07) | 15.8(10.24to22.85) | 16.5(10.82to23.52) | 17.12(11.37to24.08) | 0.45 (0.45 to 0.46) | 0.44 (0.43 to 0.44) |
| Malta | 16.48(11.41to23.18) | 17.04(11.98to23.76) | 17.23(12.18to23.91) | 17.03(12.03to23.69) | 0.23 (0.2 to 0.26) | 0.12 (0.08 to 0.15) |
| Marshall Islands | 25.62(17.92to33.91) | 26.89(19.11to35.19) | 28.29(20.36to36.6) | 29.73(21.8to38.11) | 0.5 (0.5 to 0.5) | 0.51 (0.51 to 0.52) |
| Mauritania | 16.55(10.82to23.67) | 17.29(11.51to24.4) | 18.11(12.29to25.2) | 18.95(13.02to26.12) | 0.45 (0.45 to 0.45) | 0.47 (0.46 to 0.47) |
| Mauritius | 29.03(21.19to37.33) | 32.22(24.33to40.71) | 34.81(26.55to43.44) | 36.63(28.42to45.4) | 0.92 (0.89 to 0.95) | 0.8 (0.76 to 0.83) |
| Mexico | 29.76(21.94to37.85) | 32.85(25.1to41.04) | 35.36(27.71to43.6) | 37.11(29.38to45.39) | 0.88 (0.85 to 0.91) | 0.76 (0.73 to 0.79) |
| Micronesia (Federated States of) | 26.92(19.03to35.36) | 29.37(21.4to37.73) | 31.37(23.33to39.63) | 32.87(24.83to41.26) | 0.78 (0.75 to 0.8) | 0.68 (0.66 to 0.71) |
| Monaco | 15.11(10.22to21.72) | 15.48(10.54to22.05) | 15.7(10.77to22.32) | 15.79(10.81to22.4) | 0.2 (0.18 to 0.21) | 0.15 (0.13 to 0.16) |
| Mongolia | 21.62(14.64to29.48) | 22.63(15.54to30.74) | 23.47(16.27to31.59) | 24.13(16.95to32.56) | 0.42 (0.4 to 0.43) | 0.38 (0.36 to 0.39) |
| Montenegro | 19.8(13.65to27.42) | 20.91(14.66to28.52) | 21.84(15.41to29.51) | 22.52(16.02to30.14) | 0.5 (0.49 to 0.51) | 0.44 (0.43 to 0.46) |
| Morocco | 16.38(11.2to23.17) | 18.62(13.03to25.49) | 21.6(15.67to28.86) | 25.19(18.92to32.7) | 1.41 (1.38 to 1.44) | 1.5 (1.47 to 1.53) |
| Mozambique | 15.08(9.37to22.03) | 15.63(9.8to22.57) | 16.39(10.45to23.61) | 17.31(11.28to24.6) | 0.42 (0.41 to 0.43) | 0.48 (0.46 to 0.49) |
| Myanmar | 24.09(16.69to32.44) | 25.11(17.56to33.51) | 26.51(18.81to35.11) | 28.19(20.26to36.72) | 0.48 (0.47 to 0.5) | 0.54 (0.52 to 0.56) |
| Namibia | 17.58(11.67to24.81) | 17.93(11.88to25.29) | 18.73(12.55to26.02) | 20.2(13.83to27.62) | 0.32 (0.29 to 0.35) | 0.46 (0.42 to 0.51) |
| Nauru | 27.9(19.93to36.3) | 28.89(20.96to37.31) | 30.57(22.62to39.02) | 32.91(24.92to41.42) | 0.46 (0.43 to 0.48) | 0.57 (0.53 to 0.6) |
| Nepal | 16.65(10.83to23.69) | 17.3(11.54to24.38) | 18.52(12.81to25.73) | 21.3(14.94to28.98) | 0.53 (0.5 to 0.57) | 0.79 (0.71 to 0.88) |
| Netherlands | 16.15(10.92to22.91) | 16.45(11.18to23.14) | 16.73(11.39to23.37) | 17.03(11.73to23.8) | 0.18 (0.18 to 0.18) | 0.18 (0.18 to 0.18) |
| New Zealand | 17.18(12.14to23.8) | 17.78(12.8to24.43) | 18.08(12.97to24.75) | 18.08(12.92to24.66) | 0.26 (0.24 to 0.28) | 0.18 (0.15 to 0.2) |
| Nicaragua | 27.63(20.1to35.68) | 29.93(22.54to37.92) | 32.32(24.92to40.37) | 34.94(27.32to43.22) | 0.8 (0.79 to 0.8) | 0.8 (0.79 to 0.81) |
| Niger | 15.04(9.54to22.08) | 15.73(10.18to22.74) | 16.35(10.7to23.39) | 16.83(11.13to23.79) | 0.42 (0.41 to 0.43) | 0.39 (0.38 to 0.4) |
| Nigeria | 15.7(10.24to22.62) | 16.26(10.8to23.19) | 16.99(11.44to23.93) | 18.1(12.32to25.02) | 0.4 (0.39 to 0.41) | 0.47 (0.45 to 0.5) |
| Niue | 27.54(19.5to35.9) | 30.18(22.23to38.64) | 32.04(24.09to40.53) | 33.09(24.94to41.45) | 0.77 (0.73 to 0.81) | 0.63 (0.59 to 0.67) |
| North Macedonia | 18.27(12.24to25.46) | 20.69(14.44to28.16) | 22.04(15.62to29.71) | 22.49(16.06to30.02) | 0.97 (0.9 to 1.04) | 0.7 (0.62 to 0.77) |
| Northern Mariana Islands | 29.16(21.33to37.71) | 32.57(24.48to41.21) | 34.02(25.92to42.56) | 33.34(25.38to41.76) | 0.79 (0.71 to 0.87) | 0.46 (0.36 to 0.55) |
| Norway | 13.94(9.16to20.36) | 14.36(9.53to20.82) | 14.66(9.81to21.13) | 14.81(9.97to21.32) | 0.26 (0.25 to 0.27) | 0.21 (0.2 to 0.22) |
| Oman | 17.23(11.98to24.02) | 19.63(14.06to26.68) | 22.51(16.52to29.53) | 25.68(19.32to32.98) | 1.36 (1.35 to 1.37) | 1.39 (1.38 to 1.4) |
| Pakistan | 20.06(13.52to27.69) | 21.09(14.37to28.94) | 22.08(15.1to30.06) | 22.99(15.93to30.89) | 0.49 (0.48 to 0.5) | 0.47 (0.46 to 0.47) |
| Palau | 28.9(20.94to37.4) | 33.14(25.04to41.65) | 35(26.75to43.51) | 34.29(26.07to42.74) | 0.98 (0.88 to 1.08) | 0.58 (0.47 to 0.7) |
| Palestine | 20.01(14.32to27.1) | 21.44(15.72to28.61) | 23.72(17.7to31.08) | 26.86(20.73to34.2) | 0.87 (0.83 to 0.91) | 1.02 (0.97 to 1.07) |
| Panama | 25.64(18.33to33.62) | 27.24(19.84to35.34) | 28.9(21.5to37.03) | 30.49(23.41to38.91) | 0.6 (0.6 to 0.6) | 0.6 (0.6 to 0.6) |
| Papua New Guinea | 21.25(14.16to29.44) | 21.81(14.56to29.97) | 22.67(15.39to31.1) | 23.88(16.38to32.41) | 0.33 (0.31 to 0.34) | 0.4 (0.38 to 0.42) |
| Paraguay | 18.6(12.82to25.93) | 20.03(13.98to27.53) | 21.8(15.59to29.16) | 23.75(17.13to31.5) | 0.8 (0.79 to 0.81) | 0.85 (0.83 to 0.86) |
| Peru | 15.89(10.4to22.87) | 17.16(11.5to24.14) | 18.64(12.74to25.7) | 20.19(13.98to27.6) | 0.8 (0.8 to 0.81) | 0.83 (0.82 to 0.84) |
| Philippines | 24.95(17.71to33.15) | 26.58(19.01to34.93) | 28.26(20.46to36.71) | 29.86(21.97to38.42) | 0.63 (0.63 to 0.63) | 0.62 (0.62 to 0.62) |
| Poland | 18.66(12.63to25.79) | 19.21(13.22to26.27) | 19.52(13.59to26.55) | 19.63(13.66to26.72) | 0.23 (0.22 to 0.25) | 0.17 (0.15 to 0.19) |
| Portugal | 15.32(10.29to21.7) | 16.94(11.66to23.61) | 17.26(11.92to24.07) | 15.9(10.86to22.5) | 0.59 (0.49 to 0.69) | 0.16 (0.02 to 0.29) |
| Puerto Rico | 23.62(16.75to31.59) | 27.2(20.47to35.34) | 28.98(22.02to37.3) | 29.35(22.04to37.65) | 1.07 (0.98 to 1.16) | 0.72 (0.62 to 0.82) |
| Qatar | 22.22(16.37to29.35) | 25.42(19.31to32.48) | 27.46(21.21to34.78) | 28.81(22.35to36.2) | 1.08 (1.01 to 1.15) | 0.86 (0.8 to 0.93) |
| Republic of Korea | 17.49(12.18to24.33) | 17.73(12.55to24.56) | 17.66(12.65to24.5) | 17.11(12.09to23.73) | 0.05 (0.03 to 0.07) | -0.06 (-0.09 to -0.03) |
| Republic of Moldova | 20.07(13.19to28.02) | 20.7(13.71to28.75) | 21.36(14.3to29.46) | 22.31(15.2to30.36) | 0.32 (0.31 to 0.32) | 0.35 (0.33 to 0.36) |
| Romania | 16.87(11.02to23.8) | 17.85(12.1to24.94) | 18.98(13.13to26.12) | 20.22(14.11to27.42) | 0.59 (0.58 to 0.6) | 0.62 (0.61 to 0.63) |
| Russian Federation | 23.46(16.24to31.61) | 23.85(16.59to32.01) | 24.46(17.2to32.66) | 25.45(18.06to33.67) | 0.21 (0.2 to 0.22) | 0.27 (0.25 to 0.29) |
| Rwanda | 15.35(9.63to22.33) | 15.76(9.98to22.67) | 16.3(10.47to23.2) | 16.98(11.08to24.1) | 0.3 (0.29 to 0.31) | 0.35 (0.33 to 0.36) |
| Saint Kitts and Nevis | 25.6(18.7to33.63) | 28.99(21.92to37.16) | 30.65(23.41to38.67) | 30.96(23.7to39.01) | 0.94 (0.86 to 1.02) | 0.63 (0.54 to 0.72) |
| Saint Lucia | 22.88(15.93to30.92) | 25.44(18.22to33.51) | 26.98(19.92to35.27) | 27.59(20.45to35.67) | 0.85 (0.79 to 0.91) | 0.63 (0.57 to 0.69) |
| Saint Vincent and the Grenadines | 21.87(15.1to29.49) | 24.2(17.13to32.09) | 25.74(18.63to33.83) | 26.52(19.28to34.63) | 0.84 (0.79 to 0.88) | 0.66 (0.6 to 0.71) |
| Samoa | 27.05(19.29to35.39) | 28.96(21.21to37.3) | 30.41(22.58to38.84) | 31.33(23.36to39.87) | 0.6 (0.57 to 0.62) | 0.5 (0.48 to 0.53) |
| San Marino | 14.37(9.46to20.98) | 14.65(9.76to21.28) | 14.83(9.93to21.44) | 14.91(10.03to21.53) | 0.16 (0.15 to 0.17) | 0.13 (0.12 to 0.14) |
| Sao Tome and Principe | 17.22(11.58to24.26) | 18.58(12.72to25.8) | 19.73(13.76to27.09) | 20.61(14.44to28.07) | 0.69 (0.67 to 0.71) | 0.62 (0.6 to 0.64) |
| Saudi Arabia | 22.12(16.18to29.3) | 26.29(19.9to33.75) | 29.44(22.97to37.13) | 31.88(25.07to39.52) | 1.49 (1.41 to 1.56) | 1.23 (1.16 to 1.3) |
| Senegal | 15.79(10.26to22.79) | 16.36(10.69to23.44) | 16.92(11.23to24.01) | 17.45(11.67to24.44) | 0.35 (0.34 to 0.35) | 0.34 (0.34 to 0.34) |
| Serbia | 18.11(12.09to25.54) | 19.28(13.17to26.73) | 20.29(14.09to27.57) | 21.09(14.92to28.52) | 0.58 (0.56 to 0.59) | 0.52 (0.51 to 0.54) |
| Seychelles | 26.19(18.58to34.35) | 28.49(20.91to36.76) | 30.11(22.26to38.57) | 31.01(23.24to39.48) | 0.71 (0.68 to 0.75) | 0.58 (0.54 to 0.62) |
| Sierra Leone | 15.43(9.88to22.49) | 15.97(10.35to23.09) | 16.7(11.01to23.81) | 17.61(11.76to24.69) | 0.4 (0.38 to 0.41) | 0.45 (0.44 to 0.47) |
| Singapore | 23.07(16.76to30.38) | 22.56(16.43to30) | 22.39(16.41to29.84) | 22.58(16.56to29.87) | -0.16 (-0.17 to -0.14) | -0.08 (-0.1 to -0.05) |
| Slovakia | 18.3(12.3to25.53) | 19.25(13.18to26.65) | 20.03(13.9to27.36) | 20.6(14.36to27.98) | 0.46 (0.45 to 0.47) | 0.41 (0.39 to 0.42) |
| Slovenia | 17.13(11.38to24.24) | 18.03(12.18to25.16) | 18.77(12.8to25.88) | 19.34(13.37to26.47) | 0.46 (0.45 to 0.47) | 0.41 (0.4 to 0.43) |
| Solomon Islands | 26.04(18.28to34.41) | 26.66(18.79to35) | 27.51(19.61to35.86) | 28.55(20.65to36.83) | 0.28 (0.27 to 0.28) | 0.32 (0.3 to 0.33) |
| Somalia | 15.29(9.52to22.34) | 15.63(9.9to22.71) | 16.09(10.41to23.13) | 16.65(10.67to23.86) | 0.26 (0.25 to 0.26) | 0.29 (0.28 to 0.3) |
| South Africa | 19.39(13.13to26.89) | 20(13.59to27.57) | 21.04(14.47to28.71) | 22.59(15.87to30.18) | 0.41 (0.39 to 0.44) | 0.52 (0.49 to 0.55) |
| South Sudan | 14.98(9.36to21.98) | 15.4(9.7to22.4) | 15.89(10.15to22.89) | 16.46(10.58to23.43) | 0.3 (0.29 to 0.3) | 0.32 (0.31 to 0.33) |
| Spain | 15.06(10.33to21.42) | 15.12(10.41to21.61) | 14.94(10.28to21.37) | 14.23(9.6to20.44) | -0.04 (-0.06 to -0.02) | -0.17 (-0.21 to -0.13) |
| Sri Lanka | 24.72(17.39to33.26) | 26.97(19.51to35.6) | 28.83(21.1to37.5) | 30.18(22.39to38.67) | 0.78 (0.76 to 0.81) | 0.69 (0.66 to 0.71) |
| Sudan | 18.3(12.75to25.26) | 19.86(14.18to26.97) | 21.88(15.85to29.12) | 24.47(18.27to31.96) | 0.91 (0.89 to 0.93) | 1 (0.97 to 1.03) |
| Suriname | 21.7(15.04to29.37) | 23.83(16.84to31.64) | 25.66(18.66to33.6) | 27.06(20.15to35.3) | 0.85 (0.82 to 0.87) | 0.76 (0.73 to 0.78) |
| Sweden | 16.93(11.36to23.88) | 17.09(11.53to23.95) | 17.11(11.57to23.93) | 16.97(11.46to23.85) | 0.05 (0.04 to 0.06) | 0.01 (-0.01 to 0.02) |
| Switzerland | 16.51(11.29to23.19) | 16.57(11.48to23.24) | 16.59(11.51to23.17) | 16.58(11.52to23.29) | 0.03 (0.02 to 0.03) | 0.01 (0.01 to 0.02) |
| Syrian Arab Republic | 21.11(15.19to28.27) | 24.48(18.29to31.9) | 26.32(19.91to34.03) | 26.73(20.13to34.42) | 1.13 (1.04 to 1.22) | 0.8 (0.7 to 0.89) |
| Taiwan (Province of China) | 24.2(17.38to32.24) | 25.3(18.39to33.23) | 26.62(19.61to34.49) | 28.33(21.03to36.45) | 0.48 (0.47 to 0.49) | 0.53 (0.52 to 0.55) |
| Tajikistan | 18.91(12.36to26.59) | 19.21(12.61to26.88) | 19.91(13.26to27.84) | 21.53(14.64to29.66) | 0.26 (0.23 to 0.28) | 0.42 (0.37 to 0.47) |
| Thailand | 26.91(19.09to35.37) | 28.24(20.6to36.7) | 29.27(21.57to37.66) | 30.46(22.66to39.13) | 0.43 (0.42 to 0.45) | 0.41 (0.4 to 0.42) |
| Timor-Leste | 22.86(15.7to31.04) | 24.11(16.89to32.3) | 25.34(18.12to33.72) | 26.5(18.92to34.82) | 0.52 (0.52 to 0.52) | 0.51 (0.5 to 0.51) |
| Togo | 15.98(10.4to23.05) | 16.52(10.86to23.56) | 17.25(11.47to24.28) | 18.13(12.23to25.18) | 0.39 (0.38 to 0.4) | 0.44 (0.42 to 0.45) |
| Tokelau | 25.14(17.47to33.39) | 27.09(19.26to35.3) | 28.89(21.02to37.14) | 30.57(22.5to38.92) | 0.7 (0.68 to 0.72) | 0.67 (0.66 to 0.68) |
| Tonga | 27.01(19.29to35.23) | 28.47(20.56to36.68) | 29.85(21.79to38.18) | 31.1(23.24to39.55) | 0.5 (0.5 to 0.51) | 0.48 (0.48 to 0.49) |
| Trinidad and Tobago | 21.53(14.71to29.04) | 24.07(17.11to31.79) | 25.93(18.88to34.02) | 27.05(19.94to35.01) | 0.95 (0.9 to 0.99) | 0.78 (0.73 to 0.83) |
| Tunisia | 19.76(14.06to26.72) | 22.59(16.74to29.88) | 24.87(18.68to32.35) | 26.61(20.3to34.14) | 1.19 (1.14 to 1.24) | 1.01 (0.96 to 1.06) |
| Turkey | 19.07(13.43to25.99) | 23.78(17.53to31.02) | 25.97(19.54to33.88) | 25.84(19.67to33.24) | 1.6 (1.44 to 1.76) | 1.01 (0.84 to 1.17) |
| Turkmenistan | 21.09(14.22to29.12) | 21.84(14.91to29.91) | 23.02(15.84to31.38) | 24.71(17.42to33.23) | 0.44 (0.42 to 0.46) | 0.54 (0.51 to 0.57) |
| Tuvalu | 25.22(17.58to33.59) | 27.11(19.3to35.35) | 28.8(20.94to37.1) | 30.19(22.23to38.68) | 0.67 (0.66 to 0.69) | 0.62 (0.6 to 0.63) |
| Uganda | 15.01(9.4to21.92) | 15.84(10.03to22.68) | 16.31(10.37to23.31) | 16.47(10.61to23.57) | 0.43 (0.4 to 0.46) | 0.31 (0.28 to 0.35) |
| Ukraine | 20.99(14.09to28.92) | 21.2(14.27to29.13) | 21.58(14.53to29.59) | 22.49(15.35to30.63) | 0.14 (0.13 to 0.15) | 0.22 (0.19 to 0.24) |
| United Arab Emirates | 23.46(17.34to30.67) | 26.09(19.82to33.36) | 28.06(21.59to35.54) | 29.66(23.08to36.95) | 0.92 (0.88 to 0.96) | 0.78 (0.75 to 0.82) |
| United Kingdom | 14.45(9.77to20.93) | 14.76(10.08to21.22) | 14.83(10.11to21.33) | 14.73(10.03to21.21) | 0.13 (0.11 to 0.15) | 0.06 (0.04 to 0.08) |
| United Republic of Tanzania | 15.35(9.68to22.36) | 16.14(10.35to23.19) | 17(11.08to24.2) | 17.87(11.78to25.19) | 0.51 (0.51 to 0.52) | 0.52 (0.52 to 0.53) |
| United States of America | 20.51(14.74to27.25) | 20.91(14.98to27.87) | 21.14(15.24to28.25) | 21.54(15.61to28.63) | 0.16 (0.15 to 0.17) | 0.15 (0.14 to 0.16) |
| United States Virgin Islands | 22.12(15.29to30.25) | 25.17(18.03to33.3) | 26.88(19.6to34.92) | 27.44(20.29to35.49) | 1.01 (0.93 to 1.09) | 0.72 (0.64 to 0.8) |
| Uruguay | 16.35(11.1to23.07) | 16.87(11.61to23.64) | 17.33(11.9to24.11) | 17.7(12.3to24.44) | 0.29 (0.29 to 0.3) | 0.27 (0.27 to 0.28) |
| Uzbekistan | 21.97(15.08to30.02) | 22.88(15.82to31.06) | 24.13(16.9to32.45) | 25.7(18.22to33.9) | 0.47 (0.46 to 0.49) | 0.54 (0.52 to 0.56) |
| Vanuatu | 24.4(16.79to32.81) | 25.56(17.83to33.97) | 26.95(19.15to35.37) | 28.42(20.62to36.97) | 0.5 (0.49 to 0.51) | 0.53 (0.52 to 0.53) |
| Venezuela (Bolivarian Republic of) | 26.52(18.95to34.57) | 29.32(21.66to37.37) | 30.89(23.23to39.18) | 31.45(24.06to39.89) | 0.79 (0.74 to 0.85) | 0.57 (0.51 to 0.64) |
| Viet Nam | 22.35(15.32to30.61) | 23.83(16.61to32.14) | 24.95(17.67to33.34) | 25.81(18.35to34.16) | 0.56 (0.54 to 0.58) | 0.49 (0.47 to 0.51) |
| Yemen | 17.96(12.55to25.03) | 19.55(13.94to26.59) | 21.23(15.46to28.29) | 23.04(16.92to30.41) | 0.85 (0.84 to 0.85) | 0.85 (0.85 to 0.86) |
| Zambia | 15.71(10.02to22.73) | 16.21(10.4to23.38) | 17.01(11.1to24.4) | 18.11(11.97to25.65) | 0.4 (0.38 to 0.42) | 0.49 (0.46 to 0.51) |
| Zimbabwe | 17.89(11.95to25.16) | 17.9(11.87to25.24) | 18.09(12.01to25.54) | 19.93(13.6to27.46) | 0.05 (0.04 to 0.06) | 0.28 (0.2 to 0.36) |

SEV, summary exposure value; EAPC, estimated annual percentage change.
