# Supplementary material for: Global, regional, and national burden of kidney dysfunction from 1990 to 2019: a systematic analysis from the global burden of disease study 2019
Source: BMC Public Health. 2023 Jun 23;23:1218. doi: 10.1186/s12889-023-16130-8 (PMC10288715; doi:10.1186/s12889-023-16130-8)
Supplement: Supplementary file 9 — Additional file 9: Table 4S. Age-standardized morality of kidney dysfunction for both sexes combined in 1990,2000,2010, and 2019, and EAPC of ASMR from 1990 to 2019 and 1990 to 2010 in 204 countries and territories. [file 12889_2023_16130_MOESM9_ESM.docx]

Table 4S. Age-standardized morality of kidney dysfunction for both sexes combined in 1990,2000,2010, and 2019, and EAPC of ASMR from 1990 to 2019 and 1990 to 2010 in 204 countries and territories

| Location | ASMR 1990 | ASMR 2000 | ASMR 2010 | ASMR 2019 | EAPC 1990-2010 | EAPC 1990-2019 |
| --- | --- | --- | --- | --- | --- | --- |
| Afghanistan | 116.83(90.64to150.03) | 119.77(93.04to157.2) | 116.83(91to149.58) | 118.86(92.37to150.28) | 0.12 (0.03 to 0.21) | 0.07 (0.01 to 0.12) |
| Albania | 34.76(28.64to41.55) | 32.81(26.69to39.23) | 32.83(25.37to41.37) | 34.37(24.87to45.34) | 0.12 (-0.22 to 0.45) | 0.18 (0.01 to 0.34) |
| Algeria | 105.68(83.45to130.65) | 96.89(75.48to122.28) | 91.02(71.1to111.89) | 90.42(71.06to112.58) | -0.64 (-0.69 to -0.59) | -0.49 (-0.54 to -0.44) |
| American Samoa | 72.22(62.6to82.76) | 84.25(74.55to94.41) | 90.03(80.2to101.01) | 95.3(81.71to111.9) | 1.2 (1.05 to 1.35) | 1 (0.9 to 1.1) |
| Andorra | 23.38(17.85to30.93) | 19.55(15.11to25.7) | 17.5(13.43to22.2) | 17.41(12.95to22.44) | -1.59 (-1.7 to -1.49) | -1.05 (-1.21 to -0.89) |
| Angola | 42.13(32.56to51.78) | 42.62(33.37to51.57) | 41.05(31.82to50.28) | 40.64(30.89to51.48) | -0.15 (-0.22 to -0.09) | -0.23 (-0.27 to -0.18) |
| Antigua and Barbuda | 50.55(44.38to57.2) | 51.79(45.47to58.09) | 57.85(51.38to64.76) | 60.51(51.86to69.99) | 0.48 (0.13 to 0.82) | 0.69 (0.51 to 0.87) |
| Argentina | 49.1(42.21to56.25) | 49.12(43.59to54.56) | 43.78(39.05to48.19) | 42.92(38.15to47.53) | -0.31 (-0.59 to -0.03) | -0.5 (-0.65 to -0.35) |
| Armenia | 39.01(25.8to54.03) | 44.48(30.58to60.23) | 43.7(32.51to56.36) | 44.36(32.3to57.92) | -0.15 (-0.51 to 0.21) | -0.11 (-0.29 to 0.07) |
| Australia | 37.16(29.63to45.22) | 28.5(22.9to34.45) | 22.1(18.07to26.14) | 21.17(17.36to24.98) | -2.63 (-2.73 to -2.53) | -2.18 (-2.35 to -2.01) |
| Austria | 30.84(24.04to38.06) | 31.29(24.86to38.5) | 30.4(24.96to36.13) | 29(23.83to33.96) | 0.03 (-0.1 to 0.15) | -0.14 (-0.23 to -0.06) |
| Azerbaijan | 47.89(34.56to62.58) | 61.44(44.23to80.87) | 80.49(60.7to101.79) | 85.77(63.72to109.34) | 2.28 (1.97 to 2.58) | 2.1 (1.92 to 2.28) |
| Bahamas | 47.13(40.73to53.69) | 47.86(41.96to54.39) | 49.03(43.76to54.76) | 49.7(40.74to59.97) | 0.31 (0.11 to 0.5) | 0.3 (0.2 to 0.39) |
| Bahrain | 115.96(93.18to140.7) | 100.13(83.34to118.75) | 99.72(85.28to116.08) | 77.18(60.92to94.6) | -0.38 (-0.91 to 0.15) | -1.42 (-1.82 to -1.02) |
| Bangladesh | 31.1(24.85to37.9) | 30.85(25.13to37.03) | 35.02(29.12to41.68) | 29.88(22.52to38.69) | 1.37 (1.04 to 1.71) | 0.21 (-0.14 to 0.57) |
| Barbados | 40.37(34.58to46.37) | 40.97(36.33to46.01) | 39.95(35.63to44.14) | 42.7(35.3to50.61) | 0 (-0.17 to 0.17) | 0.02 (-0.07 to 0.11) |
| Belarus | 42.9(28.07to59.47) | 51.1(33.5to71.44) | 52.62(34.74to72.59) | 47.48(30.63to67.83) | 0.79 (0.41 to 1.17) | 0.09 (-0.17 to 0.36) |
| Belgium | 31.43(25.52to37.79) | 26.13(21.49to31.05) | 21.75(18.16to25.27) | 19.28(16to22.34) | -1.68 (-1.77 to -1.59) | -1.74 (-1.8 to -1.68) |
| Belize | 43.48(37.46to50.26) | 65.06(57.76to72.75) | 57.51(52.16to62.79) | 58.03(50.11to67.37) | 1.86 (1.15 to 2.57) | 0.95 (0.54 to 1.37) |
| Benin | 52.79(45.89to60.88) | 52.54(43.91to61.97) | 54.31(44.35to66.37) | 51.4(41.62to63.44) | 0.13 (0.07 to 0.19) | 0.01 (-0.05 to 0.07) |
| Bermuda | 50.13(40.52to60.19) | 38.67(32.38to45.57) | 30.88(25.82to36.4) | 30.01(24.39to36.61) | -2.34 (-2.47 to -2.22) | -1.75 (-1.93 to -1.56) |
| Bhutan | 39.03(28.55to50.64) | 42.17(33.5to51.81) | 46.38(36.75to55.84) | 49.16(38.19to60.3) | 0.93 (0.86 to 1) | 0.88 (0.84 to 0.92) |
| Bolivia (Plurinational State of) | 58.3(49.32to71.12) | 63.87(54.74to76.96) | 68.7(57.47to82.94) | 74.77(60.35to92.58) | 0.8 (0.74 to 0.85) | 0.9 (0.86 to 0.94) |
| Bosnia and Herzegovina | 38.12(29.61to47.47) | 37.48(29.99to46.16) | 40.63(33.01to48.42) | 41.47(31.34to53.63) | 0.37 (0.03 to 0.72) | 0.16 (-0.01 to 0.34) |
| Botswana | 49.05(38.11to62.99) | 67.54(48.02to91.02) | 62.73(46.53to81.14) | 64.66(48.75to84.77) | 1.12 (0.57 to 1.67) | 0.57 (0.27 to 0.87) |
| Brazil | 44.98(39.14to50.94) | 39.05(34.57to43.56) | 34.2(30.38to37.9) | 32.24(28.8to35.78) | -1.33 (-1.4 to -1.26) | -1.15 (-1.22 to -1.08) |
| Brunei Darussalam | 83.11(70.97to96.81) | 72.36(62.2to84.17) | 70.26(60.84to80) | 65.75(56.58to75.98) | -0.49 (-0.77 to -0.21) | -0.42 (-0.57 to -0.28) |
| Bulgaria | 57.63(42.99to73.54) | 68.52(52.37to85.81) | 58.45(47.06to70.43) | 58.35(44.04to74.53) | -0.25 (-0.79 to 0.29) | -0.59 (-0.87 to -0.3) |
| Burkina Faso | 42.86(35.38to50.85) | 45.08(38.17to52.48) | 47.09(39.53to54.73) | 49.19(40.97to59.13) | 0.56 (0.5 to 0.61) | 0.56 (0.53 to 0.59) |
| Burundi | 49.75(40.03to60.41) | 47.82(39.03to58.04) | 42.51(34.75to51.18) | 40.79(32.43to51.04) | -0.98 (-1.12 to -0.83) | -0.91 (-0.98 to -0.83) |
| Cabo Verde | 28.66(23.91to33.7) | 32.13(26.1to38.85) | 31.21(27to35.83) | 44.95(37.97to52.25) | 0.47 (0.2 to 0.73) | 0.89 (0.6 to 1.19) |
| Cambodia | 47.91(39.81to57.51) | 46.19(38.95to54.45) | 44.32(36.81to52.79) | 46.5(36.94to55.57) | -0.5 (-0.56 to -0.44) | -0.22 (-0.31 to -0.13) |
| Cameroon | 63.37(52.08to75.86) | 67.15(54.4to80.5) | 68.74(55.59to83.09) | 64.81(51.38to81.3) | 0.48 (0.41 to 0.55) | 0.17 (0.06 to 0.27) |
| Canada | 32.65(25.84to39.42) | 27.72(22.51to33.38) | 21.04(17.43to24.66) | 19.87(16.38to23.3) | -2.16 (-2.32 to -1.99) | -2.03 (-2.16 to -1.91) |
| Central African Republic | 50.13(39.85to60.99) | 51.5(39.68to64.58) | 52.39(39.63to67.27) | 51.12(38.39to66.94) | 0.27 (0.21 to 0.33) | 0.15 (0.09 to 0.2) |
| Chad | 46.42(37.04to60.35) | 48.27(38.67to62.25) | 50.96(41.29to62.42) | 49.15(39.08to60.76) | 0.49 (0.45 to 0.52) | 0.28 (0.21 to 0.35) |
| Chile | 37.31(31.71to43.45) | 34.69(30.62to38.8) | 35.09(31.23to38.85) | 32.31(28.5to36.05) | 0.17 (-0.08 to 0.41) | -0.17 (-0.33 to -0.02) |
| China | 35.77(30.07to42.15) | 33.83(28.76to39.12) | 39.08(32.27to46.47) | 33.83(27.48to41.22) | 0.79 (0.45 to 1.13) | 0.39 (0.16 to 0.61) |
| Colombia | 47.51(40.63to54.98) | 39.49(34.03to44.99) | 35.46(30.59to40.58) | 32.73(25.12to41.33) | -1.7 (-1.91 to -1.49) | -1.43 (-1.56 to -1.29) |
| Comoros | 44.14(30.72to54.38) | 43.99(36.38to52.21) | 40.24(33.61to47.93) | 41.52(34.3to50.48) | -0.63 (-0.8 to -0.46) | -0.36 (-0.48 to -0.25) |
| Congo | 58.6(44.84to73.41) | 56.33(43.32to69.31) | 51.83(38.47to64.75) | 51.98(38.5to65.86) | -0.72 (-0.89 to -0.55) | -0.54 (-0.64 to -0.44) |
| Cook Islands | 49.63(41.92to59.23) | 49.08(41.93to57.37) | 49.65(42.93to56.81) | 52.98(44.25to63.35) | -0.04 (-0.1 to 0.03) | 0.29 (0.19 to 0.39) |
| Costa Rica | 48.07(40.43to56.01) | 50.02(43.63to56.57) | 47.12(41.6to52.28) | 44.77(34.84to56.43) | -0.41 (-0.77 to -0.05) | -0.47 (-0.66 to -0.29) |
| Croatia | 46.41(35.55to58.37) | 44.13(35.21to53.41) | 40.62(32.44to49.36) | 35.9(26.76to46.62) | -0.33 (-0.5 to -0.15) | -0.79 (-0.94 to -0.63) |
| Cuba | 32.5(25.16to40.38) | 31.29(25.42to37.64) | 32.77(28.1to37.76) | 34.17(26.75to42.64) | -0.07 (-0.22 to 0.09) | 0.11 (-0.02 to 0.24) |
| Cyprus | 74.36(62.8to87.22) | 63.91(54.09to74.63) | 47.98(40.52to56.2) | 37.91(31.37to45.36) | -2.73 (-3.03 to -2.43) | -2.77 (-2.92 to -2.63) |
| Czechia | 50.24(38.23to63.14) | 41.03(31.64to50.95) | 31.78(24.2to39.92) | 28.48(20.59to37.22) | -2.13 (-2.26 to -1.99) | -2.11 (-2.2 to -2.02) |
| C么te d'Ivoire | 59.74(50.12to70.25) | 62.21(52.14to72.95) | 59.05(48.64to70.35) | 54.17(44.05to65.23) | -0.05 (-0.17 to 0.07) | -0.37 (-0.48 to -0.26) |
| Democratic People's Republic of Korea | 41.61(32.94to50.57) | 44.94(35.98to54.25) | 48.29(39.17to57.73) | 44.12(35.75to54.19) | 0.79 (0.77 to 0.82) | 0.32 (0.18 to 0.47) |
| Democratic Republic of the Congo | 46.08(38.04to55.09) | 43.9(36.82to52.13) | 41.15(33to49.95) | 40.7(31.57to52.01) | -0.58 (-0.62 to -0.53) | -0.52 (-0.55 to -0.48) |
| Denmark | 31.84(24.32to40) | 26.55(21.32to32.14) | 21.22(17.9to24.75) | 18.53(15.54to21.61) | -2.27 (-2.46 to -2.07) | -2.2 (-2.31 to -2.08) |
| Djibouti | 37.37(29.17to47.19) | 40.5(30.73to51.45) | 44.03(33to56.24) | 45.36(36.07to57.7) | 0.82 (0.77 to 0.87) | 0.67 (0.61 to 0.72) |
| Dominica | 61.29(52.95to70.26) | 61.94(52.7to71.61) | 65.91(57.64to75.29) | 68.9(57.35to82.69) | 0.51 (0.36 to 0.66) | 0.61 (0.52 to 0.69) |
| Dominican Republic | 31.74(26.25to37.93) | 34.38(29.38to39.68) | 48.62(39.21to59.73) | 54.36(41.49to69.19) | 2.88 (2.44 to 3.31) | 2.75 (2.5 to 3) |
| Ecuador | 34.59(30.6to38.72) | 50.98(46.31to55.29) | 71.07(63.44to78.44) | 66.98(53.86to83.08) | 4.18 (3.83 to 4.52) | 2.65 (2.19 to 3.12) |
| Egypt | 102.82(83.58to122.99) | 107.81(86.9to128.34) | 125.71(101.99to147.44) | 128.66(96.68to163.5) | 1.19 (1 to 1.39) | 0.95 (0.83 to 1.07) |
| El Salvador | 41.94(36.46to47.9) | 67.73(61.72to74.09) | 87.23(80.13to93.62) | 89.82(69.09to114.91) | 4.34 (3.97 to 4.7) | 2.94 (2.5 to 3.37) |
| Equatorial Guinea | 46.48(35.41to58.91) | 43.16(33.04to54.93) | 44.51(32.72to59.33) | 48.18(35.73to63.52) | -0.35 (-0.52 to -0.17) | 0.15 (-0.02 to 0.31) |
| Eritrea | 38.35(27.49to52.53) | 42.79(32.15to55.28) | 43.29(32.62to56.35) | 44.43(33.78to58.47) | 0.69 (0.54 to 0.85) | 0.42 (0.31 to 0.52) |
| Estonia | 53.14(36.24to72.57) | 50.07(35.72to66.86) | 34.75(25.64to45.62) | 33.31(23.9to45.75) | -2.24 (-2.65 to -1.82) | -2.16 (-2.38 to -1.93) |
| Eswatini | 58.81(48.57to69.54) | 77.67(59.09to100.55) | 85.61(64.77to108.83) | 77.22(58.4to98.93) | 2.64 (2.17 to 3.11) | 1.15 (0.69 to 1.62) |
| Ethiopia | 54.22(44.94to63.06) | 47.03(40.94to53.39) | 39.19(34.69to44.31) | 37.9(31.65to45.08) | -1.68 (-1.74 to -1.62) | -1.44 (-1.54 to -1.35) |
| Fiji | 83.97(66.67to105.8) | 109.44(94.99to125.56) | 95.48(82.49to108.4) | 96.35(76.15to120.99) | 0.44 (-0.01 to 0.9) | 0.06 (-0.18 to 0.3) |
| Finland | 28.67(20.98to36.45) | 23.79(17.68to30.48) | 20.96(15.97to26.2) | 18.31(14.15to23.02) | -1.48 (-1.58 to -1.37) | -1.53 (-1.59 to -1.47) |
| France | 20.78(17.29to24.59) | 17.79(14.75to21.03) | 14.55(11.99to17.02) | 12.78(10.44to15.09) | -1.59 (-1.74 to -1.44) | -1.75 (-1.84 to -1.65) |
| Gabon | 53.13(40.93to65.39) | 60.05(44.07to72.98) | 61.07(43.87to75.69) | 59.96(41.49to75.99) | 0.93 (0.78 to 1.08) | 0.39 (0.22 to 0.55) |
| Gambia | 48.28(38.18to59.96) | 48.02(39.13to58.29) | 52.31(42.65to62.37) | 55.73(45.02to67.9) | 0.55 (0.36 to 0.75) | 0.49 (0.37 to 0.61) |
| Georgia | 58.52(40.49to78.12) | 53.69(39.27to70.75) | 51.16(40.61to63.12) | 47.81(36.91to60.38) | -0.42 (-0.84 to 0.01) | -0.86 (-1.13 to -0.6) |
| Germany | 37.94(29.72to47.22) | 31.07(25.15to37.67) | 27.87(23.27to32.42) | 28.3(23.63to33.02) | -1.54 (-1.65 to -1.43) | -0.92 (-1.11 to -0.74) |
| Ghana | 45.39(36.45to56.19) | 47.43(38.71to56.64) | 53.67(44.01to63.87) | 53.51(42.77to65.66) | 0.99 (0.87 to 1.11) | 0.74 (0.64 to 0.83) |
| Greece | 49.72(41.9to57.53) | 43.32(36.2to50.65) | 31.19(25.7to36.77) | 31.16(26.35to36.2) | -2.09 (-2.36 to -1.82) | -2.05 (-2.24 to -1.86) |
| Greenland | 44.52(36.43to53.83) | 43.12(35.93to51.36) | 33.32(28.4to39.13) | 30.43(24.46to36.72) | -1.95 (-2.37 to -1.54) | -1.75 (-1.96 to -1.54) |
| Grenada | 69.69(61.07to79.06) | 66.42(59.72to73.88) | 73.96(67.06to81.21) | 74.99(66.43to83.69) | 0.27 (0 to 0.55) | 0.41 (0.26 to 0.56) |
| Guam | 56.05(46.53to67.08) | 54.44(45.32to64.28) | 51.99(43.67to61.28) | 60.81(49.22to73.56) | -0.32 (-0.7 to 0.06) | 0.32 (0.05 to 0.58) |
| Guatemala | 59.09(50.68to68.76) | 61.64(52.66to70.84) | 79.39(68.94to90.67) | 81.69(65.74to100.4) | 0.97 (0.55 to 1.4) | 1.39 (1.13 to 1.65) |
| Guinea | 51.83(42.06to64.57) | 49.37(40.58to60.86) | 54.19(44.6to64.91) | 53.3(42.22to65.55) | 0.26 (0.09 to 0.43) | 0.35 (0.26 to 0.45) |
| Guinea-Bissau | 72.52(58.95to87.5) | 67.61(55.61to80.5) | 69.32(57.74to82.25) | 65.63(52.11to80.61) | -0.2 (-0.32 to -0.08) | -0.23 (-0.3 to -0.17) |
| Guyana | 79.27(65.5to93.88) | 76.53(65.47to89.59) | 88.52(77.19to101.55) | 90.16(70.93to113.71) | 0.65 (0.41 to 0.89) | 0.74 (0.6 to 0.87) |
| Haiti | 71.68(58.29to90.36) | 64.65(51.97to81.85) | 70.48(55.14to89.44) | 71.36(53.75to94.86) | -0.01 (-0.25 to 0.23) | 0.26 (0.12 to 0.4) |
| Honduras | 48.06(38.13to64.03) | 66.5(50.49to87.13) | 82.01(63.5to103.55) | 91.88(76.14to112.28) | 2.78 (2.48 to 3.08) | 2.55 (2.33 to 2.77) |
| Hungary | 40.64(30.77to51.67) | 39.6(30.14to50.09) | 38.47(29.94to47.66) | 36.45(27.28to47.06) | -0.43 (-0.56 to -0.31) | -0.4 (-0.47 to -0.34) |
| Iceland | 24.49(18.66to31.13) | 20.15(15.51to25.27) | 15.91(12.4to19.7) | 13.6(10.51to16.9) | -2.21 (-2.35 to -2.08) | -2.34 (-2.44 to -2.24) |
| India | 50.72(41.34to60) | 55.01(46.01to64.04) | 47.47(39.92to55.63) | 45.31(36.2to54.64) | -0.16 (-0.46 to 0.14) | -0.56 (-0.74 to -0.38) |
| Indonesia | 47.19(39.9to55.54) | 50.88(43.24to59.04) | 56.27(47.58to64.99) | 56(45.8to66.26) | 0.93 (0.88 to 0.97) | 0.7 (0.63 to 0.78) |
| Iran (Islamic Republic of) | 78.41(65.03to93.07) | 78.93(65.4to93.29) | 60.04(50.29to70.4) | 58.15(48.84to68.06) | -1.26 (-1.62 to -0.91) | -1.44 (-1.66 to -1.23) |
| Iraq | 106.27(86.74to131.25) | 106.75(81.08to138.73) | 112.28(87.05to139.7) | 114.13(89.64to136.29) | 0.25 (0.17 to 0.34) | 0.16 (0.1 to 0.22) |
| Ireland | 47.57(38.38to57.17) | 37.77(30.87to45.25) | 23.23(18.98to27.69) | 21.25(17.33to25.07) | -3.59 (-3.88 to -3.29) | -3.21 (-3.41 to -3) |
| Israel | 50.91(42.82to59.36) | 42.77(37.06to48.84) | 34.33(30.04to38.14) | 31.02(26.66to34.86) | -1.75 (-1.96 to -1.55) | -1.86 (-1.97 to -1.75) |
| Italy | 29.35(24.14to34.66) | 23.41(19.41to27.74) | 19.31(15.73to22.74) | 17.89(14.6to21.1) | -1.99 (-2.09 to -1.9) | -1.75 (-1.84 to -1.65) |
| Jamaica | 41.15(36.78to45.77) | 53.11(48.41to57.67) | 42.24(38.45to46.39) | 47.34(38.21to58.69) | 0 (-0.76 to 0.76) | 0.15 (-0.23 to 0.53) |
| Japan | 30.37(25.51to35.23) | 22.82(19.26to26.12) | 18.37(15.03to21.08) | 15.56(12.5to18.1) | -2.64 (-2.77 to -2.52) | -2.41 (-2.51 to -2.31) |
| Jordan | 87.73(73.86to103.37) | 88.94(73.32to106.06) | 76.66(65.84to88.2) | 71.92(59.2to86.43) | -0.55 (-0.9 to -0.2) | -0.95 (-1.15 to -0.74) |
| Kazakhstan | 45.63(33.13to59.67) | 64.71(47.24to85.46) | 60.37(45.15to78.08) | 53.19(40.18to67.17) | 1.02 (0.37 to 1.68) | -0.05 (-0.48 to 0.38) |
| Kenya | 30.92(26.35to36.28) | 33.43(28.55to38.55) | 37(32.33to42.92) | 38.04(32.57to44.21) | 1.08 (0.96 to 1.2) | 0.81 (0.72 to 0.91) |
| Kiribati | 97.86(79.41to119.27) | 116.23(97.09to138.05) | 113.78(90.69to137.62) | 112.91(87.94to139.73) | 0.9 (0.65 to 1.15) | 0.3 (0.1 to 0.5) |
| Kuwait | 61.13(51.6to70.83) | 61(52.79to69.2) | 48.81(42.11to55.74) | 42.09(33.86to52.2) | 0.2 (-0.31 to 0.72) | -1.28 (-1.76 to -0.8) |
| Kyrgyzstan | 44.52(35.29to55.25) | 54.83(42.58to69.29) | 57.95(43.35to75.11) | 50.2(36.2to65.03) | 1.56 (1.2 to 1.93) | 0.35 (-0.02 to 0.73) |
| Lao People's Democratic Republic | 85.81(67.9to107.19) | 86.13(71.61to105.2) | 81.71(67.41to98.1) | 80.49(65.23to96.84) | -0.22 (-0.29 to -0.14) | -0.37 (-0.43 to -0.31) |
| Latvia | 47.34(32.5to65.29) | 44.81(31.34to60.34) | 41.23(29.79to54.44) | 37.55(27.13to49.86) | -1 (-1.42 to -0.58) | -1.11 (-1.32 to -0.91) |
| Lebanon | 84.83(67.7to103.94) | 77.44(62.4to93.29) | 81.56(63.71to98.85) | 78.43(59.27to96.78) | -0.25 (-0.44 to -0.07) | -0.05 (-0.16 to 0.06) |
| Lesotho | 40.52(34.08to48.47) | 51.1(42.58to61.26) | 71.12(57.47to85.68) | 75.43(57.34to94.95) | 3.37 (3.06 to 3.69) | 2.74 (2.48 to 3) |
| Liberia | 58.77(48.89to70.82) | 49.34(40.86to59.36) | 54.04(43.07to65.88) | 50.94(39.52to64.88) | -0.71 (-1.07 to -0.34) | -0.28 (-0.49 to -0.06) |
| Libya | 62.47(49.9to76.54) | 63.96(52.61to76.36) | 68.85(57.75to80.2) | 72.28(56.42to90.14) | 0.62 (0.32 to 0.92) | 0.71 (0.56 to 0.86) |
| Lithuania | 44.25(28.84to61.89) | 41.08(27.28to56.04) | 39.29(26.54to53.37) | 34.22(23.02to47.03) | -0.8 (-1.14 to -0.46) | -1.01 (-1.19 to -0.83) |
| Luxembourg | 37.58(30.25to44.93) | 32.02(26.54to37.72) | 24.84(20.81to29.12) | 19.99(16.24to23.98) | -1.96 (-2.08 to -1.84) | -2.24 (-2.34 to -2.13) |
| Madagascar | 39.76(32.44to47.36) | 41.04(33.81to49.29) | 40.21(32.26to49.59) | 39.83(30.87to51.18) | 0.06 (-0.09 to 0.21) | -0.09 (-0.18 to -0.01) |
| Malawi | 41.17(35.34to47.46) | 45.13(38.26to52.94) | 42.15(35.49to49.15) | 40.34(33.26to48.17) | 0.14 (-0.08 to 0.36) | -0.18 (-0.32 to -0.04) |
| Malaysia | 57.14(49.13to65.94) | 64.01(54.75to73.57) | 58.6(50.24to67.45) | 56.8(45.03to70.47) | 0.02 (-0.36 to 0.41) | -0.46 (-0.69 to -0.22) |
| Maldives | 101.56(86.73to118.33) | 84.05(71.46to97.59) | 63.29(54.71to72.44) | 59.77(48.24to71.33) | -2.8 (-3.03 to -2.58) | -2.31 (-2.51 to -2.11) |
| Mali | 53.31(43.77to64.87) | 46.76(38.4to57.71) | 49.15(40.93to59.32) | 48.78(39.45to60.33) | -0.48 (-0.7 to -0.26) | -0.18 (-0.32 to -0.05) |
| Malta | 46.77(38.1to55.96) | 39.73(32.71to47.44) | 30.87(25.15to37.01) | 25.37(20.41to30.31) | -1.91 (-2.08 to -1.74) | -2.11 (-2.22 to -2.01) |
| Marshall Islands | 85.05(70.45to104.97) | 97.72(79.05to123.32) | 108.35(85.72to138.32) | 109.01(83.48to141.68) | 1.41 (1.25 to 1.57) | 0.92 (0.77 to 1.08) |
| Mauritania | 67.74(57.67to79.12) | 55.75(45.58to66.31) | 51.78(42.76to62.14) | 48.28(38.25to58.39) | -1.37 (-1.52 to -1.22) | -1.14 (-1.24 to -1.04) |
| Mauritius | 100.14(86.96to114.76) | 104.85(91.8to118.01) | 109.4(100.44to118.04) | 102.79(84.58to124.07) | 0.25 (0.01 to 0.49) | -0.09 (-0.25 to 0.06) |
| Mexico | 55.05(48.88to61.62) | 71.64(65.25to77.73) | 86.46(79.5to92.95) | 89.35(76.31to103.63) | 2.58 (2.41 to 2.74) | 1.86 (1.64 to 2.07) |
| Micronesia (Federated States of) | 99.11(78.74to123.17) | 125.37(104.99to151.19) | 137.75(112.57to169.08) | 142.35(108.6to177.75) | 1.89 (1.68 to 2.1) | 1.2 (0.99 to 1.41) |
| Monaco | 22.94(16.68to29.78) | 19.94(14.8to25.42) | 18.72(14.51to23.03) | 16.89(12.93to21) | -1.15 (-1.26 to -1.03) | -0.99 (-1.06 to -0.92) |
| Mongolia | 87.2(67.88to109.3) | 103.64(80.11to129.48) | 77.88(59.34to97.52) | 70.24(51.81to90.84) | -0.84 (-1.43 to -0.24) | -1.42 (-1.75 to -1.09) |
| Montenegro | 43.89(36to52.29) | 50.05(41.76to58.71) | 55.87(46.27to65.82) | 53.99(42.98to66.02) | 1.41 (1.25 to 1.58) | 0.97 (0.81 to 1.13) |
| Morocco | 73.42(60.1to88.71) | 80.03(65.61to96.25) | 89.57(71.06to110.55) | 104.06(82.05to123.8) | 0.9 (0.78 to 1.03) | 1.24 (1.11 to 1.38) |
| Mozambique | 36.13(30.08to43.47) | 40.09(33.86to46.61) | 44.28(36.98to52.69) | 44.65(35.48to56.16) | 1.03 (0.97 to 1.09) | 0.93 (0.86 to 1.01) |
| Myanmar | 68.62(54.97to85.36) | 67.26(54.73to81.23) | 62.43(53.18to73.83) | 60.35(50.89to71.64) | -0.42 (-0.5 to -0.33) | -0.58 (-0.64 to -0.51) |
| Namibia | 48.78(39.32to61.28) | 57.25(46.32to71.43) | 49.19(39.35to61.74) | 50.13(39.41to64.06) | 0.36 (-0.08 to 0.81) | -0.17 (-0.44 to 0.09) |
| Nauru | 112.67(90.42to138.23) | 146.35(118.59to176.75) | 151.89(119.83to186.19) | 146.46(117.96to179.83) | 1.71 (1.4 to 2.02) | 0.8 (0.51 to 1.09) |
| Nepal | 32.73(25.87to41.31) | 32.93(27.39to39.67) | 36.42(30.07to42.88) | 45.12(34.69to55.32) | 0.25 (0.07 to 0.43) | 1.09 (0.84 to 1.34) |
| Netherlands | 29.41(23.56to35.55) | 25.68(21.18to30.39) | 19.17(16.1to22.33) | 18.59(15.69to21.52) | -2.16 (-2.41 to -1.91) | -1.86 (-2.03 to -1.69) |
| New Zealand | 36.86(29.65to44.48) | 32.03(25.9to38.54) | 25.91(21.38to30.74) | 24.29(20.09to28.68) | -1.58 (-1.74 to -1.43) | -1.65 (-1.75 to -1.54) |
| Nicaragua | 60.16(54.09to66.4) | 85.38(76.71to94.48) | 115.69(105.73to125.51) | 118.87(100.48to139.23) | 3.34 (2.99 to 3.68) | 2.51 (2.2 to 2.81) |
| Niger | 51.54(41.45to62.51) | 46.79(38.36to56.95) | 45.68(37.37to54.92) | 46.21(37.1to58.06) | -0.49 (-0.6 to -0.37) | -0.31 (-0.39 to -0.23) |
| Nigeria | 45.93(37.34to55.32) | 44.86(35.64to55.77) | 43.42(34.1to52.65) | 41.92(33.63to50.97) | -0.25 (-0.31 to -0.2) | -0.3 (-0.33 to -0.27) |
| Niue | 77.42(62.56to95.43) | 93.39(76.08to112.77) | 96.41(77.57to115.51) | 94.82(75.16to117.45) | 1.18 (0.97 to 1.39) | 0.56 (0.37 to 0.76) |
| North Macedonia | 50.33(41.16to60.24) | 64.79(53.24to77.55) | 66.92(53.85to80.02) | 62.42(47.68to79.59) | 1.52 (1.19 to 1.85) | 0.52 (0.21 to 0.83) |
| Northern Mariana Islands | 77.62(66.42to92.43) | 84.13(73.46to97.38) | 90.23(78.55to103.21) | 92.88(78.71to107.46) | 0.64 (0.55 to 0.74) | 0.79 (0.69 to 0.88) |
| Norway | 25.82(19.94to32.26) | 23.38(18.55to28.46) | 17.9(14.77to21.21) | 15.1(12.39to17.99) | -1.89 (-2.1 to -1.67) | -2.07 (-2.19 to -1.95) |
| Oman | 88.45(66.65to114.52) | 104.97(82.94to130.2) | 107.32(87.67to127.13) | 104.39(84.39to127.67) | 0.78 (0.58 to 0.99) | 0.72 (0.55 to 0.89) |
| Pakistan | 48.53(38.55to60.8) | 64.32(51.91to79.14) | 71.45(57.02to87.49) | 70.71(55.37to86.17) | 2.01 (1.77 to 2.26) | 1.24 (1 to 1.49) |
| Palau | 100.59(81.89to126.21) | 121.6(95.55to148.77) | 124.91(101.46to152.45) | 121.63(95.6to149.66) | 1.2 (0.98 to 1.42) | 0.61 (0.41 to 0.8) |
| Palestine | 104.67(82.38to130.72) | 102.35(86.94to119.75) | 101.02(87.3to114.17) | 91.74(75.67to109.66) | -0.2 (-0.25 to -0.15) | -0.51 (-0.61 to -0.41) |
| Panama | 37.32(31.36to43.34) | 39.5(34.92to44.35) | 43.13(38.04to47.7) | 42.04(32.83to52.92) | 0.81 (0.66 to 0.96) | 0.49 (0.37 to 0.61) |
| Papua New Guinea | 32.34(24.92to42.1) | 36.39(27.28to47.4) | 40.23(30.29to52.9) | 41.48(31.07to54.88) | 1.2 (1.13 to 1.26) | 0.9 (0.81 to 0.99) |
| Paraguay | 32.92(27.86to38.48) | 38.25(33.7to43) | 49.18(43.45to55.18) | 52.7(40.97to66.96) | 1.95 (1.75 to 2.15) | 2 (1.87 to 2.13) |
| Peru | 36.37(30.72to42.25) | 37.21(31.52to43.64) | 38.38(31.9to44.87) | 33.35(24.88to43.03) | -0.11 (-0.39 to 0.18) | -0.23 (-0.4 to -0.05) |
| Philippines | 56.76(50.21to63.96) | 61.55(54.03to70.03) | 76.94(67.52to87.06) | 78.25(64.6to93.38) | 1.9 (1.72 to 2.09) | 1.61 (1.47 to 1.76) |
| Poland | 50.02(38.52to62.48) | 43.83(34.76to53.78) | 32.79(25.98to39.83) | 26.82(20.49to33.74) | -2.59 (-2.91 to -2.27) | -2.83 (-3.02 to -2.64) |
| Portugal | 38.7(31.54to45.57) | 33.62(28.26to38.64) | 28.19(24.6to31.65) | 22.62(19.21to25.69) | -1.37 (-1.55 to -1.19) | -2 (-2.2 to -1.81) |
| Puerto Rico | 51.89(44.98to58.93) | 41.59(36.44to46.98) | 39.46(35.29to43.67) | 39.31(30.52to49.86) | -1.16 (-1.4 to -0.92) | -0.77 (-0.93 to -0.6) |
| Qatar | 124.63(99.39to155.82) | 133.62(108.73to161.95) | 129.83(107to155.05) | 113.55(88.65to142.05) | 0.38 (-0.09 to 0.85) | -0.28 (-0.58 to 0.01) |
| Republic of Korea | 45.03(37.63to52.88) | 28.75(24.24to33.02) | 20.44(17.16to23.76) | 17.34(14.51to20.37) | -3.78 (-3.97 to -3.59) | -3.32 (-3.5 to -3.14) |
| Republic of Moldova | 48.99(30.34to70.13) | 52.44(33.38to74.4) | 49.15(32.74to68.28) | 38.88(26.38to52.5) | -0.68 (-1.14 to -0.22) | -1.25 (-1.53 to -0.98) |
| Romania | 46.7(36.25to58.4) | 46.27(35.97to57.17) | 40.18(31.68to48.96) | 37.99(28.86to48.87) | -1.09 (-1.44 to -0.73) | -1.18 (-1.37 to -1) |
| Russian Federation | 56.04(41.27to73.24) | 71.3(53.37to91.85) | 59.28(44.43to76.07) | 47.69(34.99to61.79) | 0.15 (-0.5 to 0.8) | -1.06 (-1.51 to -0.61) |
| Rwanda | 50.5(42.05to59.77) | 47.18(39.59to54.84) | 36.48(30.1to42.94) | 38.38(30.07to46.81) | -2.19 (-2.57 to -1.82) | -1.6 (-1.86 to -1.33) |
| Saint Kitts and Nevis | 103.25(90.56to116.82) | 85.54(75.22to96.49) | 76.49(67.59to85.82) | 84.14(70.89to98.31) | -0.94 (-1.18 to -0.71) | -0.53 (-0.71 to -0.35) |
| Saint Lucia | 61.53(54.26to69.39) | 49.23(44.09to54.67) | 45.53(40.92to50.19) | 52.69(44.52to61.55) | -1.47 (-1.69 to -1.25) | -0.7 (-0.98 to -0.42) |
| Saint Vincent and the Grenadines | 55.88(47.56to65.07) | 54.08(47.07to62.12) | 57.32(50to64.7) | 63.86(54.55to74.52) | -0.15 (-0.37 to 0.07) | 0.59 (0.36 to 0.82) |
| Samoa | 78.79(64.66to96.51) | 89.18(72.5to109.77) | 92.16(75.49to110.7) | 91.48(74.73to113.23) | 0.87 (0.73 to 1.01) | 0.45 (0.31 to 0.58) |
| San Marino | 15.47(11.99to19.62) | 12.84(9.79to16.24) | 12.92(8.93to17.6) | 12.49(8.37to17.18) | -0.8 (-1.05 to -0.56) | -0.52 (-0.66 to -0.38) |
| Sao Tome and Principe | 61.28(50.75to69.9) | 71.5(61.39to82.42) | 75.59(63.83to86.13) | 78.65(64.08to89.76) | 1.04 (0.86 to 1.21) | 0.82 (0.72 to 0.92) |
| Saudi Arabia | 96.94(78.7to116.07) | 116.28(102.07to131.45) | 128.93(112.26to146.11) | 112.81(92.67to135.07) | 1.43 (1.33 to 1.53) | 0.6 (0.36 to 0.85) |
| Senegal | 57.98(46.83to70.82) | 50.92(41.54to64.29) | 51.85(42.72to61.63) | 52.16(42.05to64.28) | -0.31 (-0.55 to -0.07) | -0.28 (-0.39 to -0.16) |
| Serbia | 52.82(42.58to64.41) | 60.43(48.88to72.81) | 59.43(48.83to70.48) | 55.96(42.82to71.27) | 0.6 (0.31 to 0.89) | 0.06 (-0.15 to 0.26) |
| Seychelles | 59.91(51.86to69.01) | 71.45(63.24to79.97) | 71.74(63.63to79.85) | 71.15(61.46to80.72) | 0.95 (0.66 to 1.24) | 0.31 (0.09 to 0.53) |
| Sierra Leone | 50.24(40.87to60.23) | 46.72(38.45to56.03) | 50.63(41.53to60.28) | 48.54(38.65to60.82) | 0.23 (0.03 to 0.43) | 0.12 (0.01 to 0.23) |
| Singapore | 45.69(37.91to53.5) | 31.22(25.18to37.65) | 24.67(20.47to28.93) | 20.69(17.19to24.11) | -3.06 (-3.24 to -2.89) | -2.58 (-2.74 to -2.42) |
| Slovakia | 54.76(42.1to69.03) | 50.03(38.4to62.08) | 43.7(33.56to54.44) | 38.86(28.1to51.08) | -0.8 (-0.95 to -0.65) | -1.22 (-1.36 to -1.08) |
| Slovenia | 26.86(19.58to36.77) | 21.55(16.81to26.68) | 17.1(13.76to20.88) | 14.96(11.09to19.77) | -2.45 (-2.57 to -2.32) | -2.23 (-2.33 to -2.13) |
| Solomon Islands | 110.26(82.91to144.17) | 121.23(94.97to152.77) | 107.49(83.79to135.54) | 107.32(82.05to135.21) | -0.28 (-0.57 to 0) | -0.24 (-0.39 to -0.09) |
| Somalia | 47.82(36.27to59.33) | 45.49(33.64to58.5) | 48.03(36.35to61.06) | 47.21(35.89to60.9) | 0.09 (-0.04 to 0.22) | 0.13 (0.07 to 0.2) |
| South Africa | 35.86(31.74to40.21) | 53.76(48.78to59.19) | 54.08(49.02to59.13) | 48.14(43.12to53.03) | 2.05 (1.47 to 2.63) | 1.06 (0.66 to 1.46) |
| South Sudan | 40.24(30.9to50.85) | 39.04(29.6to49.46) | 39.34(29.61to49.85) | 39.14(29.78to49.48) | -0.17 (-0.26 to -0.09) | -0.07 (-0.12 to -0.02) |
| Spain | 33.52(28.37to39.15) | 26.28(21.9to30.64) | 20.83(17.31to23.99) | 18.29(15.32to21.13) | -2.1 (-2.21 to -1.98) | -2.12 (-2.19 to -2.04) |
| Sri Lanka | 55.24(46.37to65.21) | 59.55(51.57to68.06) | 56.31(48.73to64.62) | 48.48(35.87to63.5) | 0.51 (0.25 to 0.78) | -0.2 (-0.45 to 0.05) |
| Sudan | 80.4(64.12to98.48) | 80.81(63.32to100.07) | 81.58(64.45to101.41) | 88.5(70to111.64) | 0.07 (0.05 to 0.08) | 0.26 (0.19 to 0.33) |
| Suriname | 56.67(49.34to64.42) | 62.55(56.31to69.04) | 63.36(57.42to68.74) | 70.45(59.26to82.93) | 0.76 (0.18 to 1.33) | 0.73 (0.44 to 1.02) |
| Sweden | 29.9(21.9to38.57) | 24.28(18.42to30.67) | 20.01(15.64to24.42) | 18.47(14.82to22.45) | -2.03 (-2.08 to -1.98) | -1.72 (-1.81 to -1.62) |
| Switzerland | 26.14(20.34to32.36) | 23.61(18.48to29.21) | 20.69(16.57to24.8) | 18.9(15.29to22.59) | -1.2 (-1.29 to -1.11) | -1.12 (-1.18 to -1.07) |
| Syrian Arab Republic | 100.32(79.56to124.43) | 117.27(96.07to140.72) | 107.15(83.49to133.87) | 109.36(82.2to141.73) | -0.14 (-0.5 to 0.23) | -0.09 (-0.27 to 0.09) |
| Taiwan (Province of China) | 49.66(44.6to54.78) | 37.69(33.72to41.64) | 30.57(27.16to33.75) | 30.77(24.24to38.67) | -2.36 (-2.49 to -2.23) | -1.77 (-1.96 to -1.58) |
| Tajikistan | 30.16(20.78to41.09) | 41.55(27.85to57.14) | 56.46(39.21to77.03) | 65.25(44.61to88.24) | 2.89 (2.63 to 3.14) | 2.92 (2.75 to 3.09) |
| Thailand | 50.26(43.26to58.11) | 51.95(43.88to59.89) | 43.93(37.94to50.13) | 41.8(31.9to53.55) | -0.93 (-1.14 to -0.71) | -0.99 (-1.11 to -0.86) |
| Timor-Leste | 53.04(42.75to65.57) | 52.65(41.54to65.17) | 57.09(45.59to69.19) | 66.67(52.01to80.17) | 0.23 (0.11 to 0.36) | 0.87 (0.69 to 1.05) |
| Togo | 52.75(43.85to63.55) | 51.19(42.57to61.58) | 53.05(43.93to64.2) | 50.96(41.21to63.24) | 0.01 (-0.07 to 0.08) | -0.07 (-0.11 to -0.02) |
| Tokelau | 66.96(52.81to83.99) | 77.37(61.39to97.07) | 80.78(64.44to102.37) | 80.1(62.43to103.24) | 1.1 (0.93 to 1.26) | 0.6 (0.44 to 0.75) |
| Tonga | 51.31(42.32to62.55) | 67.01(55.84to80.74) | 67.11(55.2to81.87) | 68.47(55.69to85.47) | 1.68 (1.26 to 2.11) | 0.97 (0.69 to 1.25) |
| Trinidad and Tobago | 55.49(46.91to63.98) | 59.91(52.09to68.55) | 58.42(52.46to64.78) | 58.29(43.95to75.46) | 0.21 (-0.02 to 0.45) | 0.05 (-0.07 to 0.18) |
| Tunisia | 64.48(52.66to77.83) | 73.06(57.57to90.68) | 74.48(55.95to97.45) | 72.31(53.24to93.25) | 0.64 (0.5 to 0.78) | 0.33 (0.22 to 0.44) |
| Turkey | 67.58(55.33to84.26) | 54.11(44.49to65.93) | 57.46(47.7to68.23) | 54.27(42.57to68.04) | -1.11 (-1.41 to -0.81) | -0.54 (-0.75 to -0.32) |
| Turkmenistan | 59.52(43.47to77.33) | 71.3(52.45to91.26) | 65.94(50.9to82.81) | 71.22(51.98to93.6) | 0.53 (-0.04 to 1.09) | -0.01 (-0.32 to 0.3) |
| Tuvalu | 81.89(65.61to102.64) | 94.6(77.43to115.31) | 98.94(77.62to124.92) | 100.35(77.19to132.07) | 1.05 (0.91 to 1.19) | 0.67 (0.55 to 0.8) |
| Uganda | 36.84(30.12to44.76) | 42.08(34.67to51.76) | 38.66(31.69to46.6) | 38.61(31.42to46.84) | 0.21 (-0.05 to 0.47) | -0.09 (-0.23 to 0.06) |
| Ukraine | 46.56(30.55to64.65) | 59.42(37.35to83.55) | 55.86(35.53to79.02) | 57.79(37.96to81.27) | 0.78 (0.38 to 1.18) | 0.19 (-0.08 to 0.46) |
| United Arab Emirates | 127.7(105.9to150.58) | 155.42(127.35to184.56) | 146.11(118.9to175.34) | 98.48(74.77to128.41) | 1.01 (0.56 to 1.46) | -1.05 (-1.67 to -0.42) |
| United Kingdom | 30.06(23.5to37.44) | 22.86(17.93to28.42) | 15.68(12.63to18.84) | 14.9(12.21to17.8) | -3.35 (-3.52 to -3.18) | -2.86 (-3.07 to -2.66) |
| United Republic of Tanzania | 39.39(33.35to46.2) | 38.91(32.96to45.63) | 41(34.93to47.45) | 42.52(35.94to50.04) | 0.14 (0.02 to 0.26) | 0.33 (0.26 to 0.41) |
| United States of America | 40.97(33.08to49.35) | 39.77(32.97to46.88) | 34.2(29.09to39.19) | 34.82(29.97to39.8) | -0.72 (-0.9 to -0.53) | -0.67 (-0.77 to -0.56) |
| United States Virgin Islands | 50.07(40.19to60.38) | 53.34(44.62to63.52) | 65.37(55.95to74.76) | 63.23(52.8to73.82) | 1.47 (1.18 to 1.75) | 1.19 (1.02 to 1.37) |
| Uruguay | 35.95(30.11to42.22) | 30.1(25.85to34.32) | 27.23(23.69to30.73) | 27.53(23.86to31.02) | -1.09 (-1.29 to -0.89) | -0.85 (-0.97 to -0.73) |
| Uzbekistan | 56.4(43.71to71.73) | 103.35(79.55to130.9) | 138.2(103.07to176.04) | 124.73(90.9to162.82) | 4.59 (4.07 to 5.11) | 2.78 (2.2 to 3.36) |
| Vanuatu | 68.65(50.9to90.38) | 82.04(63.42to106.35) | 93.43(72.01to120.02) | 100.8(78.44to131.11) | 1.26 (1.08 to 1.44) | 1.25 (1.16 to 1.35) |
| Venezuela (Bolivarian Republic of) | 48.26(40.3to57.11) | 58.64(51.52to66.22) | 61.9(55.01to69.2) | 67.4(51.36to86.46) | 1.47 (1.25 to 1.69) | 0.85 (0.65 to 1.05) |
| Viet Nam | 53.69(44.27to64.26) | 46.63(39.84to54.16) | 49.43(42.42to57.67) | 54.28(43.77to64.82) | -0.48 (-0.72 to -0.24) | 0.16 (-0.05 to 0.37) |
| Yemen | 75.13(58.24to97.13) | 74.94(57.45to96.04) | 76.19(59.43to94.96) | 83.72(66.09to106.51) | 0.08 (0.03 to 0.12) | 0.34 (0.26 to 0.43) |
| Zambia | 47.25(39.68to55.21) | 53.36(45.22to61.59) | 50.51(42.88to58.79) | 48.69(39.71to59.69) | 0.26 (0.04 to 0.48) | -0.11 (-0.26 to 0.03) |
| Zimbabwe | 43.92(35.37to57.11) | 51.9(41.36to67.6) | 61.7(48.86to81.16) | 61.17(46.83to81.93) | 2.11 (1.87 to 2.36) | 1.46 (1.24 to 1.67) |

ASMR, age-standard morality rate; EAPC, estimated annual percentage change.
