# Supplementary material for: Global, regional, and national burden of kidney dysfunction from 1990 to 2019: a systematic analysis from the global burden of disease study 2019
Source: BMC Public Health. 2023 Jun 23;23:1218. doi: 10.1186/s12889-023-16130-8 (PMC10288715; doi:10.1186/s12889-023-16130-8)
Supplement: Supplementary file 10 — Additional file 10: Table 5S. Age-standardized DALYs of kidney dysfunction for both sexes combined in 1990,2000,2010, and 2019, and EAPC of ASDR from 1990 to 2019 and 1990 to 2010 in 204 countries and territories [file 12889_2023_16130_MOESM10_ESM.docx]

Table 5S. Age-standardized DALYs of kidney dysfunction for both sexes combined in 1990,2000,2010, and 2019, and EAPC of ASDR from 1990 to 2019 and 1990 to 2010 in 204 countries and territories

| Location | DALYs 1990 | DALYs 2000 | DALYs 2010 | DALYs 2019 | EAPC 1990-2010 | EAPC 1990-2019 |
| --- | --- | --- | --- | --- | --- | --- |
| Afghanistan | 2744.17(2165.13to3404.66) | 2777.98(2171.59to3520.85) | 2629.08(2093.43to3308.58) | 2606.38(2066.2to3288.05) | -0.05 (-0.17 to 0.06) | -0.17 (-0.24 to -0.1) |
| Albania | 768.38(667.92to876.29) | 708.24(612.21to810.8) | 672(549.37to807.91) | 695.51(533.76to883.52) | -0.34 (-0.64 to -0.04) | -0.19 (-0.34 to -0.03) |
| Algeria | 1916.97(1542.45to2362.89) | 1738.67(1390.01to2149.91) | 1610.21(1315.98to1937.39) | 1593.8(1287.15to1944.35) | -0.83 (-0.86 to -0.8) | -0.65 (-0.71 to -0.59) |
| American Samoa | 1822.12(1582.82to2083.07) | 2124.65(1890.18to2389.24) | 2275.13(2020.65to2546.19) | 2424.75(2062.04to2867.07) | 1.18 (1.04 to 1.31) | 1.02 (0.93 to 1.1) |
| Andorra | 389.99(313.58to504.81) | 334.89(273.23to418.78) | 298.87(242.78to366.82) | 295.03(232.72to370.69) | -1.45 (-1.52 to -1.38) | -1.03 (-1.15 to -0.9) |
| Angola | 1063.89(832.89to1319.36) | 1044.27(823.82to1252.27) | 975.29(770.21to1177.23) | 933.16(731.6to1171.53) | -0.49 (-0.57 to -0.41) | -0.55 (-0.6 to -0.5) |
| Antigua and Barbuda | 1232.67(1102.61to1371.89) | 1246.79(1122.34to1381.2) | 1357.7(1228.24to1500.89) | 1411.24(1220.46to1626.42) | 0.35 (0.1 to 0.6) | 0.54 (0.4 to 0.67) |
| Argentina | 1003.02(896.27to1115.49) | 991.16(909.29to1076.79) | 883.66(813.13to959.04) | 845.72(775.25to918.47) | -0.42 (-0.64 to -0.19) | -0.64 (-0.77 to -0.51) |
| Armenia | 772.65(560.96to1002) | 864.13(652.38to1096.27) | 893.59(710.67to1090.11) | 895.55(705.19to1117.27) | 0.17 (-0.12 to 0.46) | 0.09 (-0.05 to 0.24) |
| Australia | 606.07(505.08to713.28) | 460.24(389.91to536.09) | 365.54(315.75to418.18) | 354.71(306.67to404.95) | -2.54 (-2.62 to -2.46) | -1.99 (-2.18 to -1.81) |
| Austria | 523.23(434.54to619.31) | 504.08(421.2to594.22) | 472.29(408.04to540.17) | 437.15(376.99to499.84) | -0.51 (-0.6 to -0.43) | -0.61 (-0.67 to -0.55) |
| Azerbaijan | 1187.71(947.95to1471.63) | 1406.94(1102.82to1754.24) | 1673.12(1364.64to2011.83) | 1678.1(1313.66to2064.03) | 1.28 (0.97 to 1.59) | 1.08 (0.91 to 1.25) |
| Bahamas | 1230.1(1087.41to1379.52) | 1255.08(1123.89to1403.54) | 1307.87(1183.4to1443.81) | 1333.38(1112.5to1612.06) | 0.43 (0.31 to 0.55) | 0.4 (0.34 to 0.46) |
| Bahrain | 2101.5(1722.38to2506.16) | 1850.23(1570.47to2150.91) | 1710.07(1486.81to1965.43) | 1378.56(1111.76to1668.26) | -0.75 (-1.17 to -0.33) | -1.63 (-1.96 to -1.31) |
| Bangladesh | 831.41(647.04to1006.61) | 787.57(656.35to925.21) | 872.7(752.09to1013.92) | 734.71(587.01to921.56) | 0.86 (0.56 to 1.16) | 0.05 (-0.21 to 0.32) |
| Barbados | 957.42(853.45to1067.48) | 1012.1(915.98to1120.41) | 1010.65(913.78to1115.87) | 1058.05(888.5to1249.42) | 0.29 (0.15 to 0.43) | 0.22 (0.15 to 0.29) |
| Belarus | 846.36(606.18to1108.46) | 1043.04(740.68to1372.08) | 1107.87(802.42to1446.09) | 948.69(648.66to1301.31) | 1.16 (0.75 to 1.57) | 0.11 (-0.25 to 0.46) |
| Belgium | 538.02(455.22to624.8) | 441.5(376.91to510.59) | 367.12(320.09to418.61) | 327.07(282.84to372.56) | -1.8 (-1.87 to -1.74) | -1.79 (-1.84 to -1.74) |
| Belize | 1077.44(952.34to1216.92) | 1615.57(1465.41to1775.19) | 1529.79(1404.1to1663.89) | 1568.53(1363.94to1799.51) | 2.04 (1.45 to 2.63) | 1.22 (0.86 to 1.58) |
| Benin | 1250.08(1078.39to1432.9) | 1235.89(1029.03to1466) | 1261.38(1020.08to1559.87) | 1189.01(946.05to1506.64) | 0.01 (-0.03 to 0.06) | -0.08 (-0.13 to -0.03) |
| Bermuda | 1073.24(911.86to1247.47) | 839.51(730.59to967.16) | 717.26(627.31to820.99) | 708.64(600.57to838.69) | -1.94 (-2.1 to -1.79) | -1.37 (-1.55 to -1.19) |
| Bhutan | 1017.95(742.46to1314.48) | 1070.32(861.38to1312.74) | 1117.35(894.03to1357.34) | 1148.67(884.03to1425.16) | 0.5 (0.45 to 0.56) | 0.44 (0.41 to 0.48) |
| Bolivia (Plurinational State of) | 1379.6(1177.14to1668.01) | 1459.69(1263.78to1743.06) | 1483.77(1229.86to1792.89) | 1544.16(1239.65to1930.52) | 0.34 (0.27 to 0.41) | 0.4 (0.36 to 0.43) |
| Bosnia and Herzegovina | 798.89(664.98to949.42) | 795.28(665.92to946.2) | 806.9(693.14to928.08) | 795.41(630.35to998.96) | -0.08 (-0.31 to 0.14) | -0.21 (-0.32 to -0.09) |
| Botswana | 1148.4(883.32to1487.29) | 1630.18(1148.96to2221.96) | 1464.96(1076.98to1892.59) | 1478.5(1109.2to1920.94) | 1.1 (0.47 to 1.74) | 0.44 (0.09 to 0.79) |
| Brazil | 1074.16(968.82to1189.98) | 961.82(877.09to1057.12) | 818.98(745.59to900.86) | 777.53(705.57to856.54) | -1.34 (-1.44 to -1.24) | -1.22 (-1.29 to -1.14) |
| Brunei Darussalam | 1680.97(1470.76to1929.86) | 1360.26(1188.45to1547.85) | 1302.46(1157.3to1448.58) | 1247.78(1091.32to1413.54) | -1.04 (-1.32 to -0.76) | -0.73 (-0.89 to -0.56) |
| Bulgaria | 1117.56(894.52to1356.63) | 1350.68(1103.03to1608.53) | 1211.55(1037.69to1401.6) | 1208.12(960.61to1507.49) | 0.13 (-0.33 to 0.6) | -0.28 (-0.53 to -0.02) |
| Burkina Faso | 1008.48(833.05to1196.81) | 1049.73(877.05to1209.7) | 1065.5(885.58to1240.4) | 1151.01(949.9to1375.76) | 0.36 (0.29 to 0.42) | 0.49 (0.43 to 0.55) |
| Burundi | 1240.83(1007.97to1512.93) | 1160.66(941.53to1412.25) | 997.93(810.84to1198.98) | 948.23(750.53to1190.53) | -1.32 (-1.49 to -1.16) | -1.19 (-1.28 to -1.09) |
| Cabo Verde | 684.83(598.48to775.16) | 766.24(637to905.51) | 743.56(666.4to828.38) | 930.65(795.65to1076.08) | 0.39 (0.14 to 0.63) | 0.54 (0.34 to 0.74) |
| Cambodia | 1347.16(1130.46to1575.77) | 1257.58(1065.77to1453.97) | 1150.39(978.33to1353.83) | 1161.83(945.99to1382.06) | -0.88 (-0.95 to -0.82) | -0.65 (-0.73 to -0.57) |
| Cameroon | 1478.19(1225.94to1771.38) | 1582.5(1284.7to1894.43) | 1626.1(1297.82to1995.11) | 1517.14(1176.83to1963.92) | 0.53 (0.45 to 0.61) | 0.19 (0.07 to 0.31) |
| Canada | 566.43(469.78to662.79) | 471.55(399.62to547.44) | 384.79(335.54to439.69) | 365.87(316.82to419.01) | -1.92 (-1.97 to -1.86) | -1.64 (-1.74 to -1.54) |
| Central African Republic | 1292.49(1043.15to1557.39) | 1331.13(1032.66to1665.36) | 1337.68(1024.23to1703.66) | 1286.14(972.67to1673.84) | 0.18 (0.11 to 0.25) | 0.04 (-0.01 to 0.1) |
| Chad | 1114.87(904.33to1445.72) | 1163.59(944.34to1470.65) | 1213.17(988.65to1480.6) | 1163.23(919.81to1438.66) | 0.49 (0.43 to 0.55) | 0.23 (0.15 to 0.32) |
| Chile | 718.3(634.56to810.5) | 687.58(626.88to752.94) | 678.19(617.26to741.01) | 622.34(566.01to683.83) | 0.13 (-0.08 to 0.33) | -0.26 (-0.4 to -0.12) |
| China | 862.82(746.82to994.89) | 781.02(689.83to882.27) | 821.93(710.54to940.93) | 708.75(593.34to832.28) | 0.11 (-0.18 to 0.39) | -0.18 (-0.35 to 0) |
| Colombia | 1098.36(972.58to1234.62) | 939.93(838.34to1052.28) | 820.68(727.37to920.21) | 771.77(620.02to955.64) | -1.7 (-1.87 to -1.53) | -1.41 (-1.54 to -1.28) |
| Comoros | 1026.25(622.28to1295.74) | 1022.13(837.01to1214.61) | 882.63(740.12to1054.02) | 935.25(749.36to1147.28) | -0.89 (-1.12 to -0.65) | -0.53 (-0.7 to -0.35) |
| Congo | 1429.23(1112.35to1784.22) | 1356.84(1069.06to1649.45) | 1196.8(909.28to1492.54) | 1154.76(877.88to1454.16) | -1.02 (-1.24 to -0.79) | -0.9 (-1.02 to -0.78) |
| Cook Islands | 1296.72(1098.87to1527.64) | 1266.36(1086.67to1474.61) | 1300.14(1128.08to1499.16) | 1391.35(1150.68to1668.2) | -0.02 (-0.08 to 0.05) | 0.33 (0.23 to 0.43) |
| Costa Rica | 1097.91(965.94to1241.8) | 1170.05(1049.07to1293.44) | 1150.03(1040.11to1260.34) | 1104.38(893.71to1361.72) | -0.05 (-0.36 to 0.26) | -0.15 (-0.31 to 0.01) |
| Croatia | 865.17(703.25to1046.35) | 835.23(699.77to978.36) | 719.85(605.1to845.22) | 628.77(500.46to786.53) | -0.7 (-0.91 to -0.48) | -1.17 (-1.34 to -1) |
| Cuba | 757.79(637.27to893.42) | 757.53(658.73to874.07) | 798.87(709.99to895.6) | 828.29(677.61to1006.81) | 0.25 (0.15 to 0.34) | 0.28 (0.2 to 0.36) |
| Cyprus | 1059.46(917.99to1227.76) | 918.7(797.95to1054.19) | 682.83(595.81to779.04) | 549.75(462.37to638.94) | -2.53 (-2.77 to -2.29) | -2.69 (-2.82 to -2.57) |
| Czechia | 997.1(805.13to1209.1) | 772.16(636.33to925.09) | 583.92(477.45to699.39) | 514.42(406.65to642.52) | -2.56 (-2.67 to -2.46) | -2.42 (-2.51 to -2.33) |
| C么te d'Ivoire | 1396.26(1163.29to1649.12) | 1466.49(1218.61to1733.05) | 1366.68(1114.01to1650.24) | 1253.87(985.99to1542.35) | -0.14 (-0.3 to 0.03) | -0.44 (-0.56 to -0.32) |
| Democratic People's Republic of Korea | 1105.06(877.67to1339.13) | 1155.23(934.29to1382.85) | 1200.37(995.33to1437.5) | 1118.24(915.48to1368.4) | 0.45 (0.43 to 0.47) | 0.13 (0.03 to 0.23) |
| Democratic Republic of the Congo | 1096.51(908.11to1306.38) | 1041.44(879.38to1214.81) | 958.38(793.51to1137.4) | 935.15(753.6to1149.29) | -0.69 (-0.75 to -0.63) | -0.63 (-0.67 to -0.59) |
| Denmark | 557.01(448.69to675.49) | 477.85(408.42to558.21) | 378.17(331.22to429.17) | 329.98(287.71to378.4) | -2.12 (-2.3 to -1.93) | -2.12 (-2.23 to -2.02) |
| Djibouti | 876.3(682.91to1106.88) | 951.61(722.43to1227) | 1012.1(756.69to1314.24) | 1015.24(792.28to1320.99) | 0.71 (0.63 to 0.78) | 0.48 (0.4 to 0.56) |
| Dominica | 1471.81(1288.71to1667.6) | 1547.74(1347.67to1757.94) | 1645.64(1454.34to1855.46) | 1716.55(1435.83to2068.56) | 0.69 (0.56 to 0.82) | 0.69 (0.62 to 0.76) |
| Dominican Republic | 822.3(710.96to948.4) | 909.67(796.71to1028.82) | 1194.38(978.22to1455.55) | 1350.28(1052.07to1701.64) | 2.49 (2.18 to 2.8) | 2.36 (2.17 to 2.55) |
| Ecuador | 817.15(744.13to898.95) | 1183.75(1101.91to1269.35) | 1507.21(1368.2to1658.94) | 1386.5(1130.56to1726.24) | 3.54 (3.21 to 3.88) | 1.99 (1.53 to 2.45) |
| Egypt | 2078.66(1728.48to2441.53) | 2139.2(1772.47to2500.66) | 2488.28(2066.52to2875.25) | 2552.23(1952.16to3196.45) | 1.03 (0.89 to 1.18) | 0.88 (0.79 to 0.97) |
| El Salvador | 1126.16(1006.16to1255.13) | 1783.25(1657.81to1925.05) | 2358.63(2204.28to2522.82) | 2338.08(1820.07to2973.48) | 4.33 (4 to 4.66) | 2.9 (2.45 to 3.34) |
| Equatorial Guinea | 1182.54(912.99to1492.03) | 1012.66(771.96to1299.61) | 975.31(697.5to1321.94) | 1008.72(757.85to1344.27) | -1.19 (-1.41 to -0.97) | -0.58 (-0.78 to -0.37) |
| Eritrea | 1008.8(754.19to1307.71) | 1073.79(817.48to1363.45) | 1055.62(796.13to1361.06) | 1054.6(799.36to1392.92) | 0.31 (0.18 to 0.45) | 0.07 (-0.02 to 0.16) |
| Estonia | 1017.13(751.04to1313.1) | 986.15(756.68to1251.71) | 664.52(528.59to826.21) | 625.74(476.87to810.3) | -2.29 (-2.8 to -1.78) | -2.35 (-2.61 to -2.08) |
| Eswatini | 1390.12(1141.61to1649.39) | 1851.21(1403.92to2367.15) | 2072.59(1546.27to2674.98) | 1842.76(1372to2375.18) | 2.82 (2.33 to 3.3) | 1.22 (0.73 to 1.72) |
| Ethiopia | 1390.26(1174.12to1612.93) | 1135.62(1000.34to1277.27) | 886.55(798.87to989.21) | 823.69(705.72to959.36) | -2.34 (-2.41 to -2.26) | -2.06 (-2.18 to -1.95) |
| Fiji | 2187.88(1758.61to2703.24) | 2726.23(2396.26to3108.81) | 2400.96(2115.17to2705.91) | 2404.1(1934.61to2984.57) | 0.33 (-0.04 to 0.71) | -0.01 (-0.22 to 0.19) |
| Finland | 497.82(386.13to615.02) | 393.73(312.4to479.79) | 333.64(271.08to398.77) | 292.09(237.89to350.42) | -1.81 (-1.93 to -1.69) | -1.85 (-1.93 to -1.78) |
| France | 351.92(301.65to405.36) | 309.39(265.3to355.4) | 250.71(216.66to286.54) | 229.64(197.45to265.23) | -1.6 (-1.74 to -1.45) | -1.6 (-1.69 to -1.52) |
| Gabon | 1260.89(1012.37to1548.87) | 1371.27(1051.07to1656.98) | 1365.25(1017.95to1676.57) | 1293.69(930.64to1626.22) | 0.63 (0.49 to 0.77) | 0.06 (-0.1 to 0.23) |
| Gambia | 1091.5(843.94to1373.04) | 1082.92(870.82to1325.71) | 1171.86(958.2to1398.95) | 1234.63(975.89to1523.57) | 0.55 (0.31 to 0.78) | 0.39 (0.24 to 0.55) |
| Georgia | 1248.83(937.65to1602.44) | 1220.02(947.26to1526.39) | 1212.62(1016.77to1442.52) | 1105.18(894.58to1345.4) | 0.07 (-0.23 to 0.38) | -0.62 (-0.88 to -0.36) |
| Germany | 646.58(537.2to770.58) | 519.81(438.87to601.91) | 431.99(374.26to489.87) | 429.97(376.41to487.56) | -2.14 (-2.25 to -2.04) | -1.48 (-1.68 to -1.28) |
| Ghana | 1072.61(858.06to1312.42) | 1104.83(901.4to1315.37) | 1240.48(1008.28to1461.92) | 1213.58(973.58to1488.24) | 0.91 (0.78 to 1.05) | 0.63 (0.53 to 0.74) |
| Greece | 813.24(710.17to920.06) | 698.09(608.35to792.19) | 527.9(454.95to604.36) | 527.55(462.01to596.34) | -2.07 (-2.23 to -1.91) | -1.84 (-1.99 to -1.68) |
| Greenland | 850.46(714.56to1015.59) | 790.68(673.33to930.86) | 634.6(552.69to726.73) | 582.08(483.51to691.1) | -1.86 (-2.16 to -1.55) | -1.62 (-1.79 to -1.46) |
| Grenada | 1704.23(1520.31to1905.81) | 1603.46(1457.06to1767.08) | 1806.91(1655.81to1980.03) | 1870.11(1671.3to2089.8) | 0.32 (0.06 to 0.57) | 0.55 (0.41 to 0.69) |
| Guam | 1275.98(1086.56to1491.98) | 1386.59(1195.45to1604.26) | 1453.95(1257.07to1671.36) | 1669.08(1388.64to1993.65) | 0.73 (0.47 to 0.98) | 1.02 (0.86 to 1.17) |
| Guatemala | 1411.56(1234.19to1605.44) | 1365.18(1202.03to1543.52) | 1830.54(1624.13to2062.41) | 1906.08(1545.39to2344.6) | 0.78 (0.24 to 1.33) | 1.46 (1.12 to 1.81) |
| Guinea | 1279.13(1054.49to1578.01) | 1191.65(997.01to1473.18) | 1296.96(1073.34to1554.9) | 1277.11(1009.19to1574.93) | 0.11 (-0.07 to 0.29) | 0.26 (0.16 to 0.37) |
| Guinea-Bissau | 1861.89(1516.04to2291.04) | 1715.65(1409.93to2037.47) | 1740.85(1459.7to2074.59) | 1600.91(1263.17to1967.53) | -0.28 (-0.41 to -0.15) | -0.38 (-0.45 to -0.31) |
| Guyana | 1978.39(1670.3to2302.26) | 1993.18(1721.74to2302.44) | 2276.27(1991.02to2586.15) | 2312.34(1827.87to2903.38) | 0.8 (0.63 to 0.97) | 0.88 (0.77 to 1) |
| Haiti | 1892.3(1566.15to2316.68) | 1648.3(1348.83to2070.5) | 1793.73(1424.35to2294.05) | 1794.3(1372.51to2339.15) | -0.19 (-0.47 to 0.08) | 0.15 (-0.02 to 0.31) |
| Honduras | 1241.31(1040.1to1565.65) | 1585.35(1232.13to2003.47) | 1872.74(1479.72to2310.88) | 2052.73(1701.62to2534.33) | 2.21 (2 to 2.42) | 1.99 (1.82 to 2.15) |
| Hungary | 855.1(691.6to1034.59) | 788.7(646.18to954.65) | 728.03(602.39to864.98) | 679.22(540.24to845.84) | -0.96 (-1.1 to -0.82) | -0.88 (-0.96 to -0.8) |
| Iceland | 417.87(334.18to509.56) | 332.46(270.64to397.59) | 261.49(217.69to311.33) | 233.51(194.35to277.95) | -2.42 (-2.52 to -2.32) | -2.3 (-2.4 to -2.2) |
| India | 1258.23(1072.72to1471.97) | 1349.07(1167.22to1534.54) | 1223.43(1065.99to1397.65) | 1126.04(932.11to1337.17) | -0.09 (-0.33 to 0.15) | -0.43 (-0.58 to -0.28) |
| Indonesia | 1343.07(1175.8to1526.39) | 1384.95(1209.35to1575.88) | 1465.44(1274.89to1671.99) | 1437.59(1216.89to1665.7) | 0.5 (0.45 to 0.54) | 0.34 (0.28 to 0.4) |
| Iran (Islamic Republic of) | 1596.69(1377.81to1837.63) | 1547.44(1332.83to1770.71) | 1181.66(1038.48to1339.45) | 1127.16(981.05to1282.74) | -1.44 (-1.74 to -1.14) | -1.56 (-1.74 to -1.38) |
| Iraq | 2300.7(1910.28to2783.37) | 2310.07(1799.09to2954.28) | 2337.75(1830.97to2859.31) | 2306.74(1839.22to2759.31) | 0.05 (0 to 0.11) | -0.1 (-0.16 to -0.05) |
| Ireland | 806.51(664.49to950.01) | 648.08(546.55to753.78) | 384.78(329.57to441.62) | 355.97(306to410.89) | -3.78 (-4.14 to -3.43) | -3.39 (-3.65 to -3.14) |
| Israel | 839.45(727.96to957.3) | 721.3(647.25to803.65) | 577.94(523.99to638.49) | 520.69(467.95to576.71) | -1.68 (-1.87 to -1.49) | -1.81 (-1.91 to -1.7) |
| Italy | 519.75(447.58to594.82) | 409.15(353.55to470.15) | 323.56(278.55to370.04) | 297.36(256.4to340.51) | -2.3 (-2.37 to -2.22) | -2.04 (-2.14 to -1.94) |
| Jamaica | 968.19(881.49to1065.89) | 1327.55(1226.19to1430.67) | 1126.87(1035.27to1224.94) | 1256.1(1029.26to1540.2) | 0.61 (-0.13 to 1.36) | 0.62 (0.25 to 0.99) |
| Japan | 581.06(508.62to657.96) | 482.34(427.37to542.13) | 406.22(355.48to457.65) | 348.29(302.51to395.54) | -1.85 (-1.92 to -1.79) | -1.76 (-1.81 to -1.72) |
| Jordan | 1771.48(1519.16to2067.8) | 1791.66(1515.49to2122.83) | 1529.17(1338.28to1728.51) | 1461.45(1236.28to1738.31) | -0.53 (-0.84 to -0.21) | -0.91 (-1.1 to -0.72) |
| Kazakhstan | 1098.48(883.21to1347.38) | 1562.8(1246.15to1927.99) | 1337.66(1082.62to1629.09) | 1133.15(919.45to1359.12) | 0.69 (-0.05 to 1.45) | -0.6 (-1.1 to -0.1) |
| Kenya | 698.02(597.98to812.73) | 767.32(660.22to876.78) | 853.15(751.71to974.93) | 845.28(724.69to980.89) | 1.22 (1.09 to 1.36) | 0.79 (0.65 to 0.93) |
| Kiribati | 2945.85(2380.75to3539.31) | 3421.46(2846.98to4066.16) | 3291.52(2642.09to3990.57) | 3217.31(2514.46to3996.41) | 0.67 (0.43 to 0.9) | 0.11 (-0.07 to 0.3) |
| Kuwait | 1276.51(1107.71to1452.82) | 1302.53(1153.41to1452.04) | 1040.08(917.47to1178.3) | 904.99(747.96to1103.65) | 0.19 (-0.3 to 0.67) | -1.15 (-1.59 to -0.72) |
| Kyrgyzstan | 1252.88(1075.59to1459.46) | 1448.92(1226.98to1690.57) | 1371.42(1130.08to1637.23) | 1143.69(908.31to1393.62) | 0.51 (0.11 to 0.91) | -0.59 (-0.95 to -0.24) |
| Lao People's Democratic Republic | 2323.36(1872.33to2826.9) | 2255.01(1897.03to2699.32) | 2081.62(1723.38to2506.06) | 1973.89(1574.99to2400.43) | -0.51 (-0.57 to -0.44) | -0.68 (-0.74 to -0.62) |
| Latvia | 942.73(687.82to1230.02) | 920.16(690.13to1180.94) | 853.27(663.89to1062.59) | 758.1(575.29to975.65) | -0.9 (-1.42 to -0.38) | -1.19 (-1.45 to -0.92) |
| Lebanon | 1666.35(1378.58to2017.06) | 1463.83(1221.91to1721.82) | 1534.78(1253.18to1800.14) | 1516.45(1193.63to1825.14) | -0.47 (-0.69 to -0.24) | -0.1 (-0.25 to 0.05) |
| Lesotho | 963.99(809.58to1148.65) | 1213.71(1019.33to1446.51) | 1697.06(1358.55to2047) | 1790.69(1349.8to2269.2) | 3.44 (3.11 to 3.77) | 2.77 (2.49 to 3.05) |
| Liberia | 1418.36(1170.5to1698.66) | 1132.11(924.58to1365.6) | 1222.65(970.67to1496.82) | 1154.67(880.41to1484.63) | -1.1 (-1.5 to -0.7) | -0.49 (-0.75 to -0.23) |
| Libya | 1338.59(1105.35to1599.19) | 1390.56(1175.35to1604.6) | 1460.61(1276.21to1659.08) | 1542.63(1231.06to1881.83) | 0.56 (0.32 to 0.81) | 0.63 (0.51 to 0.76) |
| Lithuania | 861.94(611.56to1142.54) | 814.25(603.27to1045) | 786.72(587.12to1002.15) | 671.57(498.71to887.03) | -0.65 (-1.09 to -0.21) | -1.03 (-1.27 to -0.79) |
| Luxembourg | 623.61(527.29to731.27) | 523.19(449.09to602.1) | 416.66(360.63to478.19) | 337.76(289.24to394.31) | -1.88 (-1.97 to -1.79) | -2.16 (-2.25 to -2.06) |
| Madagascar | 1003.05(839to1179.22) | 984(818.77to1162.42) | 958.57(761.23to1172.12) | 937.9(741.12to1188.05) | -0.23 (-0.32 to -0.14) | -0.28 (-0.33 to -0.24) |
| Malawi | 1005.48(851.93to1164.23) | 1097.75(923.51to1279.37) | 999.39(843.64to1159.57) | 936.76(770.84to1115.21) | -0.04 (-0.29 to 0.21) | -0.38 (-0.53 to -0.23) |
| Malaysia | 1393.8(1226.83to1577.2) | 1459.49(1281.24to1653.66) | 1357.28(1184.31to1536.46) | 1341.35(1087.67to1625.78) | -0.19 (-0.43 to 0.04) | -0.37 (-0.5 to -0.24) |
| Maldives | 2345.65(2031.55to2715.62) | 1809.24(1571.23to2081.74) | 1329.42(1176.66to1494.55) | 1253.62(1044.39to1473.38) | -3.28 (-3.49 to -3.06) | -2.6 (-2.83 to -2.36) |
| Mali | 1312.97(1084.07to1572.19) | 1111.96(918.53to1366.25) | 1137.33(937.38to1362.64) | 1135.54(896.45to1422.83) | -0.81 (-1.03 to -0.58) | -0.41 (-0.57 to -0.26) |
| Malta | 775.03(647.66to905.3) | 659.9(567.85to767.6) | 522.9(447.63to606.63) | 439.81(375.39to513) | -1.8 (-1.95 to -1.65) | -1.96 (-2.04 to -1.87) |
| Marshall Islands | 2255.47(1900.91to2728.94) | 2643.85(2181.44to3287.17) | 2894.28(2311.01to3683.46) | 2882.71(2213.25to3743.47) | 1.42 (1.27 to 1.57) | 0.87 (0.71 to 1.04) |
| Mauritania | 1579.4(1337.45to1845.83) | 1258.11(1032.68to1486.28) | 1153.81(959.31to1381.04) | 1047.88(805.3to1300.24) | -1.54 (-1.72 to -1.36) | -1.33 (-1.43 to -1.22) |
| Mauritius | 2456.81(2177.13to2763.79) | 2567.53(2308.44to2848.03) | 2789.79(2602.63to3001.28) | 2622.81(2174.74to3152.94) | 0.45 (0.23 to 0.67) | 0.11 (-0.03 to 0.26) |
| Mexico | 1285.86(1168.7to1415.79) | 1752.11(1631.32to1884.53) | 2150.09(2013.76to2305.42) | 2231.22(1927.45to2550.18) | 2.95 (2.77 to 3.14) | 2.11 (1.86 to 2.36) |
| Micronesia (Federated States of) | 2724.47(2175.7to3368.73) | 3346.43(2833.21to4003.95) | 3643.37(2950.44to4479.15) | 3730.25(2721.25to4709.06) | 1.67 (1.49 to 1.86) | 1.05 (0.86 to 1.24) |
| Monaco | 396.53(306.46to497.86) | 344.83(270.33to425.79) | 321.77(264.19to384.9) | 294.54(238.39to352.7) | -1.19 (-1.31 to -1.08) | -0.99 (-1.07 to -0.91) |
| Mongolia | 2171.12(1761.83to2648.35) | 2499.19(2031.66to3038.9) | 1840.72(1465.51to2260.45) | 1621.4(1225.95to2060.28) | -1.26 (-1.85 to -0.66) | -1.75 (-2.07 to -1.43) |
| Montenegro | 922.31(787.39to1061.34) | 1057.4(918.94to1204.44) | 1091.67(941.95to1245.1) | 1031.68(850.35to1235.49) | 1.1 (0.9 to 1.31) | 0.49 (0.3 to 0.69) |
| Morocco | 1536.04(1304.98to1808) | 1655.61(1394.8to1945.3) | 1861.68(1514.32to2264.47) | 2034.54(1634.48to2399.18) | 0.93 (0.83 to 1.03) | 1.06 (0.98 to 1.13) |
| Mozambique | 864.86(721.38to1031.34) | 937.09(783.57to1101.84) | 1061.18(877.93to1257.54) | 1059.16(843.21to1323.89) | 1.04 (0.93 to 1.14) | 0.97 (0.87 to 1.06) |
| Myanmar | 1959.83(1576.47to2434.94) | 1876.62(1541.48to2253.58) | 1680.36(1425.37to1987.55) | 1566.85(1308.96to1876.75) | -0.69 (-0.79 to -0.58) | -0.92 (-1 to -0.83) |
| Namibia | 1107.15(886.8to1387.46) | 1299.56(1053.57to1617.38) | 1094.14(872.52to1368.87) | 1091.27(846.51to1418.4) | 0.28 (-0.19 to 0.75) | -0.31 (-0.6 to -0.03) |
| Nauru | 3030.62(2414.06to3738.62) | 3978.5(3212.78to4823.5) | 4102.39(3254.68to5096.5) | 3869.29(3112.94to4758.6) | 1.75 (1.42 to 2.09) | 0.74 (0.42 to 1.06) |
| Nepal | 885.12(710.67to1101.15) | 862.71(729.36to1024.7) | 912.08(757.1to1077.89) | 1100.3(862.66to1349.7) | -0.2 (-0.4 to -0.01) | 0.67 (0.41 to 0.93) |
| Netherlands | 501.14(415.97to593.48) | 446.67(379.63to520.9) | 321.38(278.16to367.3) | 306.51(267.68to349.65) | -2.29 (-2.59 to -1.98) | -2.06 (-2.24 to -1.88) |
| New Zealand | 661.85(555.03to779.68) | 560.96(481.46to651.51) | 464.11(405.77to526.82) | 437.53(382.48to494.22) | -1.64 (-1.74 to -1.53) | -1.57 (-1.65 to -1.48) |
| Nicaragua | 1548.56(1420.58to1682.26) | 1967.08(1820.14to2134.01) | 2614.4(2448.85to2793.38) | 2710.43(2264.76to3211.68) | 2.67 (2.43 to 2.9) | 2.17 (1.95 to 2.38) |
| Niger | 1282.1(1047.77to1530.36) | 1130.24(927.91to1367.03) | 1049.62(844.63to1266.6) | 1077.37(846.29to1377.67) | -0.88 (-0.99 to -0.77) | -0.6 (-0.71 to -0.49) |
| Nigeria | 1044.05(856.68to1270.89) | 1007.72(797.58to1259.39) | 951.82(758.24to1165.01) | 921.71(735.6to1122.75) | -0.45 (-0.52 to -0.39) | -0.43 (-0.46 to -0.39) |
| Niue | 2002.6(1629.35to2473.68) | 2387.06(1931.42to2888.75) | 2429.71(1966.72to2910.01) | 2399.51(1892.69to2985.38) | 1.04 (0.82 to 1.25) | 0.46 (0.27 to 0.65) |
| North Macedonia | 1064.11(909.54to1234.62) | 1279.02(1092.68to1476.11) | 1237.75(1061.48to1421.54) | 1146.05(902.93to1428.24) | 0.73 (0.42 to 1.04) | -0.04 (-0.29 to 0.22) |
| Northern Mariana Islands | 1972.43(1684.17to2346.3) | 2152.18(1898.35to2479.34) | 2261.05(1965.68to2605.12) | 2287.03(1916.15to2670.1) | 0.59 (0.5 to 0.68) | 0.63 (0.55 to 0.72) |
| Norway | 464.78(375.74to564) | 395.48(327.37to470.46) | 305.81(261.44to356.26) | 258.82(221.14to299.32) | -2.17 (-2.31 to -2.04) | -2.19 (-2.26 to -2.12) |
| Oman | 1705.93(1320.17to2182.56) | 1997.27(1635.61to2431.95) | 2039.64(1712.81to2364.6) | 1818.82(1520.88to2162.98) | 0.82 (0.65 to 1) | 0.33 (0.15 to 0.51) |
| Pakistan | 1248.23(1024.91to1523.84) | 1674.45(1375.7to2013.54) | 1852.55(1493.65to2250.23) | 1831.8(1452.81to2248.31) | 2.06 (1.79 to 2.33) | 1.27 (1.01 to 1.52) |
| Palau | 2654.73(2146.7to3354.92) | 3196.22(2570.88to3910.45) | 3306.53(2676.17to4043.88) | 3209.42(2527.8to3934.15) | 1.25 (1.04 to 1.45) | 0.63 (0.44 to 0.83) |
| Palestine | 2160.96(1727.72to2691.65) | 2074.67(1799.55to2387.27) | 2019.58(1794.52to2252.25) | 1818.17(1535.3to2123.75) | -0.34 (-0.4 to -0.29) | -0.64 (-0.73 to -0.55) |
| Panama | 890.87(784.67to1010.29) | 977.53(883.06to1077.2) | 1064.41(966.3to1174.18) | 1058.39(846.87to1311.67) | 1.02 (0.88 to 1.16) | 0.66 (0.54 to 0.78) |
| Papua New Guinea | 969.67(773.03to1204.61) | 1079.84(837.31to1374.55) | 1178.59(911.73to1497.37) | 1204.26(933.63to1556.81) | 1.08 (1.01 to 1.15) | 0.79 (0.69 to 0.88) |
| Paraguay | 774.12(674.64to885.2) | 919.01(821.48to1014.97) | 1159.87(1051.21to1283.79) | 1241.62(981.59to1558.35) | 2.01 (1.86 to 2.15) | 1.94 (1.83 to 2.04) |
| Peru | 872.95(760.46to992.74) | 848.23(734.35to972.06) | 862.54(740.91to996.93) | 751.6(585.99to949.38) | -0.4 (-0.64 to -0.16) | -0.46 (-0.61 to -0.32) |
| Philippines | 1339.23(1211.52to1482.06) | 1507.94(1346.62to1687.42) | 1978.98(1773.37to2216.64) | 2021.7(1701.03to2396.23) | 2.29 (2.08 to 2.5) | 1.94 (1.77 to 2.11) |
| Poland | 1056.26(869.27to1261.37) | 850.3(713.53to998.52) | 634.54(538.55to743.21) | 511.94(414.03to618.03) | -2.9 (-3.14 to -2.66) | -3 (-3.14 to -2.86) |
| Portugal | 683.99(595.17to779.15) | 586.99(517.47to657.64) | 503.33(450.5to557.28) | 380.29(335.49to426.49) | -1.37 (-1.52 to -1.22) | -2.12 (-2.35 to -1.9) |
| Puerto Rico | 1172.52(1052.74to1302.73) | 1026.49(925.84to1143.12) | 1018.51(919.24to1126.64) | 1015.89(828.22to1254.81) | -0.5 (-0.7 to -0.3) | -0.3 (-0.42 to -0.18) |
| Qatar | 2171.4(1770.97to2675.21) | 2302.89(1907.94to2762.76) | 2152.79(1792.21to2547.14) | 1750.48(1384.13to2183.41) | 0.16 (-0.26 to 0.58) | -0.76 (-1.08 to -0.44) |
| Republic of Korea | 896.3(779.62to1017.86) | 557.18(493.9to621.14) | 388.61(342.83to435.98) | 324.94(286.59to368.54) | -4.03 (-4.18 to -3.88) | -3.52 (-3.7 to -3.34) |
| Republic of Moldova | 921.61(631.35to1234.48) | 1058.13(759.73to1397.15) | 1045.3(772.49to1352) | 812.68(610.33to1036.84) | -0.14 (-0.63 to 0.35) | -0.82 (-1.13 to -0.52) |
| Romania | 948.33(787.02to1121.65) | 922.24(768.64to1085.36) | 802.39(677.04to935.46) | 766.35(614.75to950.88) | -1.19 (-1.53 to -0.84) | -1.17 (-1.36 to -0.99) |
| Russian Federation | 1228.44(975.1to1514.47) | 1598.88(1274.58to1958.11) | 1323.85(1053.55to1612.98) | 1037.16(822.44to1277.18) | 0.19 (-0.54 to 0.92) | -1.17 (-1.66 to -0.66) |
| Rwanda | 1262.63(1061.76to1476.51) | 1156.31(974.04to1343.48) | 835.87(702.71to979.72) | 850.84(686.36to1034.8) | -2.71 (-3.15 to -2.27) | -2.11 (-2.41 to -1.81) |
| Saint Kitts and Nevis | 2394.93(2133.12to2666.02) | 2023.51(1790.62to2263.42) | 1789(1554.79to2008.13) | 1977.23(1619.52to2352.99) | -1.12 (-1.28 to -0.96) | -0.67 (-0.84 to -0.51) |
| Saint Lucia | 1427.73(1292.09to1583.91) |  | 1213.42(1109.62to1325.7) | 1340.7(1151.7to1559.23) | -0.81 (-1.02 to -0.61) | -0.21 (-0.42 to -0.01) |
| Saint Vincent and the Grenadines | 1302.96(1149.32to1472.2) | 1323.26(1191.09to1473.09) | 1391.92(1243.35to1538) | 1550.44(1350.58to1791.5) | 0.21 (0.08 to 0.33) | 0.66 (0.52 to 0.8) |
| Samoa | 2011.4(1627.42to2461.1) | 2280.57(1846.52to2826.4) | 2349.61(1933.77to2823.04) | 2327.09(1851.28to2894.29) | 0.87 (0.74 to 1.01) | 0.44 (0.31 to 0.58) |
| San Marino | 272.19(221.03to329.8) | 232.43(191.03to280.02) | 229.73(174.1to297.76) | 225.42(166.53to298.56) | -0.79 (-0.98 to -0.61) | -0.51 (-0.63 to -0.39) |
| Sao Tome and Principe | 1445.33(1166.49to1666.8) | 1701.74(1473.97to1948.29) | 1717.22(1482.01to1948.64) | 1721.3(1402.63to2002.75) | 0.83 (0.56 to 1.1) | 0.47 (0.31 to 0.63) |
| Saudi Arabia | 2040.63(1654.53to2456.39) | 2411.42(2134.03to2696.1) | 2683.61(2348.5to3017.43) | 2369.66(1935.61to2834.37) | 1.46 (1.38 to 1.55) | 0.62 (0.38 to 0.87) |
| Senegal | 1360.85(1094.48to1657.9) | 1126.28(897.69to1428.94) | 1153.9(939.88to1395.49) | 1168.16(907.09to1467.76) | -0.49 (-0.8 to -0.17) | -0.41 (-0.57 to -0.25) |
| Serbia | 1055.16(897.85to1232.2) | 1165.08(986.31to1355.23) | 1054.04(902.83to1210.64) | 960.63(764.25to1194.47) | -0.06 (-0.4 to 0.27) | -0.58 (-0.8 to -0.37) |
| Seychelles | 1472.18(1297.69to1666.39) | 1701.55(1536.47to1888.48) | 1655.11(1505.3to1817.98) | 1681.31(1477.84to1887.74) | 0.61 (0.32 to 0.9) | 0.16 (-0.02 to 0.34) |
| Sierra Leone | 1197.17(961.78to1452.34) | 1096.72(889.05to1321.88) | 1190.71(966.17to1425.73) | 1149.55(900.13to1455.3) | 0.16 (-0.06 to 0.38) | 0.09 (-0.02 to 0.2) |
| Singapore | 1002.71(868.71to1145.95) | 672.39(571.56to784.59) | 532.82(467.33to608.78) | 452.75(394.46to513.66) | -3.17 (-3.35 to -2.98) | -2.62 (-2.79 to -2.44) |
| Slovakia | 1131.15(921.74to1358.91) | 986.81(810.46to1170.75) | 814.99(670.3to966.16) | 715.14(555.6to906.96) | -1.32 (-1.48 to -1.17) | -1.68 (-1.81 to -1.55) |
| Slovenia | 536(410.64to706.99) | 433.38(355.73to518.78) | 336.03(283.42to395.63) | 303.96(238.49to378.66) | -2.39 (-2.5 to -2.29) | -2.21 (-2.31 to -2.1) |
| Solomon Islands | 3377.4(2592.39to4392.05) | 3663.93(2883.2to4589.63) | 3212.38(2505.8to4038.79) | 3124.76(2412.92to3912.13) | -0.39 (-0.67 to -0.11) | -0.39 (-0.54 to -0.24) |
| Somalia | 1195.34(922.14to1488.72) | 1140.56(862.08to1442.1) | 1184.71(899.07to1497.57) | 1147.43(880.04to1467.76) | -0.03 (-0.14 to 0.07) | 0 (-0.06 to 0.05) |
| South Africa | 942.83(853.97to1038.81) | 1282.64(1185.6to1396.89) | 1248.65(1151.13to1349.73) | 1094.43(1001.47to1193.94) | 1.55 (1.09 to 2.02) | 0.57 (0.22 to 0.93) |
| South Sudan | 947.78(746.68to1193.73) | 897.84(679.27to1131.74) | 890.32(664.27to1148.7) | 885.68(667.78to1130.82) | -0.42 (-0.55 to -0.29) | -0.23 (-0.31 to -0.15) |
| Spain | 561.37(492.54to631.36) | 427.72(374.02to482.36) | 332.84(291.64to374.69) | 282.52(246.97to319.55) | -2.34 (-2.45 to -2.23) | -2.38 (-2.45 to -2.31) |
| Sri Lanka | 1285.41(1113.4to1476.19) | 1360.67(1206.24to1524.44) | 1263.51(1124.85to1420.31) | 1145.29(887.66to1466.35) | 0.25 (0 to 0.51) | -0.23 (-0.42 to -0.04) |
| Sudan |  | 1713.76(1364.02to2123.07) | 1702.61(1360.21to2106.54) | 1801.41(1437.25to2253.74) | -0.15 (-0.18 to -0.13) | 0.04 (-0.03 to 0.11) |
| Suriname | 1450.55(1263.96to1626.88) | 1625.82(1474.22to1773.69) | 1667.09(1534.91to1807.38) | 1828.8(1547.02to2137.41) | 0.78 (0.31 to 1.25) | 0.71 (0.47 to 0.94) |
| Sweden | 511.29(392.56to646.35) | 410.25(327.08to503.58) | 338.66(281.33to404.43) | 307.22(257.06to363.89) | -2.07 (-2.12 to -2.02) | -1.79 (-1.87 to -1.71) |
| Switzerland | 436.61(355.69to521.11) | 371.5(307.04to440.49) | 312.38(265.35to360.83) | 284.43(240.5to328.39) | -1.79 (-1.87 to -1.72) | -1.55 (-1.63 to -1.48) |
| Syrian Arab Republic | 2143.09(1755.17to2596.11) | 2325.47(1945.74to2732.87) | 1970.34(1586.17to2380.7) | 2033.76(1570.32to2622.22) | -0.84 (-1.18 to -0.49) | -0.56 (-0.75 to -0.38) |
| Taiwan (Province of China) | 1078.03(981.7to1181.18) | 867.64(786.13to959.78) | 768.47(689.64to855.52) | 806.35(669.96to968.5) | -1.55 (-1.7 to -1.41) | -1 (-1.18 to -0.82) |
| Tajikistan | 740.82(557.59to947.57) | 904.44(673.71to1177.56) | 1157.91(865.92to1512.8) | 1293.27(940.12to1688.78) | 1.79 (1.52 to 2.06) | 2.01 (1.84 to 2.17) |
| Thailand | 1285.1(1133.04to1458.31) | 1355.62(1179.19to1554.28) | 1088.19(965.14to1226.76) | 1054.74(836.11to1323.54) | -1.11 (-1.42 to -0.79) | -1.11 (-1.28 to -0.95) |
| Timor-Leste | 1360.41(1102.6to1658.98) | 1300.09(975.94to1597.65) | 1329.87(1000.47to1605.16) | 1559.67(1178.83to1883.49) | -0.23 (-0.35 to -0.1) | 0.51 (0.3 to 0.72) |
| Togo | 1237.33(1020.11to1490.5) | 1199.83(993.51to1451.79) | 1241.62(1010.6to1497.25) | 1181.14(949.32to1476.22) | -0.04 (-0.12 to 0.04) | -0.11 (-0.15 to -0.06) |
| Tokelau | 1761.03(1385.85to2199.14) | 2001.3(1583.66to2535.15) | 2065.84(1647.8to2603.08) | 2049.06(1592.87to2653.65) | 0.94 (0.79 to 1.1) | 0.5 (0.36 to 0.64) |
| Tonga | 1351.61(1132.27to1634.05) | 1709.66(1440.05to2010.32) | 1735.12(1435.96to2102.92) | 1771.75(1439.73to2224.26) | 1.53 (1.19 to 1.87) | 0.9 (0.67 to 1.14) |
| Trinidad and Tobago | 1319.06(1152.24to1496.88) | 1514.21(1349.58to1688.98) | 1494.48(1368.05to1629.28) | 1489.85(1151.48to1891.8) | 0.58 (0.32 to 0.85) | 0.3 (0.15 to 0.45) |
| Tunisia | 1252.58(1065.84to1468.31) | 1378.27(1111.51to1673.28) | 1389.51(1087.5to1771.79) | 1364.05(1044.93to1730.82) | 0.44 (0.33 to 0.56) | 0.23 (0.15 to 0.31) |
| Turkey | 1472.82(1247.09to1762.25) | 1223.08(1047.42to1428.68) | 1151.13(991.51to1328.57) | 1075.07(880.13to1313.94) | -1.48 (-1.64 to -1.32) | -1.03 (-1.18 to -0.88) |
| Turkmenistan | 1475.05(1197.61to1782.13) | 1753.27(1414.77to2124.96) | 1657.76(1382.2to1955.22) | 1762.58(1361.02to2219.01) | 0.61 (0.08 to 1.15) | 0.07 (-0.23 to 0.37) |
| Tuvalu | 2232.39(1803.93to2736.97) | 2488.17(2052.28to3004.92) | 2579.47(2037.55to3234.89) | 2597.83(1981.57to3376.69) | 0.83 (0.72 to 0.94) | 0.52 (0.42 to 0.62) |
| Uganda | 837.58(683.35to1014.24) | 984.31(807.4to1206.8) | 884.74(724.86to1056.63) | 864.02(705.62to1047.68) | 0.19 (-0.13 to 0.52) | -0.16 (-0.34 to 0.02) |
| Ukraine | 875.8(625.01to1160.08) | 1125.69(771.33to1507.1) | 1083.01(757.8to1447.82) | 1173.67(841.32to1574.82) | 1.09 (0.66 to 1.52) | 0.48 (0.17 to 0.78) |
| United Arab Emirates | 2537.37(2142.12to2959.14) | 2942.53(2470.61to3454.74) | 2802.28(2304.86to3335.9) | 2074.42(1614.2to2673.46) | 0.76 (0.4 to 1.12) | -0.82 (-1.3 to -0.33) |
| United Kingdom | 536(435.66to642.41) | 412.68(338.76to489.77) | 300.38(253.06to352.03) | 279.57(239.25to324.61) | -2.95 (-3.07 to -2.84) | -2.6 (-2.76 to -2.44) |
| United Republic of Tanzania | 926.16(787.94to1074.05) | 896.74(768.81to1038.47) | 940.9(809.48to1082.18) | 965.55(815.25to1142.41) | 0 (-0.14 to 0.14) | 0.22 (0.13 to 0.31) |
| United States of America | 786.8(663.79to918.6) | 793.04(685.21to906.5) | 711.31(632.97to794.71) | 724.69(646.79to808.11) | -0.29 (-0.47 to -0.1) | -0.32 (-0.42 to -0.22) |
| United States Virgin Islands | 1162.79(984.51to1374.22) | 1248.09(1073.82to1443.65) | 1484.49(1301.65to1669.64) | 1447.3(1222.99to1676.01) | 1.34 (1.12 to 1.56) | 1.07 (0.92 to 1.22) |
| Uruguay | 696.42(606.05to796.46) | 605.19(536.59to676.3) | 545.97(487.19to607.5) | 539.25(483.14to598.34) | -0.99 (-1.15 to -0.83) | -0.86 (-0.95 to -0.77) |
| Uzbekistan | 1391.73(1152.85to1653.54) | 2274.37(1896.17to2708.59) | 2740.73(2216.57to3306.97) | 2461.89(1924.89to3100.03) | 3.47 (2.98 to 3.96) | 1.86 (1.36 to 2.37) |
| Vanuatu | 1815.91(1354.55to2373.15) | 2185.73(1685.05to2838.86) | 2463.43(1907.02to3170.78) | 2640.98(2039.04to3423.93) | 1.18 (0.97 to 1.39) | 1.17 (1.06 to 1.28) |
| Venezuela (Bolivarian Republic of) | 1168.65(1006.5to1343.47) | 1462.9(1317.65to1624.99) | 1505.72(1372.26to1664.4) | 1619.37(1258.08to2071.35) | 1.44 (1.18 to 1.69) | 0.8 (0.59 to 1.01) |
| Viet Nam | 1250.97(1029.69to1511.02) | 1050.6(899.88to1227.59) | 1125.76(961.95to1308.13) | 1207.49(984.42to1454.33) | -0.56 (-0.85 to -0.27) | 0.05 (-0.17 to 0.27) |
| Yemen | 1629.61(1269.89to2098.99) | 1573.56(1225.76to2001.27) | 1566.32(1251.3to1972.1) | 1715.51(1368.18to2176.55) | -0.17 (-0.23 to -0.12) | 0.13 (0.03 to 0.23) |
| Zambia | 1158.16(995.64to1343.06) | 1303.98(1109.22to1498.21) | 1214.03(1031.04to1417.18) | 1146.7(932.96to1402.48) | 0.13 (-0.11 to 0.37) | -0.27 (-0.43 to -0.11) |
| Zimbabwe | 959.71(780.05to1226.88) | 1149.41(921.11to1477.13) | 1397.89(1118.9to1831.97) | 1381.13(1068.88to1818.86) | 2.31 (2.07 to 2.55) | 1.61 (1.38 to 1.84) |

ASDR, age-standard DALYs rate; DALYs, disability-adjusted life years; EAPC, estimated annual percentage change.
